# Design, development, and testing of a new multi-locus sequence typing scheme for the zoonotic pathogen *Cryptosporidium parvum*

**DOI:** 10.1016/j.crpvbd.2025.100308

**Published:** 2025-08-14

**Authors:** Karin Troell, Christen Rune Stensvold, Anna Rosa Sannella, Martha Betson, Emma Östlund, Rachel M. Chalmers, Umer Chaudhry, Rebecca Davidson, Lauren Davies, Ralf Ignatius, Anton de Jong, Gregory Karadjian, Karim Adjou, Christian Klotz, Sokratis Ptochos, Guy Robinson, Jeroen Roelfsema, Barbara Soba, Jacek Sroka, Paolo Vatta, Jonas Johansson Wensman, Simone M. Cacciò

**Affiliations:** a Swedish Veterinary Agency, Uppsala, Sweden; b Norwegian Veterinary Institute, Ås, Norway; c Statens Serum Institut, Copenhagen, Denmark; d Department of Infectious Diseases, Istituto Superiore di Sanità, Rome, Italy; e Discipline of Comparative Biomedical Sciences, School of Veterinary Medicine, University of Surrey, Guildford, UK; f Cryptosporidium Reference Unit, Public Health Wales Microbiology, Singleton Hospital, Swansea, UK; g Swansea University Medical School, Singleton Park, Swansea, UK; h Department of Veterinary Biomedical Sciences, Lewyt College of Veterinary Medicine, Long Island University, Brookville, NY, USA; i Department of Animal Health, Welfare and Food Safety, Norwegian Veterinary Institute, Tromsø, Norway; j MVZ Labor 28, and Department of Microbiology and Infection Immunology, Charité- Universitätsmedizin Berlin, Germany; k UMR BIPAR, Anses, Laboratoire de Santé Animale, INRAE, École Nationale Vétérinaire d’Alfort, Maisons-Alfort, France; l Department of Infectious Diseases, Unit 16 Mycotic and Parasitic Agents and Mycobacteria, Robert Koch-Institute, Berlin, Germany; m National Institute for Public Health and Environment (RIVM), Bilthoven, the Netherlands; n Institute of Microbiology and Immunology, Faculty of Medicine, University of Ljubljana, Ljubljana, Slovenia; o Department of Parasitology and Invasive Diseases, Bee Diseases and Aquatic Animal Diseases, National Veterinary Research Institute, Puławy, Poland; p Department of Animal Biosciences, Swedish University of Agricultural Sciences (SLU), Uppsala, Sweden

**Keywords:** *Cryptosporidium parvum*, Whole genome sequences, Multi-locus sequence typing scheme, Population structure, *gp60*, Outbreak investigation, Source tracking

## Abstract

The zoonotic parasite *Cryptosporidium parvum* is an important global cause of diarrheal disease in humans and young ruminants. Molecular typing is essential to track transmission routes and identify clusters of cases. Here, we developed a novel multi-locus sequence typing (MLST) scheme based on single nucleotide polymorphisms (SNPs) in unlinked markers. Coding regions with high variability were identified by comparing whole genome sequences (WGS) from 43 human- and 92 ruminant-derived *C. parvum* samples collected across Europe. We first selected 18 markers and showed that they provide high discrimination among the samples with WGS data, with 88% of the MLSTs being singletons. Next, we defined a MLST based on eight genetically unlinked markers and generated sequence data from 305 *C. parvum* samples, collected from four different host species and 13 European countries. We consolidated a set of 365 fully genotyped samples, characterized by the presence of 154 different MLSTs, 105 of which were singletons. Network analyses showed no complete clustering of samples by host species or country of origin at the European scale. We further showed that samples with *gp60* subtypes that are common in Europe are divided into many MLSTs by the new scheme, highlighting its increased discriminatory ability. However, the applicability of the scheme in public health settings is limited by its cost, turnaround time, and scalability. To achieve discrimination of *C. parvum* samples based on SNPs, a large number of loci needs to be analysed, and this is feasible using amplicon sequencing technologies.

## Highlights

• *Cryptosporidium parvum* whole genome sequences were mined to identify novel markers.
• A novel multi-locus sequence typing (MLST) scheme was developed and tested on more than 300 samples.
• Discriminatory power of the new MLST scheme was evaluated on outbreak and background cases in Europe.
• Comparison with *gp60* sequence data highlights the increased discriminatory ability of the new MLST scheme.

## Graphical abstract

**Figure:**
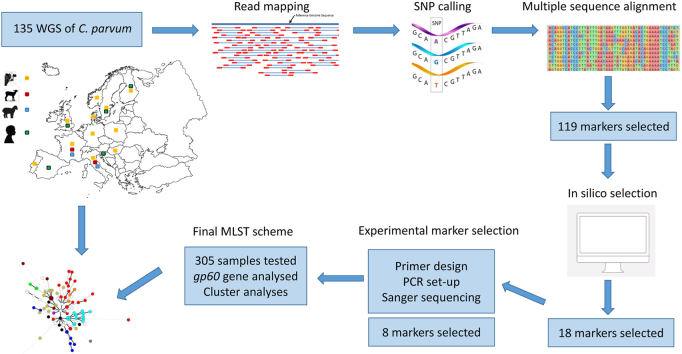

## 1 Introduction

The apicomplexan parasite *Cryptosporidium parvum* is one of the many species described in the genus *Cryptosporidium*, and by far the most important in terms of zoonotic potential (Ryan et al., 2021). It is primarily a pathogen of humans and young ruminants and a global cause of diarrheal disease in these hosts (Gibson and Striepen, 2018; Guo et al., 2021). Both direct (human-to-human, animal-to-human) and indirect (through ingestion of contaminated water or food) transmission routes exist, resulting in a complex epidemiology (Zahedi and Ryan, 2020). Molecular methods are necessary to understand transmission routes, epidemiologic patterns, and population structure (Feng et al., 2018).

Largely, molecular characterization of *Cryptosporidium* has been based on the analysis of single-nucleotide polymorphisms (SNPs) in different gene markers (Bouzid et al., 2010) or on length polymorphisms in loci containing simple sequence repeats (Widmer et al., 2004). Among gene markers, the one encoding the 60 kDa glycoprotein (*gp60*) is extremely polymorphic in *C. parvum* (Strong et al., 2000) and therefore a commonly used marker for molecular epidemiologic studies (Xiao and Feng, 2017; Chalmers et al., 2019; Robinson et al., 2025). The availability of an accepted nomenclature (Alves et al., 2006; Robinson et al., 2025) has contributed to its wide use. Based on *gp60* sequence analysis, several *C. parvum* subtype families have been described (e.g. IIa and IId), each comprising a number of variants (or subtypes) that differ mainly in the number of trinucleotide repeats (TCA or TCG) present at the 5′-end of the gene (Feng et al., 2018; Robinson et al., 2025). Globally, in both humans and animals, the IIa group (or family) is more prevalent in Europe, the Americas and the Middle East, whereas the IId group predominates in Asia (Feng et al., 2018; Bulumulla et al., 2025). Despite high variability, some subtypes, such as IIaA15G2R1 and IIaA16G2R1, have a widespread geographical distribution and are commonly found in humans and ruminants (Cacciò and Chalmers, 2016). This makes *gp60* a less informative marker in a public health context, as it is not possible to distinguish sporadic cases from epidemic clusters and outbreaks or to infer zoonotic transmission, except when rare *gp60* subtypes are involved. Furthermore, it has been argued that for an organism such as *C. parvum*, which has an obligatory sexual phase in its life cycle, the use of a single marker may not be appropriate, particularly not in areas with high transmission rates and therefore higher chances of recombination (Baptista et al., 2021). Indeed, a growing body of data demonstrated that *gp60* typing cannot be used as a proxy for the genetic identity of the samples, because this locus is often involved in recombination events (Widmer and Sullivan, 2012; Feng et al., 2013).

Another rich source of polymorphism is represented by genomic regions containing tandem repeat sequences, such as micro- and mini-satellites (e.g. Barker, 2002). The variable number of tandem repeats (VNTR) can be determined through amplification followed by sequencing or fragment length analysis and used to compare samples. Many VNTR loci have been investigated, and several multi-locus typing schemes have been proposed (Mallon et al., 2003; Gatei et al., 2007; Feng et al., 2011) and applied to different *Cryptosporidium* species. This has revealed parasite population structures ranging from panmictic to clonal, likely resulting from ecological factors such as transmission intensity (Morrison et al., 2008; Tanriverdi et al., 2008; Drumo et al., 2012). However, these schemes have rarely been compared in terms of reproducibility and ability to discriminate samples in different epidemiologic contexts (Widmer and Sullivan, 2012; Chalmers and Cacciò, 2016).

Recently, the availability of whole genome sequences (WGS) has allowed the first in-depth analyses of important aspects of *Cryptosporidium* biology, including evolution, host adaptation and population structure (Wang et al., 2022; Corsi et al., 2023; Bellinzona et al., 2024).

The search for novel loci containing VNTR has also been facilitated by mining of WGS data (Pérez-Cordón et al., 2016), and a scheme based on seven markers has been validated and shown to be informative for use in public health investigations in the UK, Sweden, Finland, and France (Robinson et al., 2022; Risby et al., 2023; Suominen et al., 2025; Chalmers et al., 2025).

In this work, we analysed 135 WGS of human- and ruminant-derived *C. parvum* samples collected across Europe to identify coding regions with a high number of single-nucleotide polymorphisms (SNPs). Our aim was to develop a new multi-locus sequence typing (MLST) scheme based on eight markers, one per chromosome, and to assess its discriminatory power on a collection of 305 human and ruminant *C. parvum* samples collected across Europe.

## 2 Materials and methods

### 2.1 Parasite samples

This work was conducted within the framework of the PARADISE project, part of the One Health European Joint Programme (One Health EJP). The 135 *C. parvum* samples for which WGS data were available have been described in previous publications (Hadfield et al., 2015; Corsi et al., 2023; Bellinzona et al., 2024). Samples of genomic DNA extracted from 305 human- and ruminant-derived *C. parvum* fecal samples from 13 European countries were also used (“field samples”). Table 1 lists the country of origin and the host species from which the 440 samples were collected. Additional information about the samples is given in Supplementary file 1: Table S1.

**Table 1:** The samples included in the study, with information on the host species and geographical origin.

Country	No. of samples with existing WGS data (host)	No. of field samples (host)
Czech Republic	0	6 (cattle)
Denmark	9 (cattle)	23 (cattle); 22 (human)
Finland	10 (cattle); 7 (human)	0
France	2 (cattle); 3 (sheep); 2 (goats)	10 (cattle); 10 (sheep); 4 (goats)
Germany	16 (cattle)	12 (human)
Hungary	11 (cattle)	0
Italy	12 (cattle); 10 (sheep); 5 (goats)	0
Latvia	0	4 (cattle)
Netherlands	0	17 (human)
Norway	4 (cattle)	9 (cattle)
Poland	2 (cattle)	14 (cattle)
Portugal	1 (cattle)	11 (cattle)
Slovenia	6 (human)	15 (human)
Spain	1 (human)	0
Sweden	3 (cattle); 2 (human)	103 (cattle); 6 (human)
UK	2 (cattle); 27 (human)	11 (cattle); 2 (sheep); 26 (human)
Total	72 (cattle); 13 (sheep); 7 (goats); 43 (human)	191 (cattle); 12 (sheep); 4 (goats); 98 (human)
Grand total	135	305

### 2.2 Identification of candidate markers from WGS data

The overall bioinformatics process is schematically presented in Supplementary file 2: Fig. S1. The processing of raw reads is described in the original publications (Corsi et al., 2023; Bellinzona et al., 2024). Briefly, Illumina raw reads (2× 150 bp) were trimmed with Trimmomatic v. 0.38 (Bolger et al., 2014) and their quality was checked before and after trimming using FastQC v.0.11.9 (https://github.com/s-andrews/FastQC/releases). Samples were down-sampled to 100× coverage, based on the size (9.1 Mb) of the *C. parvum* IOWA genome (accession number PRJNA573722) by using the reformat.sh script from BBmap v. 38.79 (https://sourceforge.net/projects/bbmap/files/). The IOWA reference genome and its annotation were used for all subsequent analyses requiring a reference. Kraken2 v.2.0.8 (Wood et al., 2019) was used to estimate the fraction of reads that originated from *C. parvum*. If a sample was contaminated but contained enough *C. parvum* reads to achieve 10× genome coverage, it was de-contaminated by mapping the reads to the reference genome using Bowtie2 v.2.3.5.1 (Langmead and Salzberg, 2012), and the mapped reads were subsequently extracted with Samtools v.1.9 (Danecek et al., 2021).

Processed samples were analysed using a pipeline developed during the COMPARE project (www.compare-europe.eu), which is available at https://github.com/EBI-COMMUNITY/ebi-parasite. Briefly, the pipeline includes steps for assembly, mapping of reads to a reference genome, variant calling, identification of repeated sequences, and analysis of variability within coding sequences.

An *ad hoc* script was used to extract all within-gene fragments of up to 500 bp in length with at least two SNPs in multiple samples and a Simpson’s index not greater than 0.35. Simpson’s index was calculated using the script available at https://gist.github.com/martinjc.

Next, the selected gene fragments were ranked based on the number of SNPs, and multiple alignments were generated for each candidate marker. To allow the design of primer sequences for PCR amplification, 100 bases on both the 5′ and 3′ flanking regions of each marker were included. Primer design was performed using the Primer-BLAST tool (Ye et al., 2012) available at the NCBI website. The overall bioinformatics process is schematically presented in Supplementary file 2: Fig. S1.

### 2.3 Laboratory tests and markers selection

The 18 markers selected as described above were initially tested in a single laboratory (Swedish Veterinary Agency, SVA). Five DNA samples, extracted from *C. parvum*-positive feces and previously tested at RIVM using a *C. parvum-*specific qPCR (Hadfield et al., 2011), with observed Ct-values ranging from 26.73 to 38.72, were used. Primers were designed for single PCR amplification, and reactions were evaluated in terms of sensitivity and reproducibility, and subsequently, for the quality of Sanger sequencing of the amplification products.

### 2.4 PCR and sequencing from *C. parvum* field samples

In order to increase sensitivity, nested primers were designed for the eight markers selected to be included in the final scheme, whereas the outer primers were those designed for the single PCR amplifications described above. All primer sequences are listed in Supplementary file 3. The DNA extracted from 305 *C. parvum*-positive fecal samples was amplified by PCR and the products sequenced in both directions. These experiments were performed at the following laboratories: Istituto Superiore di Sanità, Swedish Veterinary Agency, Public Health Agency, Sweden, University of Surrey, University of Ljubljana, Statens Serum Institut, and Robert Koch Institute.

### 2.5 Clustering analysis

Each distinct sequence (i.e. each allele) at each marker was allocated an integer, and this was repeated for all eight markers. Next, the allele numbers at each marker were concatenated to define the multi-locus sequence type (MLST) that characterized each sample. The MLSTs were then imported along with available metadata into BioNumerics (v.7.6.3, Applied Maths, Belgium) to generate minimum spanning trees.

### 2.6 Statistical analysis

Typeability (T) was assessed for each of the eight gene markers individually and for the eight-locus scheme as a whole, using the full set of 440 samples, including data from both WGS and field samples. T was calculated as the number of samples assigned to an MLST within the total number of samples tested.

The discriminatory power (D), defined as the probability that two unrelated samples will be allocated to different MLST, was assessed using the Hunter-Gaston Discriminatory Index (HGDI) (Hunter and Gaston, 1988). For this, a subset of 308 epidemiologically unrelated samples with complete genotyping data was selected from within the overall dataset.

## 3 Results

### 3.1 *In silico* selection of candidate markers from WGS data

We analysed 135 WGS from 43 human- and 92 ruminant-derived *C. parvum* samples (Table 1 and Supplementary file 1: Table S1) to identify coding regions (500–700 bp in length) with a high number of SNPs. Using the analytical workflow detailed in *Section* 2 (see Supplementary file 2: Fig. S1), a total of 150 candidate markers within 119 coding regions was identified (Supplementary file 1: Table S2). The number of candidates was reduced to 69 by discarding markers for which the design of PCR primers was problematic, due to the presence of monotonous or repetitive sequences in the flanking regions (Supplementary file 1: Table S2). Next, we explored *in silico* all possible MLST schemes (always imposing one marker per chromosome) and compared schemes for their relative ability to discriminate the 135 isolates with WGS data (i.e. comparison of how many different types each scheme will generate). This led to the selection of 18 markers (Supplementary file 1: Table S3).

**Fig. 1:**
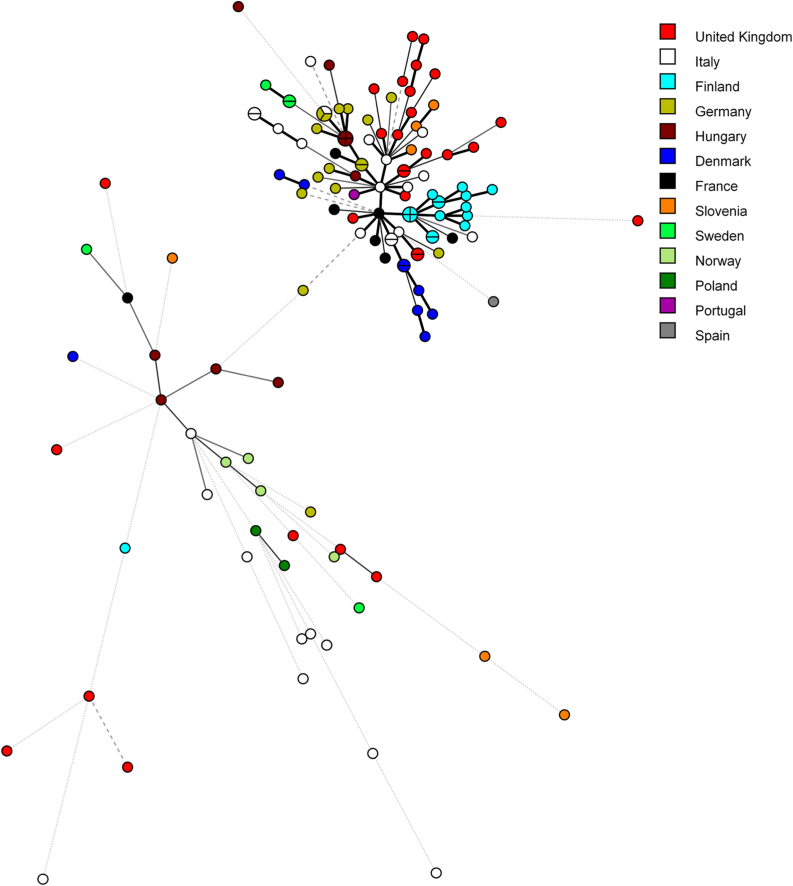
Minimum spanning tree of the 118 MLSTs generated using 18 markers and 135 *C. parvum* samples with WGS data. Samples were obtained from 13 European countries, identified with different colors in the figure, as indicated in the legend. The size of the circles is proportional to the relative frequency of the corresponding MLST. Branch styles correspond to the relationship between samples: thick solid line for one locus variants; thinner solid line for two or three locus variants; dashed line for four locus variants; and dotted line for five locus variants and above.

As shown in Fig. 1, the combination of alleles from the 18 markers found among the 135 samples with WGS data identified 118 different MLSTs (see also Supplementary file 1: Table S4). The majority of these MLSTs (104 of 118, 88%) were singletons, whereas identical MLSTs were found in small numbers of samples, creating MLST clusters. Clusters comprised either two (9 clusters), three (1 cluster), or four (2 clusters) samples, and were formed by samples from Denmark, Finland, Germany, Hungary, Italy, Sweden and the UK. Clusters comprised samples from known outbreaks, single farms, or close localities within a country.

However, when examining the MLST of 15 samples from six different outbreaks that occurred in the UK, or that involved UK residents, we found an allelic difference in one marker on chromosome 8 that distinguished one sample (UKP4) from an outbreak from the two other samples (UKP5 and UKP6) from the same outbreak (outbreak 7) (Supplementary file 1: Table S4). Likewise, the three samples (UKP102, UKP103 and UKP118) from another outbreak (outbreak 1) had identical alleles at 17 markers, but different alleles at another marker on chromosome 8 (Supplementary file 1: Table S4). In the case of a third outbreak (outbreak 3), two samples (UKP90 and UKP121) differed only within a marker on chromosome 8, but a third sample (UKP121) had additional allelic differences within one marker on chromosome 2 and one marker on chromosome 4 (Supplementary file 1: Table S4). Finally, the two samples (UKP104 and UKP122) from a fourth outbreak (outbreak 4) had different alleles within markers on chromosomes 6 and 8 (Supplementary file 1: Table S4).

### 3.2 Laboratory testing of candidate markers

PCR and sequencing experiments were performed on selected DNA with a range of Ct-values in qPCR, thus representing differing amounts of *Cryptosporidium* DNA. No significant differences in the rate of amplification or quality of Sanger sequences among the 18 candidate markers were observed (data not shown). Therefore, we manually curated these candidates to consolidate a scheme comprised of 8 markers, one per chromosome. We took into account their location in the genome, as well as the distribution of the SNPs and the presence of sequence repeats in the amplified fragments, and we selected the eight markers described in Table 2. The marker on chromosome 3 (encoding an alpha-ketoglutarate-dependent dioxygenase AlkB-like protein) contained an intron sequence, while the marker on chromosome 4 (encoding an uncharacterized protein) contained amino acid motif repeats (SKSR), but no variation in the number of repeats was observed among the 135 samples with WGS data.

**Table 2:** The eight selected gene markers, showing the chromosome, chromosomal position, and the encoded protein.

Gene marker	Chromosome (position)	Protein encoded
CPATCC_0039030	1 (790,331–792,574)	Carboxypeptidase A protein with a signal peptide
CPATCC_0028230	2 (467,600–469,861)	Unspecified protein product
CPATCC_0031960	3 (299,687–300,616)	Alpha-ketoglutarate-dependent dioxygenase AlkB-like protein
CPATCC_0021750	4 (1,099,352–1,102,168)	Uncharacterized protein containing SKSR repeats
CPATCC_0024650	5 (644,924–651,805)	Chorein/VPS13-like protein involved in vacuolar transport
CPATCC_0012400	6 (237,331–238,977)	Pescadillo-like protein
CPATCC_0007000	7 (313,585–326,058)	Unspecified protein product
CPATCC_0001010	8 (254,230–260,547)	VPS13-like protein involved in vacuolar protein trafficking

The discriminatory power of these eight markers was tested on the 135 samples with WGS data, revealing 73 different MLSTs, a few of which were relatively common (Supplementary file 2: Fig. S2).

### 3.3 Testing the selected markers on additional *C. parvum*-positive samples

We examined the genetic variability across the eight selected markers by PCR and sequencing of DNA extracted from 305 field samples collected from humans, cattle, sheep, and goats, originating from 13 European countries (Table 1 and Supplementary file 1: Table S1).

As detailed in Table 3, additional SNPs and alleles were identified in field samples at the markers on chromosomes 1, 2, 3, 4 (for this marker, also insertions/deletions of a TCAAGA motif were found), 5, and 8. No additional variability was observed for the markers on chromosomes 6 and 7. The number of alleles ranged from 5 (for the marker on chromosome 7) to 21 (for the marker on chromosome 4). Two largely different sequences were found at the marker on chromosome 4 in samples NL-H29 and NL-H47, collected from humans in the Netherlands (data not shown). Multiple alignments of the allele sequences found at each marker are available in Supplementary file 4.

**Table 3:** Summary of the single nucleotide polymorphisms (SNPs) and alleles found in each marker in the 135 samples with WGS data, of the additional SNPs found in 305 field samples, and of the total number of alleles in the samples studied.

Gene marker	No. of SNPs in samples with WGS data	No. of alleles in samples with WGS data	Additional SNPs in field samples	Total no. of alleles identified
CPATCC_0039030	7	6	5	8
CPATCC_0028230	7	7	2	9
CPATCC_0031960	6	7	2	7
CPATCC_0021750	8	12	2^a^	21^b^
CPATCC_0024650	4	5	2	7
CPATCC_0012400	5	7	0	7
CPATCC_0007000	4	5	0	5
CPATCC_0001010	7	7	3	9

a Including TCAAGA insertion/deletion.

b Including alleles with divergent sequences found in samples NL-H29 and NL-H47.

The typeability (T) index, calculated using the 440 samples with full genotyping data, ranged from 0.94 to 0.99 for individual markers, and was 0.83 for the eight markers combined (Table 4). The Hunter-Gaston discrimination index, calculated on a subset of 308 epidemiologically unrelated samples, ranged from 0.15 to 0.78 for individual markers, and was 0.98 for the eight markers combined (Table 4).

**Table 4:** Typeability and discriminatory index of single markers and the combined MLST scheme.

Marker	Typeability^a^	HGDI^b^
CPATCC_0039030	0.99	0.54
CPATCC_0028230	0.94	0.58
CPATCC_0031960	0.98	0.56
CPATCC_0021750	0.98	0.78
CPATCC_0024650	0.99	0.18
CPATCC_0012400	0.98	0.67
CPATCC_0007000	0.95	0.63
CPATCC_0001010	0.97	0.15
Eight-marker MLST scheme	0.83	0.98

a Calculated using data of 135 samples with WGS data and 305 field samples.

b Hunter-Gaston discrimination index (HGDI) calculated from a subset of 308 epidemiologically unlinked samples.

### 3.4 Typeability and discriminatory power of the new multi-locus sequence typing scheme

Out of the 440 samples tested, sequence data for all eight markers were generated for all 135 samples with WGS data and for 230 of 305 field samples (T = 83%). The remaining 75 samples with no WGS data were excluded because of failure of amplification at one marker (*n* = 40), because the allele could not be assigned unequivocally due to poor or incomplete sequencing results, or for the presence of mixed alleles (*n* = 35) (Supplementary file 1: Table S5). Based on the combination of alleles found among the 365 fully genotyped samples, a total of 154 different MLSTs were identified, 105 of which were singletons (Supplementary file 1: Table S5).

**Fig. 2:**
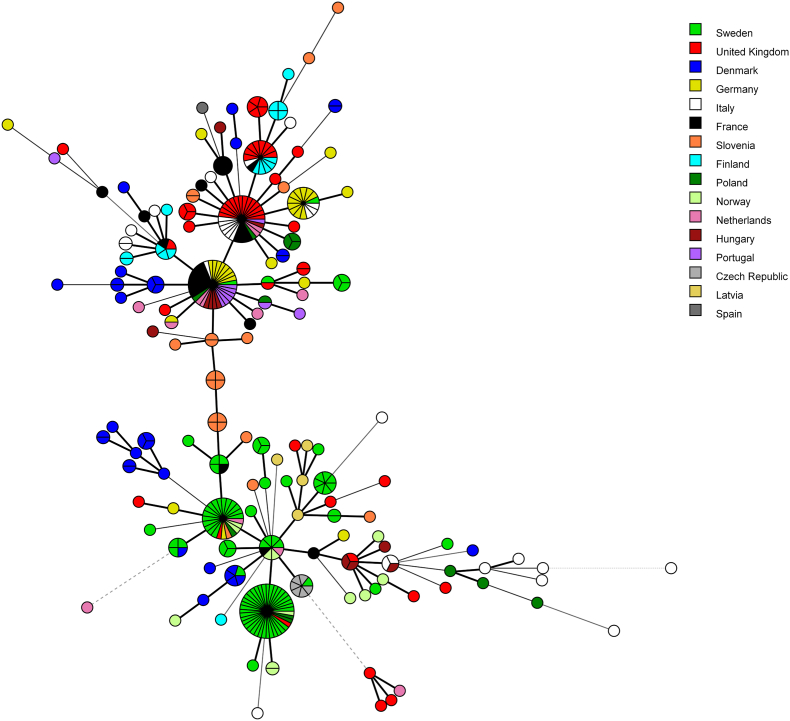
Minimum spanning tree showing the distribution of the 154 MLSTs found in 365 *C. parvum* samples from 16 European countries, which are labelled with different colors, as indicated in the legend. The size of the circles is proportional to the relative frequency of the corresponding MLST. Branch styles correspond to the relationship between samples: thick solid line for one locus variants; thinner solid line for two or three locus variants; dashed line for four locus variants; and dotted line for five locus variants and above.

A minimum spanning tree analysis showed that, at the European scale, there was no complete clustering of MLSTs by country of origin (Fig. 2), by host species (Supplementary file 2: Fig. S3), or by *gp60* family (Supplementary file 2: Fig. S4).

Several MLSTs were found to be widely distributed, including MLST-1, which was found in 31 samples from humans, cattle, sheep and goats collected across 9 countries, MLST-2, which was found in 29 samples from humans, cattle, sheep and goats from 7 countries, and MLST-3, which was found in samples from 20 cattle and two humans in Sweden (Supplementary file 1: Table S5).

The relative variability of the markers (number of alleles per marker) varied in samples from different EU countries (Supplementary file 1: Table S5). For example, among the 93 cattle samples from Sweden, the markers at chromosomes 3 and 6 were the least variable, with allele 1 found in 88 of 93 samples at both markers, whereas the marker at chromosome 4 was the most variable, with 7 different alleles, among which only allele 11 was particularly frequent (in 50% of the samples). In contrast, among the 52 samples from the UK, the markers at chromosomes 5 and 8 were the least variable, with allele 1 found in 49 of 52 samples at both markers. A similar situation was observed in Germany, where the marker at chromosome 5 was monomorphic (allele 1 in all the 28 samples), and that at chromosome 8 was almost monomorphic (allele 1 in 26 of 28 samples).

### 3.5 MLST among epidemiologically linked samples

We investigated the distribution of MLSTs among samples from confirmed or suspected outbreaks and from epidemiologically unlinked (sporadic) cases.

**Fig. 3:**
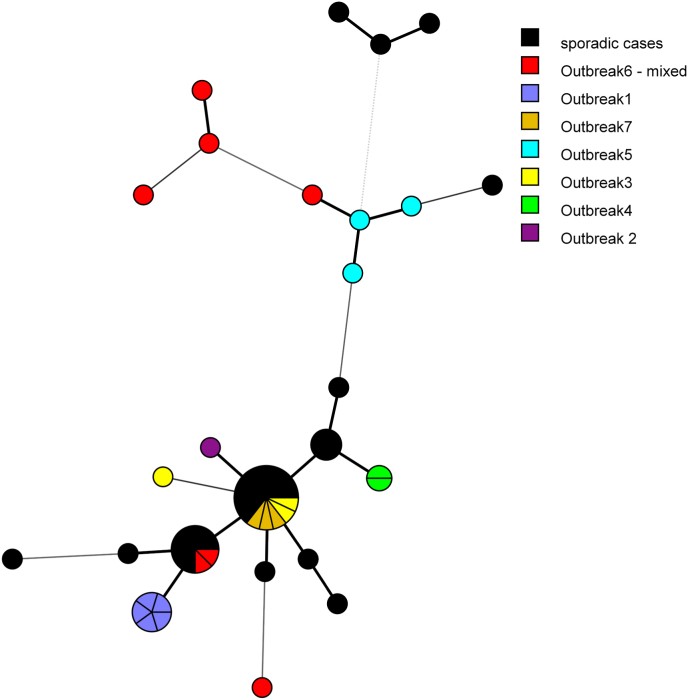
Minimum spanning tree showing the distribution of the MLSTs found in 52 *C. parvum* samples from the UK. Outbreak and sporadic samples are shown in different colors. The size of the circles is proportional to the relative frequency of the corresponding MLST. Branch styles correspond to the relationship between samples: thick solid line for one locus variants; thinner solid line for two or three locus variants; dashed line for four locus variants; and dotted line for five locus variants and above.

Regarding samples from the UK and UK residents, there were 24 samples from five different outbreaks and 28 samples from sporadic cases available for this study. Overall, there were 22 different MLST, of which 15 were singletons (Fig. 3). A unique MLST (14) characterized the five samples from outbreak 1, and another MLST (32) was found in the two samples from outbreak 4 (Supplementary file 1: Table S5). However, the MLST (2) of three samples from outbreak 7 was shared by unrelated samples from the UK and other European countries (Supplementary file 1: Table S5). The allelic differences mentioned previously influenced the MLST; one of the three samples from outbreak 3 had a MLST (92) that differed at two markers from that of the two other samples from the same outbreak (MLST-2, Supplementary file 1: Table S5). Likewise, the three samples from outbreak 5 had three distinct MLSTs (15, 100 and 107, Supplementary file 1: Table S5), although allelic differences were only observed at the marker on chromosome 2.

Finally, samples UKP130 and UKP131, which originated from the inoculum and product, respectively, of an experimental infection of a calf, had different alleles identified by a single SNP difference at the marker on chromosome 4 (Supplementary file 1: Table S5).

Regarding Denmark, 18 samples of human origin and 15 samples from cattle, collected in two regions (Zealand and Funen), were genotyped with the new scheme. These samples were categorized in 20 different MLSTs, of which 12 were singletons (Supplementary file 2: Fig. S5 and Supplementary file 1: Table S5). Among these samples, eight were from a peak in human cases reported at the end of 2021 in Funen, and were previously typed at the *gp60* gene marker to reveal two subtypes (IIdA25G1 and IIdA26G1b). The new scheme identified three MLSTs (25, 49 and 128) in the four samples with the IIdA25G1 subtype and three MLSTs (42, 131 and 132) in the four samples with the IIdA26G1b subtype (Supplementary file 2: Fig. S5 and Supplementary file 1: Table S5). Therefore, the peak in human cases was not linked to a single parasite type. As shown in Supplementary file 2: Fig. S5, the human and cattle MLSTs were mostly unrelated, suggesting a minor role of animals in the transmission of *C. parvum* to humans in the cases studied here.

### 3.6 Comparison with *gp60* typing data

We generated *gp60* sequence data for 269 of the 305 field samples under study, and for all 135 samples with WGS data (Supplementary file 1: Table S5). The Hunter-Gaston discrimination index for the *gp60* gene marker, calculated on a subset of 281 epidemiologically unrelated samples, was 0.85.

Out of the 365 samples that were fully genotyped with the new MLST scheme, *gp60* typing data were available for 338 samples, and the analysis was limited to this subset. There were 282 samples from the IIa subtype family, 55 samples from the IId family, and one sample from the Netherlands from the IIc subtype family. As expected, the IIaA15G2R1 subtype was the most prevalent among IIa samples (found in 107 samples, 38%), followed by subtypes IIaA16G1R1 (found in 54 samples, 19%), IIaA17G1R1 and IIaA16G3R1 (found in 24 and 21 samples, respectively). A minimum spanning tree based on *gp60* families is shown in Supplementary file 2: Fig. S4.

The 107 samples with *gp60* subtype IIaA15G2R1 were classified into 46 different MLSTs (Supplementary file 2: Fig. S6), while the 54 samples with *gp60* subtype IIaA16G1R1 were subdivided into 18 different MLSTs (Supplementary file 2: Fig. S7). Among the 55 samples from the IId family, there were 19 *gp60* subtypes, of which only subtype IIdA21G1 had a relatively high prevalence, being found in 8 samples (of which 7 from humans in Denmark). The 55 IId samples could be subdivided into 38 different MLSTs, of which 28 were singletons (Supplementary file 1: Table S5).

We also observed the presence of different *gp60* subtypes among some samples sharing the same MLST. This was particularly evident for MLST-2 (found in 29 samples) and MLST-3 (found in 22 samples), which were characterized by 11 and 6 different *gp60* subtypes, respectively (Supplementary file 1: Table S5).

### 3.7 Mixed infections

The vast majority of Sanger sequencing trace files did not show evidence of the presence of more than one nucleotide at a given position (a mixed profile, shown as ambiguous nucleotide in the base calling), which can originate when DNA from genetically different parasites present in an infected host is amplified. As PCR amplification generally tends to favor the more abundant population, the chance of detecting the signal originating from the less abundant population(s) is reduced. We observed mixed sequencing profiles in 22 samples (Supplementary file 1: Table S5), of which 18 were from cattle and 4 from humans. In 17 of the 22 cases, mixed profiles were observed at marker CPATCC_0028230 on chromosome 2, and, notably, most (15 of 17) were Danish cattle samples. An example of a mixed sequencing profile is shown in Supplementary file 2: Fig. S8.

### 3.8 Orthologs of *Cryptosporidium hominis* at the selected markers

We identified the orthologous sequences of the eight marker genes in the *Cryptosporidium hominis* genomes available at CryptoDB (https://cryptodb.org/) and generated multiple alignments that included *C. parvum* (Supplementary file 5). The overall homology ranged from 88% (for marker CPATCC_0028230 on chromosome 2) to 99% (for marker CPATCC_0024650 on chromosome 5), and between 93 and 97% for the other markers. The primer sequences designed for *C. parvum* were essentially conserved in *C. hominis* (Supplementary file 5), although mismatches in either the forward or reverse primer sequences were present in several markers (e.g. marker CPATCC_0028230). The *in silico* analysis, therefore, suggested that primers for *C. parvum* could amplify the orthologous sequences from *C. hominis*.

## 4 Discussion

The goal of this study was to select *in silico* and then validate experimentally, novel genetic markers useful to discriminate samples of the zoonotic pathogen *C. parvum*. To this end, we analysed WGS from 135 *C. parvum* samples, which were screened to identify coding regions (500–700 bp in length) with the highest number of SNPs. The overall variability among *C. parvum* samples from the two major zoonotic groups, IIa and IId, is known to be modest, as indicated by several comparative genomic studies (Wang et al., 2022; Corsi et al., 2023; Bellinzona et al., 2024). It was not surprising, therefore, that the selection process identified a relatively small number of polymorphic gene fragments (Supplementary file 1: Table S2), even more so since this study focused solely on European *C. parvum* samples. However, when the discriminatory power of a first selection of 18 polymorphic markers was evaluated *in silico*, 118 distinct MLST were identified among the 135 WGS samples, of which 88% were singletons (Fig. 1 and Supplementary file 1: Table S4). Therefore, a high discrimination is achievable when many markers are used.

As such a large number of markers cannot practically be used in a traditional PCR and sequencing approach, we reduced the scheme to include eight markers, one per chromosome, to ensure they were physically unlinked. This scheme was applied to 305 samples collected across Europe from humans and young ruminants, and additional variability was detected in most of the markers, which led to the description of 154 different MLST among the 365 fully genotyped samples (Supplementary file 1: Table S5). The scheme had an overall typeability of 0.83 and a discriminatory power of 0.98, calculated using the Hunter-Gaston discriminatory index (HGDI) on a subset of 308 epidemiologically unrelated samples. Therefore, the scheme fulfilled the requirements for a typing system in terms of its discriminatory power, which should be > 0.95 (van Belkum et al., 2007; Chalmers and Cacciò, 2016). In comparison, the HGDI for the *gp60* gene marker alone, calculated on 281 epidemiologically unrelated samples, was 0.85.

We generated a minimum spanning network to explore whether the host or geographical origin of the samples influenced the distribution of the MLST. At the European scale, we did not observe a complete clustering of samples by country of origin (Fig. 2) or by host (Supplementary file 2: Fig. S3). This is apparently in contrast with findings from previous studies that, based on analysis of loci containing simple sequence repeats, reported a strong geographical segregation and a correlation between genetic and geographical distance, consistent with a model of isolation by distance (Cacciò et al., 2015). This discrepancy could be due to the slower rate of single-nucleotide substitutions compared to that of repeated sequences, with the latter being more informative to describe the population structure. Future studies comparing the same collection of samples with the two approaches could shed light on this aspect.

One important application of a typing scheme in a public health context is the ability to distinguish clusters of cases from unrelated, sporadic cases occurring at the same time in a given setting. We had the possibility to apply the new MLST scheme to samples from several outbreaks that occurred in the UK. Some of these outbreaks were also analysed in a validation study for a scheme based on seven VNTR loci (Robinson et al., 2022). We found good agreement between the two schemes for outbreak B (outbreak 1 in this study), where five samples had a single MLST (14) and a single MLVA profile, and for outbreak D (outbreak 4 in this study), although only two samples were available for MLST. Unexpectedly, however, we found that the three samples from outbreak A (outbreak 5 in this study) all had different MLST (15, 100, and 107) although they only differ at the marker on chromosome 2 (Supplementary file 1: Table S5). These samples were all characterized as *gp60* subtype IIaA19G1R1 and by a single and unique MLVA profile (Robinson et al., 2022). While this discrepancy is not obvious to explain, it may be useful to recall that whole genome comparison of outbreak samples suggested that replication of the parasite in the host population introduced a small number of SNPs (approximately 10; Nader et al., 2019), and that no sample was found to share an identical genome sequence with another (Bellinzona et al., 2024). Therefore, the small differences in MLST profiles of outbreak samples may genuinely reflect divergence at the genome level, although we cannot exclude sequencing errors. Conversely, while the MLST scheme can be too discriminatory in the public health context, by separating known clusters of epidemiologically linked samples, there are also examples where samples were clustered genetically but had no known epidemiological links. For example, MLST-2 is variable by both *gp60* (11 subtypes) and epidemiologically, as it comprises 29 samples, three from outbreak 7, two of the three cases from outbreak 3, and 24 non-linked samples.

Genotyping of three human samples from Sweden demonstrated the utility of using the MLST approach. Indeed, these cases were thought to represent a small cluster of infection, as also supported by the presence of a single *gp60* subtype (IIdA22G1c). However, we found that the MLST of one of these samples (MLST-86) differed at four markers from that of the other two (MLST-3) and was identical to an unrelated sample with a different *gp60* subtype (IIaA17G1R1c), underlining the risk of building inferences among samples based on a single genetic marker.

The discriminatory power of the new MLST scheme was also compared with that obtained with the commonly used *gp60* gene marker. In Europe, several *gp60* subtypes belonging to the IIa family, such as IIaA15G2R1 and IIaA16G2R1, have a high prevalence in both humans and ruminants, and are also associated with outbreaks (Cacciò and Chalmers, 2016; Chalmers et al., 2019). In the present study, the 107 samples typed as IIaA15G2R1 were subdivided into 46 different MLSTs by the new scheme, highlighting its higher discrimination.

We have shown the potential benefits of an MLST scheme for genotyping *C. parvum* samples in Europe. This study, however, has several limitations. First, a systematic sampling strategy could not be followed, due to the difficulties in performing fieldwork during the COVID-19 pandemic. Consequently, the origin of the samples was biased in terms of geography (with Nordic countries overrepresented) and host species (with more samples from ruminants compared to humans). Additional efforts are also needed to verify the applicability and usefulness of the proposed scheme on samples of non-European origin. Secondly, the proposed typing scheme is admittedly laborious and costly, as it requires eight nested PCR reactions and bidirectional Sanger sequencing per sample. Furthermore, the utility of the MLST scheme is limited by some markers showing little and others too much additional variability when assessed on field samples. Additionally, the difficulty in detecting and discriminating mixed alleles in Sanger sequence traces can result in reduced typeability or different MLSTs between linked cases due to the combination of alleles that have been amplified and sequenced in the sample.

## 5 Conclusions

Our extensive search for coding regions with high genetic variability based on analyses of whole genome sequences of *C. parvum* allowed the identification of many potential markers. The newly developed MLST scheme, which used eight unlinked markers, showed good typeability and ability to discriminate among European samples. However, challenges for scalability and the modest variability of some of the markers can limit its routine applicability. Furthermore, analyses of outbreak samples showed limitations of the scheme, which was not able to fully distinguish these samples from background cases, while this could be achieved using markers containing VNTR. We are currently testing SNP-based and VNTR-based markers in an effort to develop a simplified scheme that includes the most informative, unlinked markers of both types. It is likely that different marker combinations may prove better adapted for use in different public health settings. Ideally, WGS data consent to the most accurate comparison of *Cryptosporidium* samples, allowing description of local population structures and definition of relatedness among outbreak samples *versus* background samples (Huang et al., 2023; Bellinzona et al., 2024). Recent progress in sequencing technologies may reduce the cost of WGS, and the development of a dedicated pipeline (Morris et al., 2025) will contribute to overcoming the current bioinformatics challenges. Alternatively, a large number of SNP-based markers (e.g. the 18 selected markers presented in this study) could be included in an NGS amplicon-sequencing method (e.g. Joeres et al., 2024) to improve the discriminatory power, simplify the overall procedure and provide data on mixed infections.

## Ethical approval

Not applicable.

## CRediT authorship contribution statement

**Karin Troell:** Conceptualization, Investigation, Writing – original draft, Writing – review & editing, Funding acquisition. **Christen Rune Stensvold:** Investigation, Writing – review & editing. **Anna Rosa Sannella:** Investigation, Methodology, Writing – review & editing. **Martha Betson:** Investigation, Writing – original draft, Writing – review & editing. **Emma Östlund:** Conceptualization, Investigation, Methodology, Data curation, Writing – review & editing. **Rachel M. Chalmers:** Conceptualization, Investigation, Writing – review & editing. **Umer Chaudhry:** Investigation, Methodology, Writing – review & editing. **Rebecca Davidson:** Investigation, Methodology, Writing – review & editing. **Lauren Davies:** Investigation, Methodology, Writing – review & editing. **Ralf Ignatius:** Investigation, Methodology, Writing – review & editing. **Anton de Jong:** Investigation, Methodology, Writing – review & editing. **Gregory Karadjian:** Investigation, Methodology, Writing – review & editing. **Karim Adjou:** Investigation, Methodology, Writing – review & editing. **Christian Klotz:** Investigation, Methodology, Writing – review & editing. **Sokratis Ptochos:** Investigation, Methodology, Writing – review & editing. **Guy Robinson:** Investigation, Methodology, Writing – review & editing. **Jeroen Roelfsema:** Investigation, Methodology, Writing – review & editing. **Barbara Soba:** Investigation, Methodology, Writing – review & editing. **Jacek Sroka:** Investigation, Methodology, Writing – review & editing. **Paolo Vatta:** Investigation, Methodology, Data curation, Writing – review & editing. **Jonas Johansson Wensman:** Investigation, Methodology, Writing – review & editing. **Simone M. Cacciò:** Conceptualization, Investigation, Writing – original draft, Writing – review & editing, Funding acquisition.

## Funding

This project has received funding from the European Union’s Horizon 2020 Research and Innovation Programme under grant agreement No. 773830. Sampling and qPCR analysis of the field samples from Swedish cattle was funded by Formas (a Swedish Research Council for Sustainable Development), grant no. 2016–00666.

## Declaration of competing interests

The authors declare that they have no known competing financial interests or personal relationships that could have appeared to influence the work reported in this paper.

## Data Availability

All data generated or analysed during this study are included in this manuscript and its supplementary files. Sequences generated in the present study were deposited in the GenBank public repository database under accession numbers PX056776-PX056783 (marker on chromosome 1), PX056784-PX056792 (marker on chromosome 2), PX060294-PX060300 (marker on chromosome 3), PX060327-PX060345 (marker on chromosome 4), PX060301- PX060305 (marker on chromosome 5), PX060306-PX060312 (marker on chromosome 6), PX060313-PX060317 (marker on chromosome 7) and PX060318-PX060326 (marker on chromosome 8).
