# Supplementary material for: Design, development, and testing of a new multi-locus sequence typing scheme for the zoonotic pathogen Cryptosporidium parvum
Source: Curr Res Parasitol Vector Borne Dis. 2025 Aug 14;8:100308. doi: 10.1016/j.crpvbd.2025.100308 (PMC12446620; doi:10.1016/j.crpvbd.2025.100308)

Supplementary file 2

Supplementary Figure S1. Schematic representation of the analytical workflow used to process *Cryptosporidium parvum* whole genome sequences.

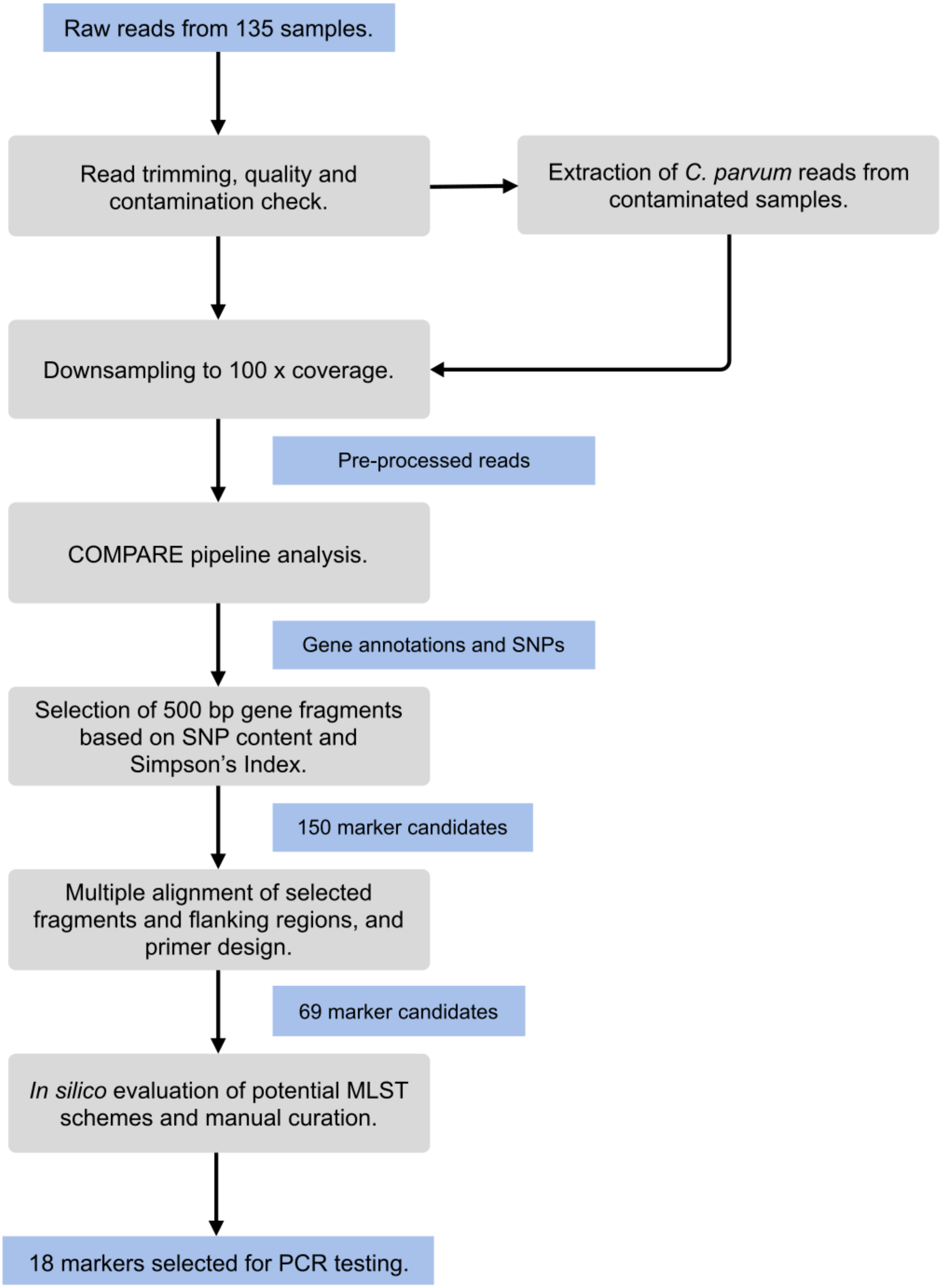

Supplementary Figure S2. Minimum spanning tree of the 73 MLSTs generated using 8 markers and 135 *C. parvum* samples with WGS data. Samples were obtained from 13 European countries, identified with different colors in the figure, as indicated in the legend. The size of the circles is proportional to the relative frequency of the corresponding MLST. Branch styles correspond to the relationship between samples (thick solid line for one locus variants; thinner solid line for two or three locus variants; dashed line for four locus variants; and dotted line for five locus variants and above).

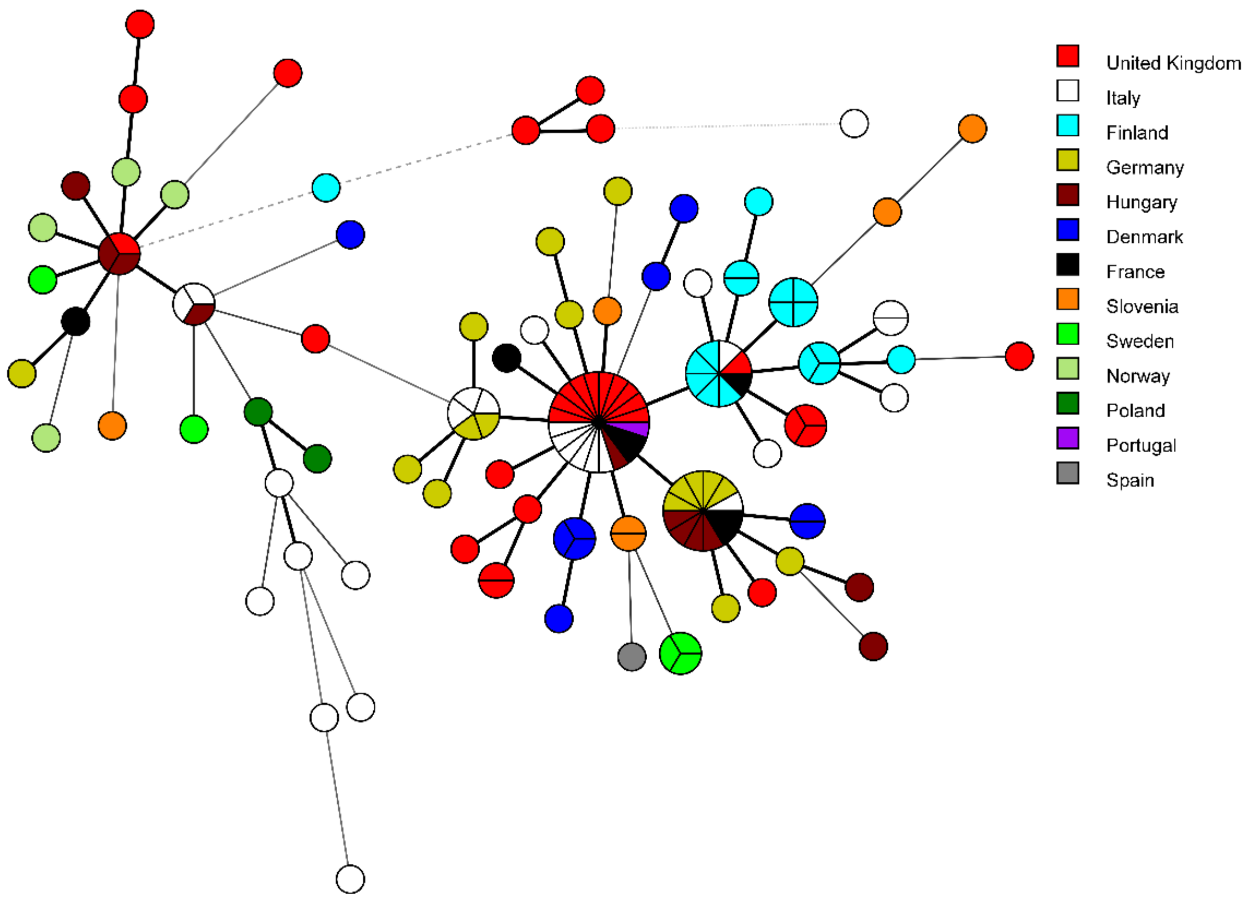

Supplementary Figure S3. Minimum spanning tree showing the distribution of the 154 MLSTs found in 365 *C. parvum* samples from 16 European countries. Samples are labelled by host species (human, cattle, sheep, goat). The size of the circles is proportional to the relative frequency of the corresponding MLST. Branch styles correspond to the relationship between samples (thick solid line for one locus variants; thinner solid line for two or three locus variants; dashed line for four locus variants; and dotted line for five locus variants and above).

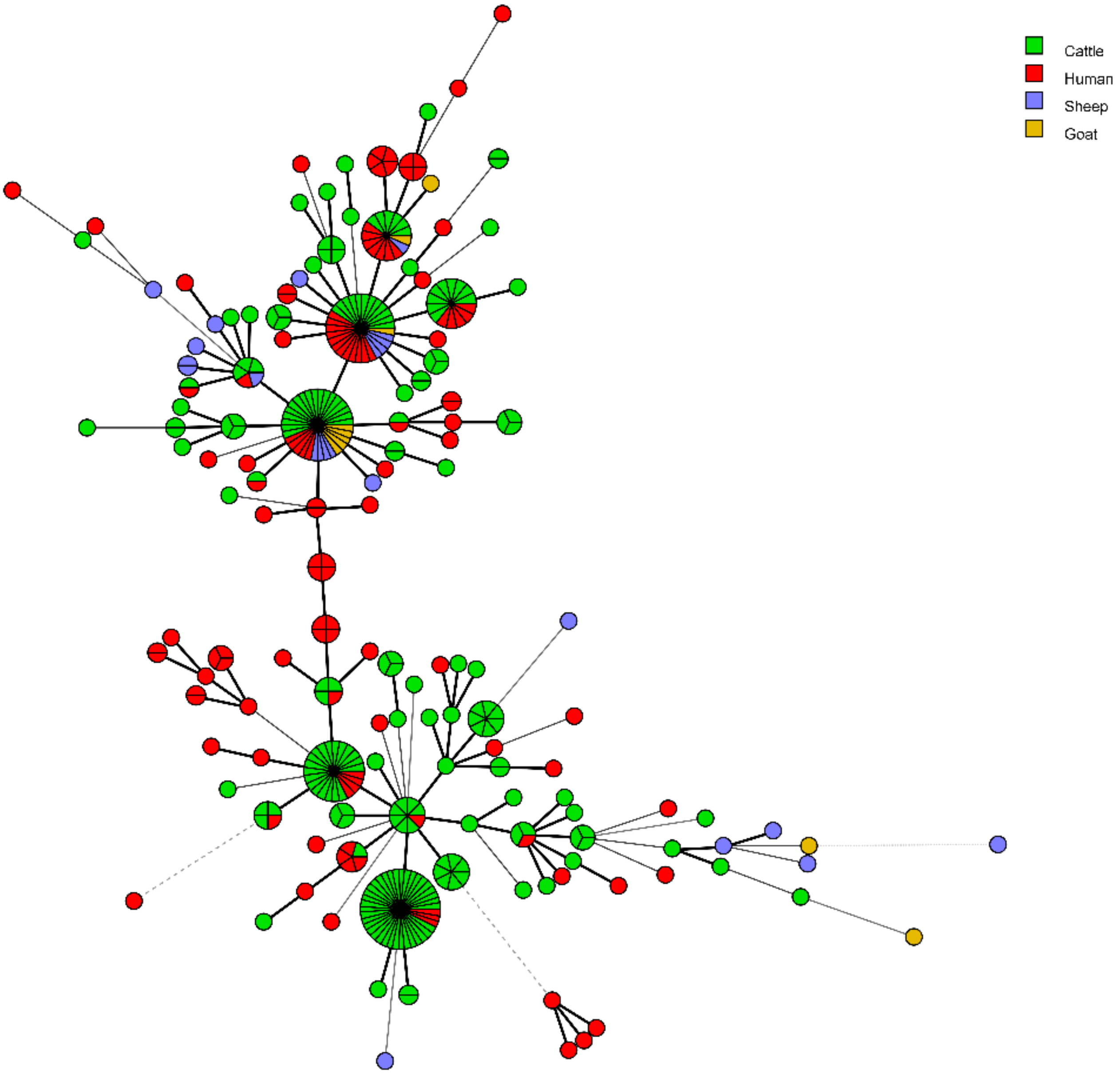

Supplementary Figure S4. Minimum spanning tree showing the distribution of the 154 MLSTs found in 365 *C. parvum* samples from 16 European countries. Samples are labelled by *gp60* subtype family (IIa, IIc, IId). The size of the circles is proportional to the relative frequency of the corresponding MLST. Branch styles correspond to the relationship between samples (thick solid line for one locus variants; thinner solid line for two or three locus variants; dashed line for four locus variants; and dotted line for five locus variants and above).

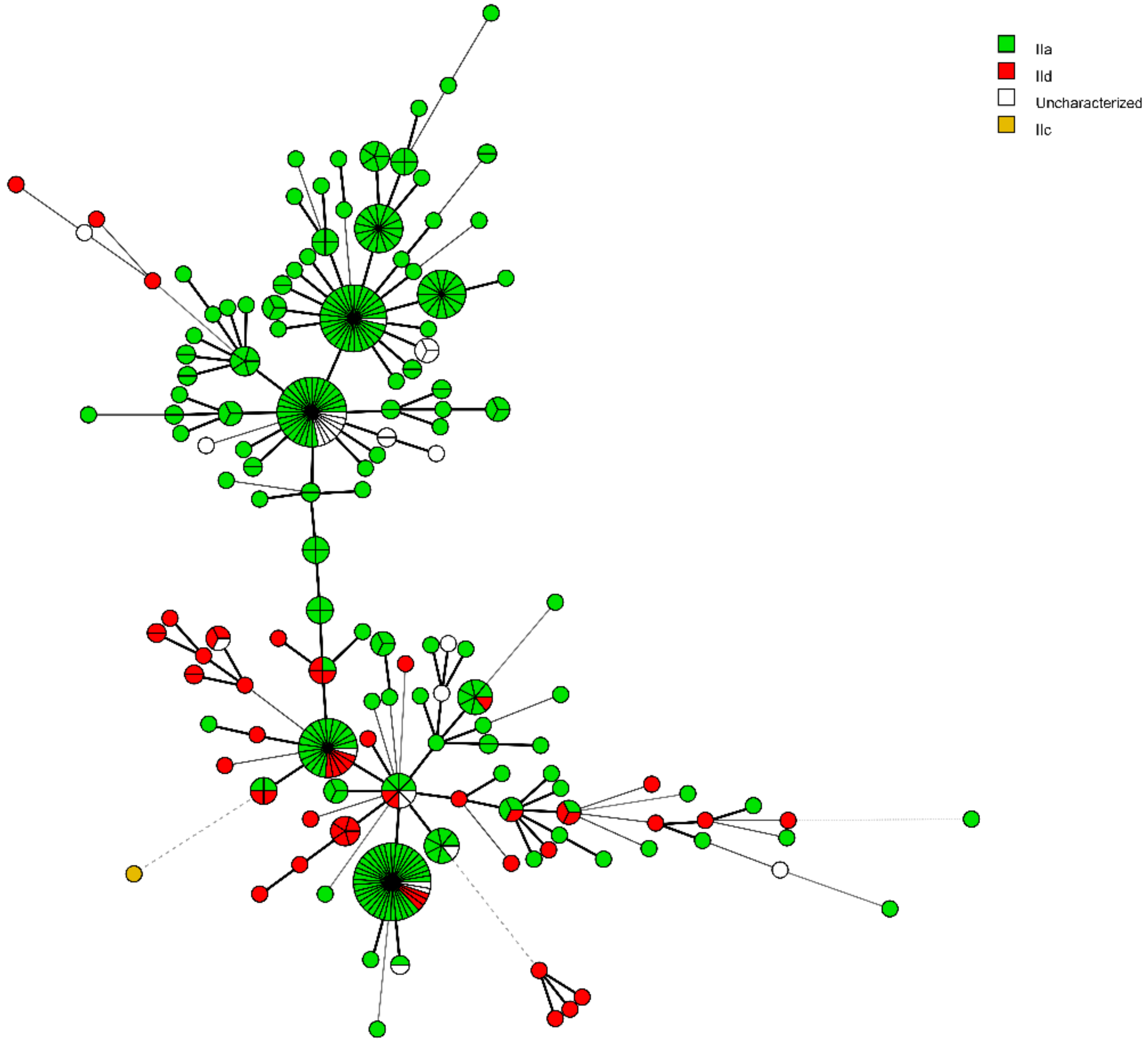

Supplementary Figure S5. Minimum spanning tree showing the distribution of MLSTs in human and cattle samples from Denmark. Samples are labelled by *gp60* subtype family (IIa, IIc, IId). The size of the circles is proportional to the relative frequency of the corresponding MLST. Branch styles correspond to the relationship between samples (thick solid line for one locus variants; thinner solid line for two or three locus variants; dashed line for four locus variants; and dotted line for five locus variants and above).

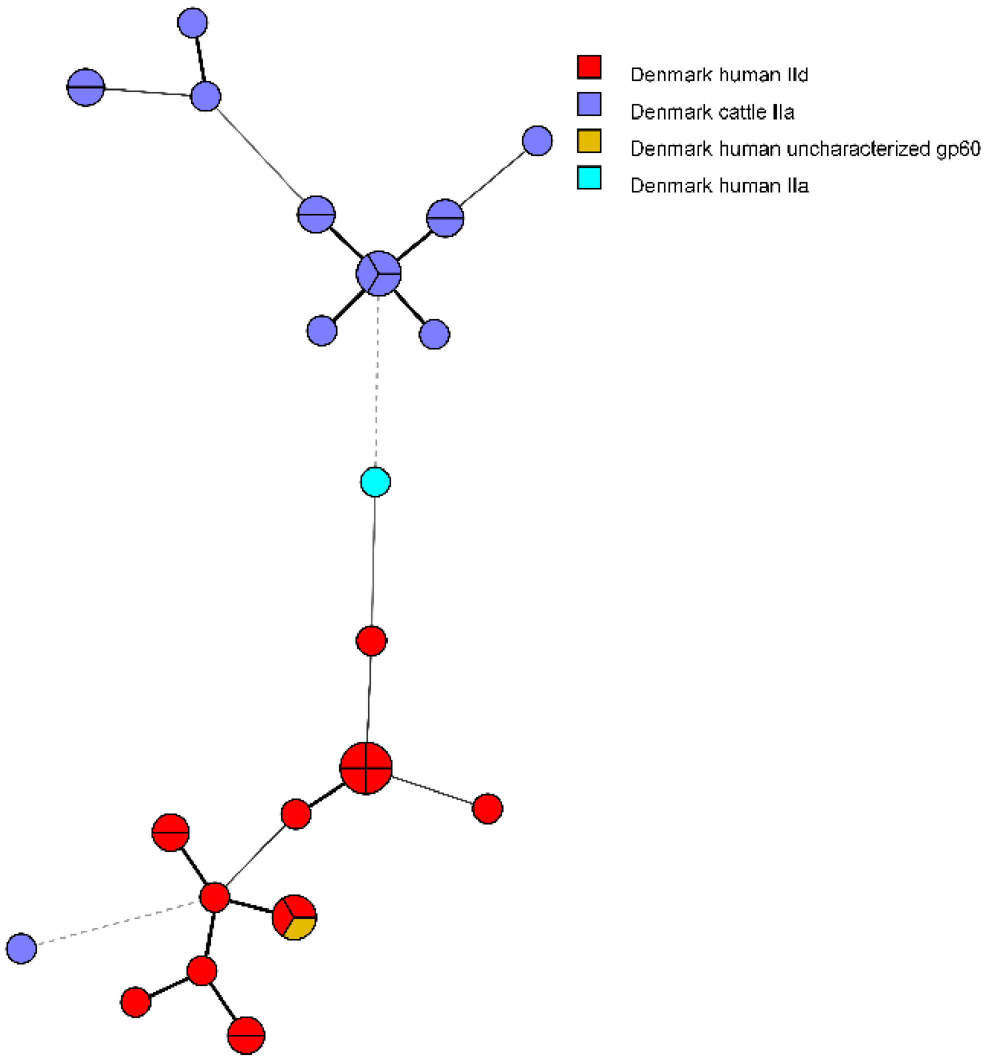

Supplementary Figure S6. Minimum spanning tree showing the distribution of the 45 MLSTs found among 107 *C. parvum* isolates sharing the *gp60* subtype IIaA15G2R1. Colours are used to disinguish the four host species. The size of the circles is proportional to the relative frequency of the corresponding MLST. Branch styles correspond to the relationship between samples (thick solid line for one locus variants; thinner solid line for two or three locus variants; dashed line for four locus variants; and dotted line for five locus variants and above).

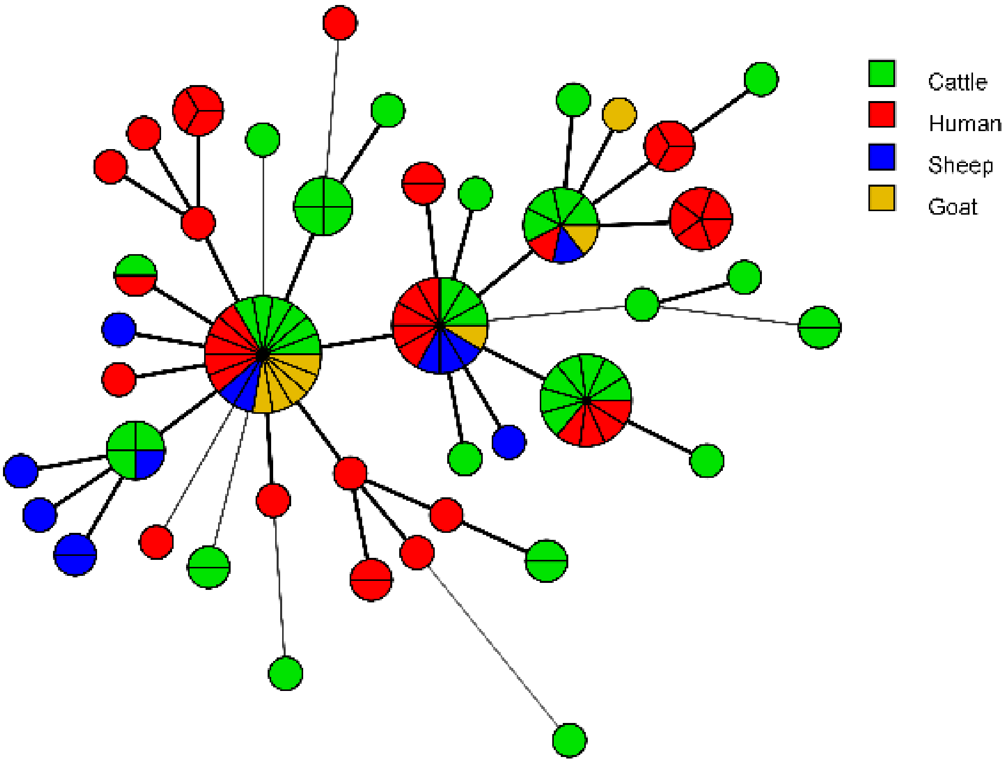

Supplementary Figure S7. Minimum spanning tree showing the distribution of the 15 MLSTs found among 54 *C. parvum* isolates sharing the gp60 subtype IIaA16G1R1. The size of the circles is proportional to the relative frequency of the corresponding MLST. Branch styles correspond to the relationship between samples (thick solid line for one locus variants; thinner solid line for two or three locus variants; dashed line for four locus variants; and dotted line for five locus variants and above).

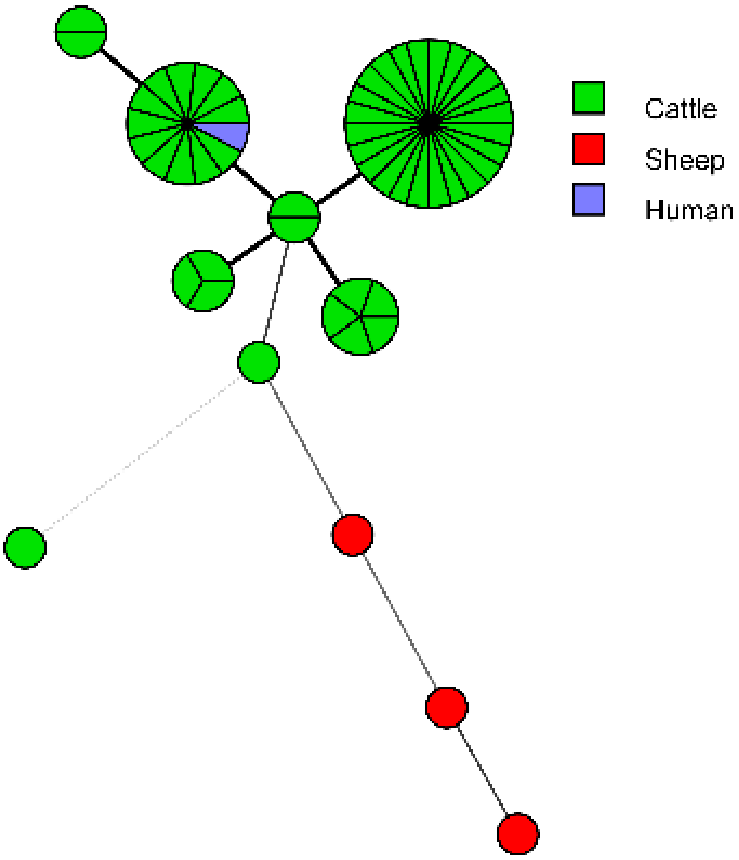

Supplementary Figure S8. Example of a mixed sequencing profile at marker CPATCC-0028230. In the isolate M4, the reverse sequence indicates the presence of a T (with an underlying weak signal for A), whereas the forward sequence indicates the presence of an A at the same position (with an underlying weak signal for T). The position is indicated by an arrow.

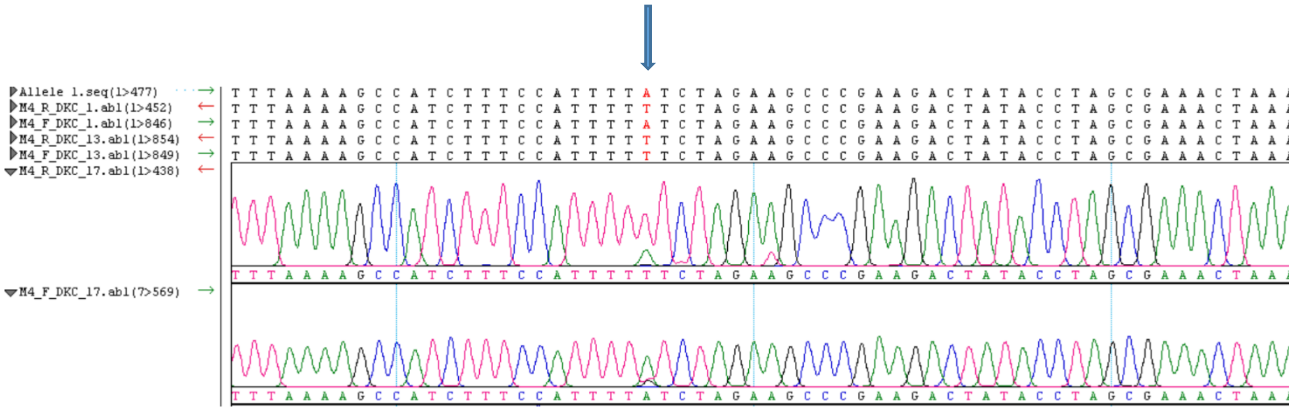

Supplement: Multimedia component 2 [file mmc2.pdf]
