# Supplementary material for: Design, development, and testing of a new multi-locus sequence typing scheme for the zoonotic pathogen Cryptosporidium parvum
Source: Curr Res Parasitol Vector Borne Dis. 2025 Aug 14;8:100308. doi: 10.1016/j.crpvbd.2025.100308 (PMC12446620; doi:10.1016/j.crpvbd.2025.100308)
Supplement: Multimedia component 3 [file mmc3.pdf]

Supplementary file 3

List of the primer sequences used to amplify the eight *C. parvum* gene markers. Primers for nested PCR are highlighted in grey.

Gene ID	Primer sequence (5‘-3‘)	Amplicon size (bp)
Cp0039030-F	ACTGTCAGCATGGAAAATGAATGC	469
Cp0039030-R	CTGGCTCGCTATTAAGGAAGATGA	
Cp0039030-NF	GAAAATGAATGCAATACACAACAA	450
Cp0039030-NR	CGCTATTAAGGAAGATGATACTTT	
Cp0028230-F	TTATCGTACCTGCTAAGCCTCAAT	498
Cp0028230-R	GTCTATTACCGGTGCCATTTTGAT	
Cp0028230-NF	CTGCTAAGCCTCAATATTGTTCAA	478
Cp0028230-NR	TGCCATTTTGATTATGATCCA	
Cp0031960-F	GGTAGAGTTAGGGCTTAAATGAGG	595
Cp0031960-R	TGGATTACTCCAGAACAGGAAAAG	
Cp0031960-NF	TAGAGTTAGGGCTTAAATGAGGAA	587
Cp0031960-NR	ACTCCAGAACAGGAAAAGACATTA	
Cp0021750-F	ATTGGAACTGAAACTTCAACACCC	509
Cp0021750-R	TGTAATCTGGCCGTTTCCAAAAAG	
Cp0021750-NF	ACTGAAACTTCAACACCCCAGGAA	500
Cp0021750-NR	AATCTGGCCGTTTCCAAAAAGCAA	
Cp0024650-F	AACAAGAGCAACTTTCCATCTCAC	536
Cp0024650-R	GTCTGTATTACGGCCTAAAATGGC	
Cp0024650-NF	AGAGCAACTTTCCATCTCACTTTA	526
Cp0024650-NR	ATTACGGCCTAAAATGGCTCCTCCA	
Cp0012400-F	AGATTACCTACACGTCCTTATGGC	512
Cp0012400-R	GATTCCAACCTTTCTGCTTTCTCC	
Cp0012400-NF	CCTACACGTCCTTATGGCCCTGGT	503
Cp0012400-NR	TCCAACCTTTCTGCTTTCTCCGAA	
Cp0o07000-F	TACTTGACTACAACTGGAAGCTCT	568
Cp0007000R	AGCTAGGAAACTCTTTGAGCCAAT	
Cp0007000-NF	GACTACAACTGGAAGCTCTTAGTA	558
Cp00o7000-NR	GGAAACTCTTTGAGCCAATACAAA	
Cp0001010-F	GCCATAACTAGAGCAATCCCAGTA	560
Cp0001010-R	CCAGACCTAAGAGATATTGGAGGC	
Cp0001010-NF	TAACTAGAGCAATCCCAGTAACAA	553
Cp0001010-NR	GACCTAAGAGATATTGGAGGCTTC	
