# Supplementary material for: Design, development, and testing of a new multi-locus sequence typing scheme for the zoonotic pathogen Cryptosporidium parvum
Source: Curr Res Parasitol Vector Borne Dis. 2025 Aug 14;8:100308. doi: 10.1016/j.crpvbd.2025.100308 (PMC12446620; doi:10.1016/j.crpvbd.2025.100308)
Supplement: Multimedia component 4 [file mmc4.pdf]

Supplementary file 4

Multiple alignment of the alleles identified at each of the eight markers. Single-nucleotide polymorphisms and insertions/deletions (for marker CPATCC_021750) are shown in bold and highlighted in yellow.

MARKER CPATCC_039030 (chromosome 1)

Allele1 GAAAATGAATGCAATACACAACAATTGCTTATATCGTTGGGTAAGCAAAAAAAAGAAAAT
Allele2 GAAAATGAATGCAATACACAACAATTGCTTATATCGTTGGGTAAGCAAAAAAAAGAAAAT
Allele3 GAAAATGAATGCAATACACAACAATTGCTTATATCGTTGGGTAAGCAAAAAAAAGAAAAT
Allele4 GAAAATGAATGCAATACACAACAATTGCTTATATCGTTGGGTAAGCAAAAAAAAGAAAAT
Allele5 GAAAATGAATGCAATACACAACAATTGCTTATATCGTTGGGTAAGCAAAAAAAAGAAAAT
Allele6 GAAAATGAATGCAATACACAACAATTGCTTATATCGTTGGGTAAGCAAAAAAAAGAAAAT
Allele7 GAAAATGAATGCAATACACAACAATTGCTTATATCGTTGGGTAAGCAAAAAAAAGAAAAT
Allele8 GAAAATGAATGCAATACACAACAATTGCTTATATCGT**C**GGGTAAGCAAAAAAAAGAAAAT

Allele1 GGAAAAGATGAATTCAAGTTTGTTAGAGGGTTTGTTTTTAACGATTCCGTTGAATCAAGA
Allele2 GGAAAAGATGAATTCAAGTTTGTTAGAGGGTTTGTTTTTAACGATTCCGTTGAATCAAGA
Allele3 GGAAAAGATGAATTCAAGTTTGTTAGAGGGTTTGTTTTTAACGATTCCGTTGAATCAAGA
Allele4 GGAAAAGATGAATTCAAGTTTGTTAGAGGGTTTGTTTTTAACGATTCCGTTGAATCAAGA
Allele5 GGAAAAGATGAATTCAAGTTTGTTAGAGGGTTTGTTTTTAACGATTCCGTTGAATCAAGA
Allele6 GGAAAAGATGAATTCAAGTTTGTTAGAGGGTTTGTTTTTAACGATTCCGTTGAATCAAGA
Allele7 GGAAAAGATGAATTCAAGTTTGTTAGAGGGTTTGTTTT**A**AACGATTCCGTTGAATCAAGA
Allele8 GGAAAAGATGAATTCAAGTTTGTTAGAGGGTTTGTTTTTAACGATTCCGTTGAATCAAGA

Allele1 TCTAGTCGTGGATTTAAGGTGAATTTCAAATACAAGAATTCATCCATTAATATAGCTTCC
Allele2 TCTAGTCGTGGATTTAAGGTGAATTTCAAATACAAGAATTCATCCATTAATATAGCTTC**A**
Allele3 TCTAGTCGTGGATTTAAGGTGAATTTCAAATACAAGAATT**T**ATCCATTAATATAGCTTCC
Allele4 TCTAGTCGTGGATTTAAGGTGAATTTCAAATACAAGAATTCATCCATTAATATAGCTTCC
Allele5 TCTAGTCGTGGATTTAAGGTGAATTTCAAATACAAGAATTCATCCATTAATATAGCTTCC
Allele6 TCTAGTCGTGGATTTAAGGTGAATTTCAAATACAAGAATTCATCCATTAATATAGCTTCC
Allele7 TCTAGTCGTGGATTTAAGGTGAATTTCAAATACAAGAATTCATCCATTAATATAGCTTC**A**
Allele8 TCTAGTCGTGGATTTA**T**GGTGAATTTCAAATACAAGAATTCATCCATTAATATAGCTTC**A**

Allele1 AAATCAAATTCCAACCCAAACTTTATTATTCATACTTGTATTGCAAACATTAAAAATAAA
Allele2 AAATCAAATTCCAACCCAAACTTTATTATTCATACTTGTATTGCAAACATTAAAAATAAA
Allele3 AAATCAAATTCCAACCCAAACTTTATTATTCATACTTGTATTGCAAACATTAAAAATAAA
Allele4 AAATCAAATTCCAACCCAAACTTTATTATTCATACTTGTATTGCAAACATTAAAAATAAA
Allele5 AAATCAAATTCCAACCCAAACTTTATTATTCATACTTGTATTGCAAACATTAAAAATAAA
Allele6 AAATCAAATTCCAACCCAAACTTTATTATTCATACTTGTATTGCAAACATTAAAAATAAA
Allele7 AAATCAAATTCCAACCCAAACTTTATTATTCATACTTGTATTGC**G**AACATTAAAAATAAA
Allele8 AAATCAAATTCCAACCCAAACTTTATTATTCATACTTGTATTGCAAACATTAAAAATAAA

Allele1 CACACGAATGGAATTTGTCAGTGCGGATTCATTAAATTCCCTGATAATAATTCCAACTCT
Allele2 CACACGAATGGAATTTGTCAGTGCGGATTCATTAAATTCCCTGATAATAATTCCAACTCT
Allele3 CACACGAATGGAATTTGTCAGTGCGGATTCATTAAATTCCCTGATAATAATTCCAACTCT
Allele4 CACACGAATGGAATTTGTCAGTGCGGATTCATTAAATTCCCTGATAATAATTCCAACTCT
Allele5 CACACGAATGGAATTTGTCAGTGCGGATTCATTAAATTCCCTGATAATAATTCCAACTCT
Allele6 CACACGAATGGAATTTGTCAGTGCGGATTCATTAAATTCCCTGATAATAATTCCAACTCT
Allele7 CACACGAATGGAATTTGTCAGT**A**CGGATTCATTAAATTCCCTGATAATAATTCCAACTCT
Allele8 CACACGAATGGAATTTGTCAGTGCGGATTCATTAAATTCCCTGATAATAATTCCAACTCT

Allele1 AATACCGCTTTAATCGTGCATTCATCAAGTTCTGATTATTTATGTAATCAACTTTACTTA
Allele2 AATACCGCTTTAATCGTGCATTCATCAAGTTCTGATTATTTATGTAATCAACTTTACTTA
Allele3 AATACCGCTTTAATCGTGCATTCATCAAGTTCTGATTATTTATGTAATCAACTTTACTTA
Allele4 AATACCGCTTTAAT**A**GTGCATTCATCAAGTTCTGATTATTTATGTAATCAACTTTAC**C**TA
Allele5 AATACCGCTTTAATCGTGCATTCATCAAGTTCTGATTATTTATGTAATCAACTTTACTTA
Allele6 AATACCGCTTTAATCGTGCATTCATCAAGTTCTGATTATTTATGTAATCAACTTTACTTA
Allele7 AATACCGCTTTAATCGTGCATTCATCAAGTTCTGATTATTTATGTAATCAACTTTACTTA
Allele8 AATACCGCTTTAATCGTGCATTCATCAAGTTCTGATTATTTATGTAATCAACTTTACTTA

Allele1 AGAAAAAATGAATTCCTTCCTTATTCCGAAATATACCTTAATATAGAACGGAACTCTAAA
Allele2 AGAAAAAATGAATTCCTTCCTTATTCCGAAATATACCTTAATATAGAACGGAACTCTAAA
Allele3 AG**C**AAAAATGAATTCCTTCCTTATTCCGAAATATACCTTAATATAGAACGGAACTCTAAA
Allele4 AG**C**AAAAATGAATTCCTTCCTTATTCCGAAATATACCTTAATATAGAACGGAACTCTAAA
Allele5 AGAAAAAATGAATTCCTTCCTTATTC**T**GAAATATACCTTAATATAGAACGGAACTCTAAA
Allele6 AGAAAAAATGAATTCCTTCCTTATTCCGAAATATACCTTAATATAGAAC**C**GAACTCTAAA
Allele7 AGAAAAAATGAATTCCTTCCTTATTCCGAAATATACCTTAATATAGAACGGAACTCTAAA
Allele8 AGAAAAAATGAATTCCTTCCTTATTCCGAAATATACCTTAATATAGAACGGAACTCTAAA

Allele1 AACAAGCAAAGTATCATCTTCCTTAATAGCG
Allele2 AACAAGCAAAGTATCATCTTCCTTAATAGCG
Allele3 AACAAGCAAAGTATCATCTTCCTTAATAGCG
Allele4 AACAAGCAAAGTATCATCTTCCTTAATAGCG
Allele5 AACAAGCAAAGTATCATCTTCCTTAATAGCG
Allele6 AACAAGCAAAGTATCATCTTCCTTAATAGCG
Allele7 AACAAGCAAAGTATCATCTTCCTTAATAGCG
Allele8 AACAAGCAAAGTATCATCTTCCTTAATAGCG

MARKER CPATCC_028230 (chromosome 2)

Allele1 CTGCTAAGCCTCAATATTGTTCAACAATGAAACTTCCACTGAATGGAGTCGGTAAAAAAA
Allele2 CTGCTAAGCCTCAATATTGTTCAACAATGAAACTTCCACTGAATGGAGTCGGTAAAAAAA
Allele3 CTGCTAAGCCTCAATATTGTTCAACAATGAAACTTCCACTGAATGGAGTCGGTAAAAAAA
Allele4 CTGCTAAGCCTCAATATTGTTCAACAATGAAACTTCCACTGAATGGAGTCGGTAAAAAAA
Allele5 CTGCTAAGCCTCAATATTGTTCAACAATGAAACTTCCACTGAATGGAGTCGGTAAAAAAA
Allele6 CTGCTAAGCCTCAATATTGTTCAACAATGAAACTTCCACTGAATGGAGTCGGTAAAAAAA
Allele7 CTGCTAAGCCTCAATATTGTTCAACAATGAAACTTCCACTGAATGGAGTCGGTAAAAAAA
Allele8 CTGCTAAGCCTCAATATTGTTCAACAATGAAACTTCCACTGAATGGAGTCGGTAAAAAAA
Allele9 CTGCTAAGCCTCAATATTGTTCAACAATGAAACTTCCACTGAATGGAGTCGGTAAAAAAA

Allele1 ATCCTGTTTTACAAAGTGGAAGTAAAGGAACTAATTTAGAAACTTCCTATGAAGATAAAT
Allele2 ATCCTGTTTTACAAAGTGGAAGTAAAGGAACTAATTTAGAAACTTCCTATGAAGATAAAT
Allele3 ATCCTGTTTTACAAAGTGGAAGTAAAGGAACTAATTTAGAAACTTCCTATGAAGATAAAT
Allele4 ATCCTGTTTTACAAAGTGGAAGTAAAGGAACTAATTTAGAAACTTCCTATGAAGATAAAT
Allele5 ATCCTGTTTTACAAAGTGGAAGTAAAGGAACTAATTTAGAAACTTCCTATGAAGATAAAT
Allele6 ATCCTGTTTTACAAAGTGGAAGTAAAGGAACTAATTTAGAAACTTCCTATGAAGATAAAT
Allele7 ATCCTGTTTTACAAAGTGGAAGTAAAGGAACTAATTTAGAAACTTCCTATGAAGATAAAT
Allele8 ATCCTGTTTTACAAAGTGGAAGTAAAGGAACTAATTTAGAAACTTCCTATGAAGATAAAT
Allele9 ATCCTGTTTTACAAAGTGGAAGTAAAGGAACTAATTTAGAAACTTCCTATGAAGATAAAT

Allele1 TTAAAAGCCATCTTTCCATTTTATCTAGAAGCCCGAAGACTATACCTAGCGAAACTAAAC
Allele2 TTAAAAGCCATCTTTCCATTTTATCTAGAAGCCCGAAGACTATACCTAGCGAAACTAAAC
Allele3 TTAAAAGCCATCTTTCCATTTTATCTAGAAGCCCGAAGACTATACCTAGCGAAACTAAAC
Allele4 TTAAAAGCCATCTTTCCATTTTATCTAGAAGCCCGAAGACTATACCTAGCGAAACTAAAC

Allele5 TTAAAAGCCATCTTTCCATTTTATCTAGAAGCCCGAAGACTATACCTAGCGAAACTAAAC
Allele6 TTAAAAGCCATCTTTCCATTTTATCTAGAAGCCCGAAGACTATACCTAGCGAAACTAAAC
Allele7 TTAAAAGCCATCTTTCCATTTTATCTAGAAGCCCGAAGACTATACCTAGCGAAACTAAAC
Allele8 TTAAAAGCCATCTTTCCATTTTATCTAGAAGCCCGAAGACTATACCTAGCGAAACTAAAC
Allele9 TTAAAAGCCATCTTTCCATTTT**T**TCTAGAAGCCCGAAGACTATACCTAGCGAAACTAAAC

Allele1 AAAATAAACTTAGATGGAAAGTAGAAAATACAGGCGCAGAGAGATCAAGTTCGGATAACA
Allele2 AAAATAAACTTAGATGGAAAGTAGAAAATACAGGCGCAGAGAGATCAAGTTCGGATAACA
Allele3 AAAATAAACTTAGATGGAAAGTAGAAAATACAGGC**A**CAGAGAGATCAAGTTCGGATAACA
Allele4 AAAATAAACTTAGATGGAAAGTAGAAAATACAGGC**A**CAGAGAGATCAAGTTCGGATAACA
Allele5 AAAATAAACTTAGATGGAAAGTAGAAAATACAGGC**A**CAGAGAGATCAAGTTCGGATAACA
Allele6 AAAATAAACTTAGATGGAAAGTAGAAAATACAGGCGCAGAGAGATCAAGTTCGGATAACA
Allele7 AAAATAAACTTAGATGGAAAGTAGAAAATACAGGC**A**CAGAGAGATCAAGTTCGGATAACA
Allele8 AAAATAAACTTAGATGGAAAGTAGAAAATACAGGCGCAGAGAGATCAAGTTCGGATAACA
Allele9 AAAATAAACTTAGATGGAAAGTAGAAAATACAGGCGCAGAGAGATCAAGTTCGGATAACA

Allele1 GAAAAAACGGTCCCAGATCTGGAATGCTTGGAGGGCCTATTCAACATAGTGCTGAAGGAA
Allele2 GAAAAAACGGTCCCAGATCTGGAATGCTTGGAGGGCCTATTCAACATAGTGCTGAAGGAA
Allele3 GAAAAAACGGTCCCAGATCTGGAATGCTTGGAGGGCCTATTCAACATAGTGCTGAAGGAA
Allele4 GAAAAAACGGTCCCAGATCTGGAATGCTTGGAGGGCCTATTCAACATAGTGCTGAAGGAA
Allele5 GAAAAAACGGTCCCAGATCTGGAATGCTTGGAGGGCCTATTCAACATAGTGCTGAAGGAA
Allele6 GAAAAAACGGTCCCAGATCTGGAATGCTTGGAGGGCCTATTCAACATAGTGCTGAAGGAA
Allele7 GAAAAAACGGTCCCAGATCTGGAATGCTTGGAGGGCCTATTCAACATAGTGCTGAAGGAA
Allele8 GAAAAAACGGTCCCAGATCTGGAATGCTTGGAGGGCCTATTCAACATAGTGCTGAAGGAA
Allele9 GAAAAAACGGTCCCAGATCTGGAATGCTTGGAGGGCCTATTCAACATAGTGCTGAAGGAA

Allele1 TGGATACTTACCATGCAACTTATGTTGAGGTTGATGATTCCTCTGATGAGGAGGACACAA
Allele2 TGGATACTTACCATGCAACTTATGTTGAGGTTGATGATTCCTCTGATGAGGAGG**G**CA**A**AA
Allele3 TGGATACTTACCATGCAACTTATGTTGAGGTTGATGATTCCTCTGATGAGGAGGACACAA
Allele4 TGGATACTTACCATGCAACTTATGTTGAGGTTGATGATTCCTCTGATGAGGAGGACACAA
Allele5 TGGATACTTACCATGCAACTTATGTTGAGGTTGATGATTCCTCTGATGAGGAGGACACAA
Allele6 TGGATACTTACCATGCAACTTATGTTGAGGTTGATGATTCCTCTGATGAGGAGGACACAA
Allele7 TGGATACTTACCATGCAACTTATGTTGAGGTTGATGATTCCTCTGATGAGGAGG**G**CA**A**AA
Allele8 TGGATACTTACCATGCAACTTATGTTGA**C**GTTGATGATTCCTCTGATGAGGAGGACACAA
Allele9 TGGATACTTACCATGCAACTTATGTTGAGGTTGATGATTCCTCTGATGAGGAGGACACAA

Allele1 TTAATCCAGTTGGTCCTCCGGACGCAGACCCAAGCGTCGCACAAATTTTGAAGCTATTAG
Allele2 TTAATCCAGTTGGTCCTCCGGACG**A**AGA**A**CCAAGCGTCGCACAAATTTTGAAGCTATTAG
Allele3 TTAATCCAGTTGGTCCTCCGGACGCAGACCCAAGCGTCGCACAAATTTTGAAGCTATTAG
Allele4 TTAATC**T**AGTTGGTCCTCCGGA**T**GCAGACCCAAGCGTCGCACAAATTTTGAAGCTATTAG
Allele5 TTAATCCAGTTGGTCCTCCGGA**T**GCAGACCCAAGCGTCGCACAAATTTTGAAGCTATTAG
Allele6 TTAATCCAGTTGGTCCTCCGGACGCAGA**A**CCAAGCGTCGCACAAATTTTGAAGCTATTAG
Allele7 TTAATC**T**AGTTGGTCCTCCGGACGCAGACCCAAGCGTCGCACAAATTTTGAAGCTATTAG
Allele8 TTAATCCAGTTGGTCCTCCGGACGCAGACCCAAGCGTCGCACAAATTTTGAAGCTATTAG
Allele9 TTAATCCAGTTGGTCCTCCGGACGCAGACCCAAGCGTCGCACAAATTTTGAAGCTATTAG

Allele1 AGCTTGATATAGATCTGAAAACAAATGAAGAAGCTGTGGATCATAATCAAAATGGCA
Allele2 AGCTTGATATAGATCTGAAAACAAATGAAGAAGCTGTGGATCATAATCAAAATGGCA
Allele3 AGCTTGATATAGATCTGAAAACAAATGAAGAAGCTGTGGATCATAATCAAAATGGCA
Allele4 AGCTTGATATAGATCTGAAAACAAATGAAGAAGCTGTGGATCATAATCAAAATGGCA
Allele5 AGCTTGATATAGATCTGAAAACAAATGAAGAAGCTGTGGATCATAATCAAAATGGCA
Allele6 AGCTTGATATAGATCTGAAAACAAATGAAGAAGCTGTGGATCATAATCAAAATGGCA
Allele7 AGCTTGATATAGATCTGAAAACAAATGAAGAAGCTGTGGATCATAATCAAAATGGCA
Allele8 AGCTTGATATAGATCTGAAAACAAATGAAGAAGCTGTGGATCATAATCAAAATGGCA
Allele9 AGCTTGATATAGATCTGAAAACAAATGAAGAAGCTGTGGATCATAATCAAAATGGCA

MARKER CPATCC_031960 (chromosome 3)

Allele1 TAGAGTTAGGGCTTAAATGAGGAAAATCTTTAATATTAATAACGTTATGGTTTTCTAGAT
Allele2 TAGAGTTAGGGCTTAAATGAGGAAAATCTTTAATATTAATAACGTTATGGTTTTCTAGAT
Allele3 TAGAGTTAGGGCTTAAATGAGGAAAATCTTTAATATTAATAACGTTATGGTTTTCTAGAT
Allele4 TAGAGTTAGGGCTTAAATGAGGAAAATCTTTAATATTAATAACGTTATGGTTTTCTAGAT
Allele5 TAGAGTTAGGGCTTAAATGAGGAAAATCTTTAATATTAAT**G**ACGTTATGGTTTTCTAGAT
Allele6 TAGAGTTAGGGCTTAAATGAGGAAAATCTTTAATATTAATAACGTTATGGTTTTCTAGAT
Allele7 TAGAGTTAGGGCTTAAATGAGGAAAATCTTTAATATTAATAACGTTATGGTTTTCTAGAT
Allele8 TAGAGTTAGGGCTTAAATGAGGAAAATCTTTAATATTAATAACGTTATGGTTTTCTAGAT

Allele1 CGTCTTGGTGCCTGAAATTCGAGATTTGAGTAGAATTATAAATATTAGAAATAGAACTGG
Allele2 CGTCTTGGTGCCTGAAATTCGAGATTTGAGTAGAATTATAAATATTAGAAATAGAA**T**TGG
Allele3 CGTC**C**TGGTGCCTGAAATTCGAGATTTGAGTAGAATTATAAATATTAGAAATAGAA**T**TGG
Allele4 CGTCTTGGTGCCTGAAATTCGAGATTTGAGTAGAATTATAAATATTAGAAATAGAAC**A**GG
Allele5 CGTCTTGGTGCCTGAAATTCGAGATTTGAGTAGAATTATAAATATTAGAAATAGAA**T**TGG
Allele6 CGTCTTGGTGCCTGAAATTCGAGATTTGAGTAGAATTATAAATATTAGAAATAGAA**T**TGG
Allele7 CGTC**C**TGGTGCCTGAAATTCGAGATTTGAGTAGAATTATAAATATTAGAAATAGAA**T**TGG
Allele8 CGTCTTGGTGCCTGAAATTCGAGATTTGAGTAGAA**C**TATAAATATTAGAAATAGAACTGG

Allele1 TAAATAGGTAAAGCTTACCTGGATGAAATTCCATGTAGAAGTTGAGTATAGCATAGATCT
Allele2 TAAATAGGTAAAGCTTACCTGGATGAAATTCCATGTAGAAGTTGAGTATAGCATAGATCT
Allele3 TAAATAGGTAAAGCTTACCTGGATGAAATTCCATGTAGAAGTTGAGTATAGCATAGATCT
Allele4 TAAATAGGTAAAGCTTACCTGGATGAAATTCCATGTAGAAGTTGAGTATAGCATAGATCT
Allele5 TAAATAGGTAAAGCTTACCTGGATGAAATTCCATGTAGAAGTTGAGTATAGCATAGATCT
Allele6 TAAATAGGTAAAGCTTACCTGGATGAAATTCCATGTAGAAGTTGAGTATAGCATAGATCT
Allele7 TAAATAGGTAAAGCTTACCTGGATGAAATTCCATGTAGAAGTTGAGTATAGCATAGATCT
Allele8 TAAATAGGTAAAGCTTACCTGGATGAAATTCCATGTAGAAGTTGAGTATAGCATAGATCT

Allele1 TGAAAGACTAGTAGGCTGAGTTTGGGGACAATAAGTGAGAATAGAGGGAATTTGTTTTCT
Allele2 TGAAAGACTAGTAGGCTGAGTTTGGGGACAATAAGTGAGAATAGAGGGAATTTGTTTTCT
Allele3 TGAAAGACTAGTAGGCTGAGTTTGGGGACAATAAGTGAGAATAGAGGGAATTTGTTTTCT
Allele4 TGAAAGACTAGTAGGCTGAGTTTGGGGACAATAAGTGAGAATAGAGGGAATTTGTTTTCT
Allele5 TGAAAGACTAGTAGGCTGAGTTTGGGGACAATAAGTGAGAATAGAGGGAATTTGTTTTCT
Allele6 TGAAAGACTAGTAGGCTGAGTTTGGGGACAATAAGTGAGAATAGAGGGAATTTGTTTTCT
Allele7 TGAAAGACTAGTAGGCTGAGTTTGGGGACAATAAGTGAGAATAGAGGGAATTTGTTTTCT
Allele8 TGAAAGACT**G**GTAGGCTGAGTTTGGGGACAATAAGTGAGAATAGAGGGAATTTGTTTTCT

Allele1 TGAGTATCAAGAGAGGGATTCCAAAAATCGAATAAAGTGTCAGATTCTAATGAGATGATT
Allele2 TGAGTATCAAGAGAGGGATTCCAAAAATCGAATAAAGTGTCAGATTCTAATGAGATGATT
Allele3 TGAGTATCAAGAGAGGGATTCCAAAAATCGAATAAAGTGTCAGATTCTAATGAGATGATT
Allele4 TGAGTATCAAGAGAGGGATTCCAAAAATCGAATAAAGTGTCAGATTCTAATGAGATGATT
Allele5 TGAGTATCAAGAGAGGGATTCCAAAAATCGAATAAAGTGTCAGATTCTAATGAGATGATT
Allele6 TGAGTATCAAGAGAGGGATTCCAAAAATCGAATAAAGTGTCAGATTCTAATGAGATGATT
Allele7 TGAGTATCAAGAGAGGGATTCCAAAAATCGAATAAAGTGTCAGATTCTAATGAGATGATT
Allele8 TGAGTATCAAGAGAGGGATTCCAAAAATCGAATAAAGTGTCAGATTCTAATGAGATGATT

Allele1 GCGACTCTTGGGTAATAAAGAGGGCCATCCTTGTGAGGAAGAATCCCTTTGTATTGTTCA
Allele2 GCGACTCTTGGGTAATAAAGAGGGCCATCCTTGTGAGGAAGAATCCCTTTGTATTGTTCA
Allele3 GCGACTCTTGGGTAATAAAGAGGGCCATCCTTGTGAGGAAGAATCCCTTTGTATTGTTCA
Allele4 GCGACTCTTGGGTAATAAAGAGGGCCATCCTTGTGAGGAAGAATCCCTTTGTATTGTTCA
Allele5 GCGACTCTTGGGTAATAAAGAGGGCCATCCTTGTGAGGAAGAATCCCTTTGTATTGTTCA
Allele6 GCGACTCTTGGGTAATAAAGAGGGCCATCCTTGTGAGGAA**A**AATCCCTTTGTATTGTTCA
Allele7 GCGACTCTTGGGTAATAAAGAGGGCCATCCTTGTGAGGAAGAATCCCTTTGTATTGTTCA
Allele8 GCGACTCTTGGGTAATAAAGAGGGCCATCCTTGTGAGGAAGAATCCCTTTGTATTGTTCA

Allele1 TATTGATTGATTAGTACATGGTTTGGAGTTTCTTCTTTTGAAAATATATTGTAATCTACT
Allele2 TATTGATTGATTAGTACATGGTTTGGAGTTTCTTCTTTTGAAAATATATTGTAATCTACT
Allele3 TATTGATTGATTAGTACATGGTTTGGAGTTTCTTCTTTTGAAAATATATTGTAATCTACT
Allele4 TATTGATTGATTAGTACATGGTTTGGAGTTTCTTCTTTTGAAAATATATTGTAATCTACT
Allele5 TATTGATTGATTAGTACATGGTTTGGAGTTTCTTCTTTTGAAAATATATTGTAATCTACT
Allele6 TATTGATTGATTAGTACATGGTTTGGAGTTTCTTCTTTTGAAAATATATTGTAATCTACT
Allele7 TATTGATTGATTAGTACATGGTTTGGAGTTTCTTCTTTTGAAAATATATTGTAATCTACT
Allele8 TATTGATTGATTAGTACATGGTTTGGAGTTTCTTCTTTTGAAAATATATTGTAA**C**CTACT

Allele1 AAAGATTGAGAAATAGATTCTAACCATTCAGGTAAATCTTTCTGATTAACAATTCCTGAT
Allele2 AAAGATTGAGAAATAGATTCTAACCATTCAGGTAAATCTTTCTGATTAACAATTCCTGAT
Allele3 AAAGATTGAGAAATAGATTCTAACCATTCAGGTAAATCTTTCTGATTAACAATTCCTGAT
Allele4 AAAGATTGAGAAATAGATTCTAACCATTCAGGTAAATCTTTCTGATTAACAATTCCTGAT
Allele5 AAAGATTGAGAAATAGATTCTAACCATTCAGGTAAATCTTTCTGATTAACAATTCCTGAT
Allele6 AAAGATTGAGAAATAGATTCTAACCATTCAGGTAAATCTTTCTGATTAACAATTCCTGAT
Allele7 AAAGATTGAGAAATAGATTCTAACCATTCAGGTAAATCTTTCTGATTAACAATTCCTGAT
Allele8 AAAGATTGAGAAATAGATTCTAACCATTCAGGTAAATCTTTCTGATTAACAATTCCTGAT

Allele1 TCAGAGACTGTTCCTCCCCATACTTGAGTTTGTCTTCCGTTCAATTTTACATTTAGAAAT
Allele2 TCAGAGACTGTTCCTCCCCATACTTGAGTTTGTCTTCCGTTCAATTTTACATTTAGAAAT
Allele3 TCAGAGACTGTTCCTCCCCATACTTGAGTTTGTCTTCCGTTCAATTTTACATTTAGAAAT
Allele4 TCAGAGACTGTTCCTCCCCATACTTGAGTTTGTCTTCCGTTCAATTTTACATTTAGAAAT
Allele5 TCAGAGACTGTTCCTCCCCATACTTGAGTTTGTCTTCCGTTCAATTTTACATTTAGAAAT
Allele6 TCAGAGACTGTTCCTCCCCATACTTGAGTTTGTCTTCCGTTCAATTTTACATTTAGAAAT
Allele7 TCAGAGACTGTTCCTCCCCATACTTGAGTTTGTCTTCCGTTCAATTTTACATTTAGAAAT
Allele8 TCAGAGACTGTTCCTCCCCATACTTGAGTTTGTCTTCCGTTCAATTTTACATTTAGAAAT

Allele1 GAGGATCTTGAGATATTCTCTAGTAATGTCTTTTCCTGTTCTGGAGT
Allele2 GAGGATCTTGAGATATTCTCTAGTAATGTCTTTTCCTGTTCTGGAGT
Allele3 GAGGATCTTGAGATATT**A**TCTAGTAATGTCTTTTCCTGTTCTGGAGT
Allele4 GAGGATCTTGAGATATTCTCTAGTAATGTCTTTTCCTGTTCTGGAGT
Allele5 GAGGATCTTGAGATATTCTCTAGTAATGTCTTTTCCTGTTCTGGAGT
Allele6 GAGGATCTTGAGATATTCTCTAGTAATGTCTTTTCCTGTTCTGGAGT
Allele7 GAGGATCTTGAGATATTCTCTAGTAATGTCTTTTCCTGTTCTGGAGT
Allele8 GAGGATCTTGAGATATTCTCTAGTAATGTCTTTTCCTGTTCTGGAGT

MARKER CPATCC_021750 (chromosome 4)

Allele1 ACTGAAACTTCAACACCCCAGGAAGGACATATATCAAGATCAAAAACAAGATCAAAATCA
Allele2 ACTGAAACTTCAACACCCCAGGAAGGACATATATCAAGATCAAAAACAAGATCAAAATCA
Allele3 ACTGAAACTTCAACACCCCAGGAAGGACATATATCAAGATCAAAAACAAGATCAAAATCA
Allele4 ACTGAAACTTCAACACCCCAGGAAGGACATATATCAAGATCAAAAACAAGATCAAAATCA
Allele5 ACTGAAACTTCAACACCCCAGGAAGGACATATATCAAGATCAAAAACAAGATCAAAATCA
Allele6 ACTGAAACTTCAACACCCCAGGAAGGACATATATCAAGATCAAAAACAAGATCAAAATCA
Allele7 ACTGAAACTTCAACACCCCAGGAAGGACATATATCAAGATCAAAAACAAGATCAAAATCA
Allele8 ACTGAAACTTCAACACCCCAGGAAGGACATATATCAAGATCAAAAACAAGATCAAAATCA
Allele9 ACTGAAACTTCAACACCCCAGGAAGGACATATATCAAGATCAAAAACAAGATCAAAATCA
Allele10 ACTGAAACTTCAACACCCCAGGAAGGACATATATCAAGATCAAAAACAAGATCAAAATCA
Allele11 ACTGAAACTTCAACACCCCAGGAAGGACATATATCAAGATCAAAAACAAGATCAAAATCA
Allele12 ACTGAAACTTCAACACCCCAGGAAGGACATATATCAAGATCAAAAACAAGATCAAAATCA
Allele13 ACTGAAACTTCAACACCCCAGGAAGGACATATATCAAGATCAAAAACAAGATCAAAATCA
Allele14 ACTGAAACTTCAACACCCCAGGAAGGACATATATCAAGATCAAAAACAAGATCAAAATCA
Allele15 ACTGAAACTTCAACACCCCAGGAAGGACATATATCAAGATCAAAAACAAGATCAAAATCA
Allele16 ACTGAAACTTCAACACCCCAGGAAGGACATATATCAAGATCAAAAACAAGATCAAAATCA
Allele17 ACTGAAACTTCAACACCCCAGGAAGGACATATATCAAGATCAAAAACAAGATCAAAATCA
Allele18 ACTGAAACTTCAACACCCCAGGAAGGACATATATCAAGATCAAAAACAAGATCAAAATCA
Allele19 ACTGAAACTTCAACACCCCAGGAAGGACATATATCAAGATCAAAAACAAGATCAAAATCA

Allele1 AAATCAAGATCAAAATCAAGATCAAAATCAAGATCAAAATCAAAATCAAGAGCAAGATCA
Allele2 AAATCAAGATCAAAATCAAGATCAAAATCAAGATCAAAATCAAAATCAAGAGCAAGATCA
Allele3 AAATCAA**A**ATCAAAATCAAGATCAAAATCAA**A**ATCAAAATCAAAATCAAGAGCAAGATCA
Allele4 AAATCAAGATCAAAATCAAGATCAAAATCAAGATCAAAATCAAAATCAAGAGCAAGATCA
Allele5 AAATCAAGATCAAAATCAA**A**ATCAAAATCAAGATCAAAATCAAAATCAAGAGCAAGATCA
Allele6 AAATCA**G**GATCAAAATCAAGATCAAAATCAAGATCAAAATCAAAATCAAGAGCAAGATCA
Allele7 AAATCA**G**GATCAAAATCAAGATCAAAATCAAGATCAAAATCAAAATCAAGAGCAAGATCA
Allele8 AAATCA**G**GATCAAAATCAAGATCAAAATCAAGATCAAAATCAAAATCAAGAGCAAGATCA
Allele9 AAATCAAGATCAAAATCAAGATCAAAATCAAGATCAAAATCAAAATCAAGAGCAAGATCA
Allele10 AAATCA**G**GATCAAAATCAAGATCAAAATCAAGATCAAAATCAAAATCAAGAGCAAGATCA
Allele11 AAATCAAGATCAAAATCAA**A**ATCAAAATCAAGATCAAAATCAAAATCAAGAGCAAGATCA
Allele12 AAATCAAGATCAAAATCAAGATCAAAATCAA**A**ATCAAAATCAAAATCAAGAGCAAGATCA
Allele13 AAATCAAGATCAAAATCAAGATCAAAATCAAGATCAAAATCAAAATCAAGAGCAAGATCA
Allele14 AAA------------------------------TCAAAATCAAAATCAAGAGCAAGATCA
Allele15 AAATCAAGATCAAAATCAAGATCAAAATCAAGATCAAAATCAAAATCAAGAGCAAGATCA
Allele16 AAATCAAGATCAAAATCAAGATCAAAATCAAGATCAAAATCAAAATCAAGAGCAAGATCA
Allele17 AAATCAA**A**ATCAAAATCAAGATCAAAATCAA**A**ATCAAAATCAAAATCAAGAGCAAGATCA
Allele18 AAATCAAGATCAAAATCAAGATCAAAATCAAGATCAAAATCAAAATCAAGAGCAAGATCA
Allele19 AAATCAA**A**ATCAAAATCAAGATCAAAATCAA**A**ATCAAAATCAAAATCAAGAGCAAGATCA

Allele1 AAATCAAGAACAAGAGCAAGATCAAAATCAAGATCAAGATCAAGA------ACAAGATCA
Allele2 AAATCAAGAACAAGAGCAAGATCAAAATCAAGATCAAGATCAAGA------ACAAGATCA
Allele3 AAATCAAGAACAAGAGCAAGATCAAAATCAAGATCAAGATCAAGA------ACAAGATCA
Allele4 AAATCAAGAACAAGAGCAAGATCAAAATCAAGATCAAGATCAAGA------ACAAGATCA
Allele5 AAATCAAGAACAAGAGCAAGATCAAAATCAAGATCAAGATCAAGA------ACAAGATCA
Allele6 AAATCAAGAACAAGAGCAAGATCAAAATCAAGATCAAGATCAAGA------ACAAGATCA
Allele7 AAATCAAGAACAAGAGCAAGATCAAAATCAAGATCAAGATCAAGA------ACAAGATCA
Allele8 AAATCAAGAACAAGAGCAAGATCAAAATCAAGATCAAGATCAAGA------ACAAGATCA
Allele9 AAATCAAGAACAAGAGCAAGATCAAAATCAAGATCAAGATCAAGA------ACAAGATCA
Allele10 AAATCAAGAACAAGAGCAAGATCAAAATCAAGATCAAGATCAAGA------ACAAGATCA
Allele11 AAATCAAGAACAAGAGCAAGATCAAAATCAAGATCAAGATCAAGA------ACAAGATCA
Allele12 AAATCAAGAACAAGAGCAAGATCAAAATCAAGATCAAGATCAAGA------ACAAGATCA
Allele13 AAATCAAGAACAAGAGCAAGATCAAAATCAA-----------------------------
Allele14 AAATCAAGAACAAGAGCAAGATCAAAATCAAGATCAAGATCAAGA------ACAAGATCA
Allele15 AAATCAAGAACAAGAGCAAGATCAAAATCAAGATCAAGATCAAGA------ACAAGATCA
Allele16 AAATCAAGAACAAGAGCAAGATCAAAATCAAGATCAAGATCAAGA------ACAAGATCA
Allele17 AAATCAAGAACAAGAGCAAGATCAAAATCAAGATCAAGATCAAGA------ACAAGATCA
Allele18 AAATCAAGAACAAGAGCAAGATCAAAATCAAGATCAAGATCAAGA**TCAAGA**ACAAGATCA
Allele19 AAATCAAGAACAAGAGCAAGATCAAAATCAAGATCAAGATCAAGA------ACAAGATCA

Allele1 AGAACAAGATCAAGATCAAGATCAAGAACAAGATCAAGAACAAGGTCAAGAACAAGATCA
Allele2 AGA**T**CAA**A**ATCAAGATCAAGATCAAGAACAAGATCAAGAACAAGGTCAAGAACAAGATCA
Allele3 AGA**T**CAA**A**ATCAAGATCAAGATCAAGAACAAGATCAAGAACAAGGTCAAGAACAAGATCA
Allele4 AGAACAAGATCAAGATCAAGATCAAGAACAAGATCAAGAACAAGGTCAAGAACAAGATCA
Allele5 AGAACAAGATCAAGATCAAGATCAAGAACAAGATCAAGAACAAGGTCAAGAACAAGATCA
Allele6 AGA**T**CAA**A**ATCAAGATCAAGATCAAGAACAAGATCAAGAACAAGGTCAAGAACAAGATCA
Allele7 AGAACAAGATCAAGATCAAGATCAAGAACAAGATCAAGAACAAGGTCAAGAACAAGATCA
Allele8 AGA**T**CAAGATCAAGATCAAGATCAAGAACAAGATCAAGAACAAGGTCAAGAACAAGATCA
Allele9 AGA**T**CAAGATCAAGATCAAGATCAAGAACAAGATCAAGAACAAGGTCAAGAACAAGATCA
Allele10 AGAACAAGATCAAGATCAAGATCAAGAACAAGATCAAGAACAAGGTCAAGAACAAGATCA
Allele11 AGA**T**CAA**A**ATCAAGATCAAGATCAAGAACAAGATCAAGAACAAGGTCAAGAACAAGATCA
Allele12 AGA**T**CAA**A**ATCAAGATCAAGATCAAGAACAAGATCAAGAACAAGGTCAAGAACAAGATCA
Allele13 -------------GATCAAGATCAAGAACAAGATCAAGAACAAGGTCAAGAACAAGATCA
Allele14 AGA**T**CAA**A**ATCAAGATCAAGATCAAGAACAAGATCAAGAACAAGGTCAAGAACAAGATCA
Allele15 AGA**T**CAA**A**ATCAAGATCAAGATCAAGAACAAGATCAAGAACAAGGTCAAGAACAAGATCA
Allele16 AGA**T**CAA**A**ATCAAGATCAAGATCAAGAACAAGATCAAGAACAAGGTCAAGAACAAGATCA
Allele17 AGA**T**CAA**A**ATCAAGATCAAGATCAAGAACAAGATCAAGAACAAGGTCAAGAACAAGATCA
Allele18 AGA**T**CAA**A**ATCAAGATCAAGATCAAGAACAAGATCAAGAACAAGGTCAAGAACAAGATCA
Allele19 AGA**T**CAA**A**ATCAAGATCAAGATCAAGAACAAGATCAAGAACAAGGTCAAGAACAAGATCA

Allele1	AGAACAAGATCAAGATCAAACTTAGATTCTGAACTCGATTCAGGAACTAGTAAAACAGAG
Allele2	AGAACAAGATCAAGATCAAACTTAGATTCTGAACTCGATTCAGGAACTAGTAAAACAGAG
Allele3	AGAACAAGATCAAGATCAAACTTAGATTCTGAACTCGATTCAGGAACTAGTAAAACAGAG
Allele4	AGAACAAGATCAAGATCAAACTTAGATTCTGAACTCGATTCAGGAACTAGTAAAACAGAG
Allele5	AGAACAAGATCAAGATCAAACTTAGATTCTGAACTCGATTCAGGAACTAGTAAAACAGAG
Allele6	AGAACAAGATCAAGATCAAACTTAGATTCTGAACTCGATTCAGGAACTAGTAAAACAGAG
Allele7	AGAACAAGATCAAGATCAAACTTAGATTCTGAACTCGATTCAGGAACTAGTAAAACAGAG
Allele8	AGAACAAGATCAAGATCAAACTTAGATTCTGAACTCGATTCAGGAACTAGTAAAACAGAG
Allele9	AGAACAAGATCAAGATCAAACTTAGATTCTGAACTCGATTCAGGAACTAGTAAAACAGAG
Allele10	AGAACAAGATCAAGATCAAACTTAGATTCTGAACTCGATTCAGGAACTAGTAAAACAGAG
Allele11	AGAACAAGATCAAGATCAAACTTAGATTCTGAACTCGATTCAGGAACTAGTAAAACAGAG
Allele12	AGAACAAGATCAAGATCAAACTTAGATTCTGAACTCGATTCAGGAACTAGTAAAACAGAG
Allele13	AGAACAAGATCAAGATCAAACTTAGATTCTGAACTCGATTCAGGAACTAGTAAAACAGAG
Allele14	AGAACAAGATCAAGATCAAACTTAGATTCTGAACTCGATTCAGGAACTAGTAAAACAGAG
Allele15	AGA**------------**TCAAACTTAGATTCTGAACTCGATTCAGGAACTAGTAAAACAGAG
Allele16	AGAACAAGATCAAGATCAAACTTAGATTCTGAACTCGATTCAGGAACTAGTAAAACAGAG
Allele17	AGAACAAGATCAAGATCAAACTTAGATTCTGAACTCGATTCAGGAACTAGTAAAACAGAG
Allele18	AGAACAAGATCAAGATCAAACTTAGATTCTGAACTCGATTCAGGAACTAGTAAAACAGAG
Allele19	AGAACAAGATCAAGATCAAACTTAGATTCTGAACTCGATTCAGGAACTAGTAAAACAGAG

Allele1	TGTACAGAAGGTAGTTCTGTACTTGCTGAACCTTTTAAATCAAAATTTTTGAAGATGTTT
Allele2	TGTACAGAAGGTAGTTCTGTACTTGCTGAACCTTTTAAATCAAAATTTTTGAAGATGTTT
Allele3	TGTACAGAAGGTAGTTCTGTACTTGCTGAACCTTTTAAATCAAAATTTTTGAAGATGTTT
Allele4	TGTACAGAAGGTAGTTCTGTACTTGCTGAACCTTTTAAATCAAAATTTTTGAAGATGTTT
Allele5	TGTACAGAAGGTAGTTCTGTACTTGCTGAACCTTTTAAATCAAAATTTTTGAAGATGTTT
Allele6	TGTACAGAAGGTAGTTCTGTACTTGCTGAACCTTTTAAATCAAAATTTTTGAAGATGTTT
Allele7	TGTACAGAAGGTAGTTCTGTACTTGCTGAACCTTTTAAATCAAAATTTTTGAAGATGTTT
Allele8	TGTACAGAAGGTAGTTCTGTACTTGCTGAACCTTTTAAATCAAAATTTTTGAAGATGTTT
Allele9	TGTACAGAAGGTAGTTCTGTACTTGCTGAACCTTTTAAATCAAAATTTTTGAAGATGTTT
Allele10	TGTACAGAAGGTAGTTCTGTACTTGCTGAACCTT**A**TAAATCAAAATTTTTGAAGATGTTT
Allele11	TGTACAGAAGGTAGTTCTGTACTTGCTGAACCTTTTAAATCAAAATTTTTGAAGATGTTT
Allele12	TGTACAGAAGGTAGTTCTGTACTTGCTGAACCTTTTAAATCAAAATTTTTGAAGATGTTT
Allele13	TGTACAGAAGGTAGTTCTGTACTTGCTGAACCTTTTAAATCAAAATTTTTGAAGATGTTT
Allele14	TGTACAGAAGGTAGTTCTGTACTTGCTGAACCTTTTAAATCAAAATTTTTGAAGATGTTT
Allele15	TGTACAGAAGGTAGTTCTGTACTTGCTGAACCTTTTAAATCAAAATTTTTGAAGATGTTT
Allele16	TGTACAGAAGGTAGTTCTGTACTTGCTGAACCTTTTAAATCAAAATTTTTGAAGATGTTT
Allele17	TGTACAGAAGGTAGTTCTGTACTTGCTGAACCTTTTAAATCAAAATTTTTGAAGATGTTT
Allele18	TGTACAGAAGGTAGTTCTGTACTTGCTGAACCTTTTAAATCAAAATTTTTGAAGATGTTT
Allele19	TGTACAGAAGGTAGTTCTGTACTTGCTGAACCTTTTAAATCAAAATTTTTGAAGATGTTT

Allele1	GTGAAAGAGCCCTTAGAAGATTCTACTCCTTCAGTAATGAAATTTTTCAGTATTTCATAT
Allele2	GTGAAAGAGCCCTTAGAAGATTCTACTCCTTCAGTAATGAAATTTTTCAGTATTTCATAT
Allele3	GTGAAAGAGCCCTTAGAAGATTCTACTCCTTCAGTAATGAAATTTTTCAGTATTTCATAT
Allele4	GTGAAAGAGCCCTT**G**GAAGATTCTACTCCTTCAGTAATGAAATTTTTCAGTATTTCATAT
Allele5	GTGAAAGAGCCCTTAGAAGATTCTACTCCTTCAGTAATGAAATTTTTCAGTATTTCATAT
Allele6	GTGAAAGAGCCCTTAGAAGATTCTACTCCTTCAGTAATGAAATTTTTCAGTATTTCATAT
Allele7	GTGAAAGAGCCCTTAGAAGATTCTACTCCTTCAGTAATGAAATTTTTCAGTATTTCATAT
Allele8	GTGAAAGAGCCCTTAGAAGATTCTACTCCTTCAGTAATGAAATTTTTCAGTATTTCATAT
Allele9	GTGAAAGAGCCCTTAGAAGATTCTACTCCTTCAGTAATGAAATTTTTCAGTATTTCATAT
Allele10	GTGAAAGAGCCCTTAGAAGATTCTACTCCTTCAGTAATGAAATTTTTCAGTATTTCATAT
Allele11	GTGAAAGAGCCCTTAGAAGATTCTACTCCTTCAGTAATGAAATTTTTCAGTATTTCATAT
Allele12	GTGAAAGAGCCCTTAGAAGATTCTACTCCTTCAGTAATGAAATTTTTCAGTATTTCATAT
Allele13	GTGAAAGAGCCCTTAGAAGATTCTACTCCTTCAGTAATGAAATTTTTCAGTATTTCATAT
Allele14	GTGAAAGAGCCCTTAGAAGATTCTACTCCTTCAGTAATGAAATTTTTCAGTATTTCATAT
Allele15	GTGAAAGAGCCCTTAGAAGATTCTACTCCTTCAGTAATGAAATTTTTCAGTATTTCATAT
Allele16	GTGAAAGAGCCCTTAGAAGATTCTACTCCTTCAGTAATGAAATTTTTCAGTATTTCATAT
Allele17	GTGAAAGAGCCCTTAGAAGATTCTACTCCTTCAGTAATGAAATTTTTCAGTATTTCATAT
Allele18	GTGAAAGAGCCCTTAGAAGATTCTACTCCTTCAGTAATGAAATTTTTCAGTATTTCATAT
Allele19	GTGAAAGAGCCCTTAGAAGATTCTACTCCTTCAGTAATGAAATTTTTCAGTATTTCATAT

Allele1	CCAAATGAGCCAACTGTTACAGTAATGTCAGAGGCTAAAGAAATTTTTAATGAAGTTCAA
Allele2	CCAAATGAGCCAACTGTTACAGTAATGTCAGAGGCTAAAGAAATTTTTAATGAAGTTCAA
Allele3	CCAAATGAGCCAACTGTTACAGTAATGTCAGAGGCTAAAGAAATTTTTAATGAAGTTCAA
Allele4	CCAAATGAGCCAACTGTTACAGTAATGTCAGAGGCTAAAGAAATTTTTAATGAAGTTCAA
Allele5	CCAAATGAGCCAACTGTTACAGTAATGTCAGAGGCTAAAGAAATTTTTAATGAAGTTCAA
Allele6	CCAAATGAGCCAACTGTTACAGTAATGTCAGAGGCTAAAGAAATTTTTAATGAAGTTCAA
Allele7	CCAAATGAGCCAACTGTTACAGTAATGTCAGAGGCTAAAGAAATTTTTAATGAAGTTCAA
Allele8	CCAAATGAGCCAACTGTTACAGTAATGTCAGAGGCTAAAGAAATTTTTAATGAAGTTCAA
Allele9	CCAAATGAGCCAACTGTTACAGTAATGTCAGAGGCTAAAGAAATTTTTAATGAAGTTCAA
Allele10	CCAAATGAGCCAACTGTTACAGTAATGTCAGAGGCTAAAGAAATTTTTAATGAAGTTCAA
Allele11	CCAAATGAGCCAACTGTTACAGTAATGTCAGAGGCTAAAGAAATTTTTAATGAAGTTCAA
Allele12	CCAAATGAGCCAACTGTTACAGTAATGTCAGAGGCTAAAGAAATTTTTAATGAAGTTCAA
Allele13	CCAAATGAGCCAACTGTTACAGTAATGTCAGAGGCTAAAGAAATTTTTAATGAAGTTCAA
Allele14	CCAAATGAGCCAACTGTTACAGTAATGTCAGAGGCTAAAGAAATTTTTAATGAAGTTCAA
Allele15	CCAAATGAGCCAACTGTTACAGTAATGTCAGAGGCTAAAGAAATTTTTAATGAAGTTCAA
Allele16	CCAAATGAGCCAACTGTTACAGTAATGTCA**A**AGGCTAAAGAAATTTTTAATGAAGTTCAA
Allele17	CCAAATGAGCCAACTGTTACAGTAATGTCA**A**AGGCTAAAGAAATTTTTAATGAAGTTCAA
Allele18	CCAAATGAGCCAACTGTTACAGTAATGTCAGAGGCTAAAGAAATTTTTAATGAAGTTCAA
Allele19	CCAAATGAGCCAACTGTTACAGTAATGTCAGAGGCTAAAGAAATTTTTAATGAAGTTCAA

Allele1	AATTGCTTTTTGGAAACGGCCAGATT
Allele2	AATTGCTTTTTGGAAACGGCCAGATT
Allele3	AATTGCTTTTTGGAAACGGCCAGATT
Allele4	AATTGCTTTTTGGAAACGGCCAGATT
Allele5	AATTGCTTTTTGGAAACGGCCAGATT
Allele6	AATTGCTTTTTGGAAACGGCCAGATT
Allele7	AATTGCTTTTTGGAAACGGCCAGATT
Allele8	AATTGCTTTTTGGAAACGGCCAGATT
Allele9	AATTGCTTTTTGGAAACGGCCAGATT
Allele10	AATTGCTTTTTGGAAACGGCCAGATT
Allele11	AATTGCTTTTTGGAAACGGCCAGATT
Allele12	AATTGCTTTTTGGAAACGGCCAGATT
Allele13	AATTGCTTTTTGGAAACGGCCAGATT
Allele14	AATTGCTTTTTGGAAACGGCCAGATT
Allele15	AATTGCTTTTTGGAAACGGCCAGATT
Allele16	AATTGCTTTTTGGAAACGGCCAGATT
Allele17	AATTGCTTTTTGGAAACGGCCAGATT
Allele18	AATTGCTTTTTGGAAACGGCCAGATT
Allele19	AATTGCTTTTTGGAAACGGCCAGATT

MARKER CPATCC_0024650 (chromosome 5)

Allele1	AGAGCAACTTTCCATCTCACTTTTAGGTGGAAGCTTGGAAATAAGGGATTTAAATATTAG
Allele2	AGAGCAACTTTCCATCTCACTTTTAGGTGGAAGCTTGGAAATAAGGGATTTAAATATTAG
Allele3	AGAGCAACTTTCCATCTCACTTTTAGGTGGAAGCTTGGAAATAAGGGATTTAAATATTAG
Allele4	AGAGCAACTTTCCATCTCACTTTTAGGTGGAAGCTTGGAAATAAGGGATTTAAATATTAG
Allele5	AGAGCAACTTTCCATCTCACTTTTAGGTGGAAGCTTGGAAATAAGGGATTTAAATATTAG
Allele6	AGAGCAACTTTCCATCTCACTTTTAGGTGGAAGCTTGGAAATAAGGGATTTAAATATTAG
Allele7	AGAGCAACTTTCCATCTCACTTTTAGGTGGAAGCTTGGAAATAAGGGATTTAAATATTAG

Allele1	AAAAGAACTTTTTGATGATTTATCTTTCCCAATCAGTTTAAGTGATGGAATTGTTGGAAA
Allele2	AAAAGAACTTTTTGAT**T**ATTTATCTTTCCCAATCAGTTTAAGTGATGGAATTGTTGGAAA
Allele3	AAAAGAACTTTTTGATGATTTATCTTTCCCAATCAGTTTAAGTGATGGAATTGTTGGAAA
Allele4	AAAAGAACTTTTTGATGATTTATCTTTCCCAATCAGTTTAAGTGATGGAATTGTTGGAAA
Allele5	AAAAGAACTTTTTGATGATTTATCTTTCCCAATCAGTTTAAGTGATGGAATTGTTGGAAA
Allele6	AAAAGAACTTTTTGATGATTTATCTTTCCCAATCAGTTTAAGTGATGGAATTGTTGGAAA
Allele7	AAAAGAACTTTTTGATGATTTATCTTTCCCAATCAGTTTAAGTGATGGAATTGTTGGAAA

Allele1	AGTTAATATTGATGTTATCTGGAGAAAAATATTTACCCAAGAGTTTGTGAAAATCACCTT
Allele2	AGTTAATATTGATGTTATCTGGAGAAAAATATTTACCCAAGAGTTTGTGAAAATCACCTT
Allele3	AGTTAATATTGATGTTATCTGGAGAAAAATATTTACCCAAGAGTTTGTGAAAATCACCTT
Allele4	AGTTAATATTGATGTTATCTGGAGAAAAATATTTACCCAAGAGTTTGTGAAAATCACCTT
Allele5	AGTTAATATTGATGTTATCTGGAGAAAAATATTTACCCAAGAGTTTGTGAAAATCACCTT
Allele6	AGTTAATATTGATGTTATCTGGAGAAAAATATTTACCCAAGAGTTTGTGAAAATCACCTT
Allele7	AGTTAATATTGATGTTATCTGGAGAAAAATATTTACCCAAGAGTTTGT**A**AAAATCACCTT
Allele1	GGATGATGTATATGTTATTTTTAATACTACTGATATGAAAAATTGGAATGTTGAAATGTT
Allele2	GGATGATGTATATGTTATTTTTAATACTACTGATATGAAAAATTGGAATGTTGAAATGTT
Allele3	GGATGATGTATATGTTATTTTTAATACTACTGATATGAAAAATTGGAATGTTGAAATGTT
Allele4	GGATGATGTATATGTTATTTTTAATACTACTGATATGAAAAATTGGAATGTTGAAATGTT
Allele5	GGATGATGTATATGTTATTTTTAATACTACTGATATGAAAAATTGGAATGTTGAAATGTT
Allele6	GGATGATGTATATGTTATTTTTAATACTACTGATATGAAAAATTGGAATGTTGAAATGTT
Allele7	GGATGATGTATATGTTATTTTTAATACTACTGATATGAAAAATTGGAATGTTGAAATGTT
Allele1	TGAAAAAAACTGGAAAAGGGTTAAAGCTAATCTATTAAAACAAGATGAATTTATTACTTT
Allele2	TGAAAAAAACTGGAAAAGGGTTAAAGCTAATTTATTAAAACAAGATGAATTTATTACTTT
Allele3	TGAAAAAAACTGGAAAAGGGTTAAAG**T**TAATCTATTAAAACAAGATGAATTTATTACTTT
Allele4	TGAAAAAAACTGGAAAAGGGTTAAAGCTAAT**T**TATTAAAACAAGATGAATTTATTACTTT
Allele5	TGAAAAAAACTGGAAAAGGGTTAAAGCTAATCTATTAAAACAAGATGAATTTATTACTTT
Allele6	TGAAAAAAACTG**A**AAAAGGGTTAAAGCTAATCTATTAAAACAAGATGAATTTATTACTTT
Allele7	TGAAAAAAACTGGAAAAGGGTTAAAGCTAATCTATTAAAACAAGATGAATTTATTACTTT
Allele1	CTTAAAAAGTGCTATGGCTTCCAATTTTCTCAAACAAATTGGACACTTTTTTATATCAAA
Allele2	CTTAAAAAGTGCTATGGCTTCCAATTTTCTCAAACAAATTGGACACTTTTTTATATCAAA
Allele3	CTTAAAAAGTGCTATGGCTTCCAATTTTCTCAAACAAATTGGACACTTTTTTATATCAAA
Allele4	CTTAAAAAGTGCTATGGCTTCCAATTTTCTCAAACAAATTGGACACTTTTTTATATCAAA
Allele5	CTTAAAAAGTGCTATGGCTTCCAATTTTCTCAAACAAATTGGACACTTTTTTATATCAA**C**
Allele6	CTTAAAAAGTGCTATGGCTTCCAATTTTCTCAAACAAATTGGACACTTTTTTATATCAAA
Allele7	CTTAAAAAGTGCTATGGCTTCCAATTTTCTCAAACAAATTGGACACTTTTTTATATCAAA
Allele1	GATTCAGTTTGAAATTAAAAACATCAATTTTAGAATTGAAAATTTGTTATTCCACTATAT
Allele2	GATTCAGTTTGAAATTAAAAACATCAATTTTAGAATTGAAAATTTGTTATTCCACTATAT
Allele3	GATTCAGTTTGAAATTAAAAACATCAATTTTAGAATTGAAAATTTGTTATTCCACTATAT
Allele4	GATTCAGTTTGAAATTAAAAACATCAATTTTAGAATTGAAAATTTGTTATTCCACTATAT
Allele5	GATTCAGTTTGAAATTAAAAACATCAATTTTAGAATTGAAAATTTGTTATTCCACTATAT
Allele6	GATTCAGTTTGAAATTAAAAACATCAATTTTAGAATTGAAAATTTGTTATTCCACTATAT
Allele7	GATTCAGTTTGAAATTAAAAACATCAATTTTAGAATTGAAAATTTGTTATTCCACTATAT
Allele1	GAAGAGATTTGTTATTGGACTAAGTATTGATAAAATTTCCAGTCGAAATTGTAATGAATA
Allele2	GAAGAGATTTGTTATTGGACTAAGTATTGATAAAATTTCCAGTCGAAATTGTAATGAATA
Allele3	GAAGAGATTTGTTATTGGACTAAGTATTGATAAAATTTCCAGTCGAAATTGTAATGAATA
Allele4	GAAGAGATTTGTTATTGGACTAAGTATTGATAAAATTTCCAGTCGAAATTGTAATGAATA
Allele5	GAAGAGATTTGTTATTGGACTAAGTATTGATAAAATTTCCAGTCGAAATTGTAATGAATA
Allele6	GAAGAGATTTGTTATTGGACTAAGTATTGATAAAATTTCCAGTCGAAATTGTAATGAATA
Allele7	GAAGAGATTTGTTATTGGACTAAGTATTGATAAAATTTCCAGTCGAAATTGTAATGAATA
Allele1	TTGGATTCCAGTAGATAAATCTGGAGGAGCCATTTTAGGCCGTAAT
Allele2	TTGGATTCCAGTAGATAAATCTGGAGGAGCCATTTTAGGCCGTAAT
Allele3	TTGGATTCCAGTAGATAAATCTGGAGGAGCCATTTTAGGCCGTAAT
Allele4	TTGGATTCCAGTAGATAAATCTGGAGGAGCCATTTTAGGCCGTAAT
Allele5	TTGGATTCCAGTAGATAAATCTGGAGGAGCCATTTTAGGCCGTAAT
Allele6	TTGGATTCCAGTAGATAAATCTGGAGGAGCCATTTTAGGCCGTAAT
Allele7	TTGGATTCCAGTAGATAAATCTGGAGGAGCCATTTTAGGCCGTAAT

MARKER CPATCC_0012400 (chromosome 6)

Allele1 CCTACACGTCCTTATGGCCCTGGTGAAAAGTTACCTCCACATCTATCTCCATTTGTGGAT
Allele2 CCTACACGTCCTTATGGCCCTGGTGAAAAGTTACCTCCACATCTATCTCCATTTGTGGAT
Allele3 CCTACACGTCCTTATGGCCCTGGTGAAAAGTTACCTCCACATCTATCTCCATTTGTGGAT
Allele4 CCTACACGTCCTTATGGCCCTGGTGAAAAGTTACCTCCACATCTATCTCCATTTGTGGAT
Allele5 CCTACACGTCCTTATGGCCCTGGTGAAAAGTTACCTCCACATCTATCTCCATTTGTGGAT
Allele6 CCTACACGTCCTTATGGCCCTGGTGAAAAGTTACCTCCACATCTATCTCCATTTGTGGAT
Allele7 CCTACACGTCCTTATGGCCCTGGTGAAAAGTTACCTCCACATCTATCTCCATTTGTGGAT

Allele1 GATAGTACCCAAGGTTATATTCCAACTCAGAGACAAGTTCTTGATGAAATTAAGGAATCC
Allele2 GATAGTACCCAAGGTTATATTCCAACTCAGAGACAAGTTCTTGATGAAATTAAGGAATCC
Allele3 GATAGTACCCAAGGTTATATTCCAACTCAGAGACAAGTTCTTGATGAAATTAAGGAATCC
Allele4 GATAGTACCCAAGGTTATATTCCAACTCAGAGACAAGTTCTTGATGAAATTAAGGAATCC
Allele5 GATAGTACCCAAGGTTATATTCCAACTCAGAGACAAGTTCTTGATGAAATTAAGGAATCC
Allele6 GATAGTACCCAAGGTTATATTCCAACTCAGAGACAAGTTCTTGATGAAATTAAGGAATCC
Allele7 GATAGTACCCAAGGTTATATTCCAACTCAGAGACAAGTTCTTGATGAAATTAAGGAATCC

Allele1 AATAGTCATAAATCTCAATCTTGCTTATCTGAAGATGAATCAACAGAAATTGATGAACTT
Allele2 AATAGTCATAAATCTCAATCTTGCTTATCTGAAGATGAATCAACAGAAATTGATGAACTT
Allele3 AATAGTCATAAATCTCAATCTTGCTTATCTGAAGATGAATCAACAGAAATTGATGAACTT
Allele4 AATAGTCATAAATCTCAATCTT**A**CTTATCTGAAGATGAATCAACAGAAATTGATGAACTT
Allele5 AATAGTCATAAATCTCAATCTTGCTTATCTGAAGATGAATCAACAGAAATTGATGAACTT
Allele6 AATAGTCATAAATCTCAATCTTGCTTATCTGAAGATGAATCAACAGAAATTGATGAACTT
Allele7 AATAGTCATAAATCTCAATCTTGCTTATCTGAAGATGAATCAACAGAAATTGATGAACTT

Allele1 TCAGAACATGATTCTGATATTGAAGTTCAACAAGCTAGAGAAGATGCCTACTTTGATTCT
Allele2 TCAGAACATGATTCTGATATTGAAGTTCAACAAGCTAGAGAAGATGCCTACTTTGATTCT
Allele3 TCAGAACATGATTCTGATATTGAAGTTCAACAAGCTAGAGAAGATGCCTACTTTGATTCT
Allele4 TCAGAACATGATTCTGATATTGAAGTTCAACAAGCTAGAGAAGATGCCTACTTTGATTCT
Allele5 TCAGAACATGATTCTGATATTGAAGTTCAACAAGCTAGAGAAGATGCCTACTTTGATTCT
Allele6 TCAGAACATGATTCTGATATTGAAGTTCAACAAGCTAGAGAAGATGCCTACTTTGATTCT
Allele7 TCAGA**G**CATGATTCTGATATTGAAGTTCAACAAGCTAGAGAAGATGCCTACTTTGATTCT

Allele1 ATTGAGAGAGAACAATCATTATCAACCTCAAATGAAATTGATTCAATTGATAACGAGCAT
Allele2 ATTGAGAGAGAACAATCATTATCAACCTCAAATGAAATTGATTCAATTGATAACGAGCAT
Allele3 ATTGAGAGAGAACAATCATTATCAACCTCAAATGAAATTGATTCAATTGATAACGAGCAT
Allele4 ATTGAGAGAGAACAATCATTATCAACCTCAAATGAAATTGATTCAATTGATAACGAGCAT
Allele5 ATTGAGAGAGAACAATCATTATCAACCTCAAATGAAATTGATTCAATTGATAACGAGCAT
Allele6 ATTGAGAGAGAACAATCATTATCAACCTCAAATGAAATTGATTCAATTGATAACGAGCAT
Allele7 ATTGAGAGAGAACAATCATTATCAACCTCAAATGAAATTGATTCAATTGATAACGAGCAT

Allele1 AAAGATTCATCCAATTTTACAACAGAATCTGAATTAACTAATACAAAAGATAAAGTAAAT
Allele2 AAAGATTCATCCAATTTTACAACAGAATCTGAATTAACTAATACAAAAGATAAAGTAAAT
Allele3 AAAGATTCATCCAATTTTACAACAGAATCTGAATTAACTAATACAA**G**AGATAAAGTAAAT
Allele4 AAAGATTCATCCAATTTTACAACAGAATCTGAATTAACTAATACAAAAGATAAAGTAAAT
Allele5 AAAGATTCATCCAATTTTACAACAGAATCTGAATTAACTAATACAAAAGATAAAGTAAAT
Allele6 AAAGATTCATCCAATTTTACAACAGAATCTGAATTAACTAATACAAAAGATAAAGTAAAT
Allele7 AAAGATTCATCCAATTTTACAACAGAATCTGAATTAACTAATACAAAAGATAAAGTAAAT

Allele1 ATTGCTAGAAAGCTCGCGAAGAAACGTAAAGAAGAGGAACAAAGAGAACAACAAAAGACT
Allele2 ATTGCTAGAAAGCTCGCGAAGAAACGTAAAGAAGAGGAACAAAGAGAACAACAAAAGACT
Allele3 ATTGCTAGAAAGCTCGCGAAGAAACGTAAAGAAGAGGAACAAAGAGAACAACAAAAGACT
Allele4 ATTGCTAGAAAGCTCGCGAAGAAACGTAAAGAAGAGGAACAAAGAGAACAACAAAAGACT
Allele5 ATTGCTAGAAAGCTCGCGAAGAAACGTAAAGAAGAGGAACAAAGAGAACAACAAAAGACT
Allele6 ATTGCTAGAAAGCTCGCGAAGAAACGTAAAGAAGAGGAACAAAGAGAACAACAAAAGACT
Allele7 ATTGCTAGAAAGCTCGCGAAGAAACGTAAAGAAGAGGAACAAAGAGAACAACAAAAGACT

Allele1	CTACTCAAAAAGAAGCATAAGAGACTTTTACAAAGAATTGAATATTCTAACAAAATCGCT
Allele2	CTACTCAAAAAGAAGCATAAGAG**G**CTTTTACAAAGAATTGAATATTCTAACAAAAT**T**GCT
Allele3	CTACTCAAAAAGAAGCATAAGAG**G**CTTTTACAAAGAATTGAATATTCTAACAAAAT**T**GCT
Allele4	CTACTCAAAAAGAAGCATAAGAG**G**CTTTTACAAAGAATTGAATATTCTAACAAAAT**T**GCT
Allele5	CTACTCAAAAAGAAGCATAAGAG**G**CTTTTACAAAGAATTGAATATTCTAACAAAATCGCT
Allele6	CTACTCAAAAAGAAGCATAAGAGACTTTTACAAAGAATTGAATATTCTAACAAAAT**T**GCT
Allele7	CTACTCAAAAAGAAGCATAAGAG**G**CTTTTACAAAGAATTGAATATTCTAACAAAAT**T**GCT

Allele1	TCGGAGAAAGCAGAAAGGTTGGA
Allele2	TCGGAGAAAGCAGAAAGGTTGGA
Allele3	TCGGAGAAAGCAGAAAGGTTGGA
Allele4	TCGGAGAAAGCAGAAAGGTTGGA
Allele5	TCGGAGAAAGCAGAAAGGTTGGA
Allele6	TCGGAGAAAGCAGAAAGGTTGGA
Allele7	TCGGAGAAAGCAGAAAGGTTGGA

MARKER CPATCC_007000 (chromosome 7)

Allele1	GACTACAACTGGAAGCTCTTAGTAGTATACTGGTACTCATAAAGTTAATAGAAGCTGGAA
Allele2	GACTACAACTGGAAGCTCTTAGTAGTATACTGGTACTCATAAAGTTAATAGAAGCTGGAA
Allele3	GACTACAACTGGAAGCTCTTAGTAGTATACTGGTACTCATAAAGTTAATAGAAGCTGGAA
Allele4	GACTACAACTGGAAGCTCTTAGTAGTATACTGG**C**ACTCATAAAGTTAATAGAAGCTGGAA
Allele5	GACTACAACTGGAAGCTCTTAGTAGTATACTGGTACTCATAAAGTTAATAGAAGCTGGAA

Allele1	TTGAAAGGATTATTCTGTTTCCTGGATGGTTTACTATGGAGTTAAGAGATTTAAGGTAAT
Allele2	TTGAAAGGATTATTCTGTTTCCTGGATGGTTTACTATGGAGTTAAGAGATTTAAGGTAAT
Allele3	TTGAAAGGATTATTCTGTTTCCTGGATGGTTTACTATGGAGTTAAGAGATTTAAGGTAAT
Allele4	TTGAAAGGATTATTCTGTTTCCTGGATGGTTTACTATGGAGTTAAGAGATTTAAGGTAAT
Allele5	TTGAAAGGATTATTCTGTTTCCTGGATGGTTTACTATGGAGTTAAGAGATTTAAGGTAAT

Allele1	AGACCAATAAGTTAACATGAGGAACAAAAGATTCTTGTAGGCCTGTAAATCCAAGATTCT
Allele2	AGACCAATAAGTTAACATGAGGAACAAAAGATTCTTGTAGGCCTGTAAATCCAAGATTCT
Allele3	AGACCAATAAGTTAACATGAGGAACAAAAGATTCTTGTAGGCCTGTAAATCCAAGATTCT
Allele4	AGACCAATAAGTTAACATGAGGAACAAAAGATTCTTGTAGGCCTGTAAATCCAAGATTCT
Allele5	AGACCAATAAGTTAACATGAGGAACAAAAGATTCTTGTAGGCCTGTAAATCCAAGATTCT

Allele1	TGAATGATGAAGTAATTAAACCAGTATTTTGGGATACATGTCCAAACTCTGGGACTGTTG
Allele2	TGAATGATGAAGTAATTAAACCAGTATTTTGGGATACATGTCCAAACTC**C**GGGACTGTTG
Allele3	TGAATGATGAAGTAATTAAACCAGTATTTTGGGATACATGTCCAAACTC**C**GGGACTGTTG
Allele4	TGAATGATGAAGTAATTAAACCAGTATTTTGGGATACATGTCCAAACTCTGGGACTGTTG
Allele5	TGAATGATGAAGTAATTAAACCAGTATTTTGGGATACATGTCCAAACTC**C**GGGACTGTTG

Allele1	AGAAATAGCTTGGCCTTTGTTTAAACTTAAAGTTTGAGAAGCAATCTGCGATTAATTGTG
Allele2	AGAAATAGCTTGGCCTTTGTTTAAACTTAAAGTTTGAGAAGCAATCTGCGATTAATTGTG
Allele3	AGAAATAGCTTGGCCTTTGTTTAAACTTAAAGT**C**TGAGAAGCAATCTGCGATTAATTGTG
Allele4	AGAAATAGCTTGGCCTTTGTTTAAACTTAAAGTTTGAGAAGCAATCTGCGATTAATTGTG
Allele5	AGAAATAGCTTGGCCTTTGTTTAAACTTAAAGT**C**TGAGAAGCAATCTGCGATTAATTGTG

Allele1	GCTTTGAGATGAATTCTCTGGCAAAGTTAATTAAGATATGATTCAAAAGGAGACTAAACA
Allele2	GCTTTGAGATGAATTCTCTGGCAAAGTTAATTAAGATATGATTCAAAAGGAGACTAAACA
Allele3	GCTTTGAGATGAATTCTCTGGCAAAGTTAATTAAGATATGATTCAAAAGGAGACTAAACA
Allele4	GCTTTGAGATGAATTCTCTGGCAAAGTTAATTAAGATATGATTCAAAAGGAGACTAAACA
Allele5	GCTTTGAGATGAATTCTCTGGCAAAGTTAATTAAGATATGATTCAAAAGGAGACTAAACA

Allele1	TGTCTGTTTCTGTAATTTTCAATCCAGATCTTTGGAGGGACAAAATAATCTCAGCTGAGT
Allele2	TGTCTGTTTCTGTAATTTTCAATCCAGATCTTTGGAGGGACAAAATAATCTCAGCTGAGT
Allele3	TGTCTGTTTCTGTAATTTTCAATCCAGATCTTTGGAGGGACAAAATAATCTCAGCTGAGT
Allele4	TGTCTGTTTCTGTAATTTTCAATCCAGATCTTTGGAGGGACAAAATAATCTCAGCTGAGT
Allele5	TGTCTGTTTCTGTAATTTTCAATCCAGATCTTTGGAGGGACAAAATAATCTCAGCTGAGT

Allele1	AATACTCATAGAATAGGAATTCAGGATTAGTATCGTAAACACATAACTTGAGAACTGAGA
Allele2	AATACTCATAGAATAGGAATTCAGGATTAGTATCGTAAACACATAACTTGAGAACTGAGA
Allele3	AATACTCATAGAATAGGAATTCAGGATTAGTATCGTAAACACATAACTTGAGAACTGAGA
Allele4	AATACTCATAGAATAGGAATTCAGGATTAGTATCGTAAACACATAACTTGAGAACTGAGA
Allele5	AATACTCATAGAATAGGAATTCAGGATTAGTATCGTAAACACATAACTTGAGAACTGAGA

Allele1	TCTTTCGTAGGAAATCAGAAGAAATCTTGTTGAGATCTTTAGCTGACTTTAAAATTTGTA
Allele2	TCTTTCGTAGGAAATCAGAAGAAATCTTGT**C**GAGATCTTTAGCTGACTTTAAAATTTGTA
Allele3	TCTTTCGTAGGAAATCAGAAGAAATCTTGT**C**GAGATCTTTAGCTGACTTTAAAATTTGTA
Allele4	TCTTTCGTAGGAAATCAGAAGAAATCTTGTTGAGATCTTTAGCTGACTTTAAAATTTGTA
Allele5	TCTTTCGTAGGAAATCAGAAGAAATCTTGTTGAGATCTTTAGCTGACTTTAAAATTTGTA

Allele1	TTGGCTCAAAGAGTTTCC
Allele2	TTGGCTCAAAGAGTTTCC
Allele3	TTGGCTCAAAGAGTTTCC
Allele4	TTGGCTCAAAGAGTTTCC
Allele5	TTGGCTCAAAGAGTTTCC

MARKER CPATCC_001010 (chromosome 8)

Allele1	TAACTAGAGCAATCCCAGTAACAACACCACATGAACAGGTAATTACTACTGTCGATAACA
Allele2	TAACTAGAGCAATCCCAGTAACAACACCACATGAACAGGTAATTACTACTGTCGATAACA
Allele3	TAACTAGAGCAATCCCAGTAACAACACCACATGAACAGGTAATTACTACTGTCGATAACA
Allele4	TAACTAGAGCAATCCCAGTAACAACACCACATGAACAGGTAATTACTACTGTCGATAACA
Allele5	TAACTAGAGCAATCCCAGTAACAACACCACATGAACAGGTAATTACTACTGTCGATAACA
Allele6	TAACTAGAGCAATCCCAGTAACAACACCACATGAACAGGTAATTACTACTGTCGATAACA
Allele7	TAACTAGAGCAATCCCAGTAACAACACCACATGAACAGGTAATTACTACTGTCGATAACA
Allele8	TAACTAGAGCAATCCCAGTAACAACACCACATGAACAGGTAATTACTACTGTCGATAACA
Allele9	TAACTAGAGCAATCCCAGTAACAACACCACATGAACAGGTAATTACTACTGTCGATAACA

Allele1	CACAAACATATTCTGTTAGAGAGGTTAGAACAGAAGACAATGAAATTCGCCGTTCTTCTC
Allele2	CACAAACATATTCTGTTAGAGAGGTTAGAACAGAAGACAATGAAATTCGCCGTTCTTCTC
Allele3	CACAAACATATTCTGTTAGAGAGGTTAGAACAGAAGACAATGAAATTCGCCGTTCTTCTC
Allele4	CACAAACATATTCTGTTAGAGAGGTTAGAACAGAAGACAATGAAATTCGCCGTTCTTCTC
Allele5	CACAAACATATTCTGTTAGAGAGGTTAGAACAGAAGACAATGAAATTCGCCGTTCTTCTC
Allele6	CACAAACATATTCTGTTAGAGAGGTTAGAACAGAAGACAATGAAATTCGCCGTTCTTCTC
Allele7	CACAAACATATTCTGTTAGAGAGGTTAGAACAGAAGACAATGAAATTCGCCGTTCTTCTC
Allele8	CACAAACATATTCTGTTAGAGAGGTTAGAACAGAAGACAATGAAATTCGCCGTTCTTCTC
Allele9	CACAAACATATTCTGTTAGAGAGGTTAGAACAGAAGACAATGAAATTCGCCGTTCTTCT**A**

Allele1	AGTATAATAGTGCTAATGTGAAGCCAATAAACTTGCATGAAAACCAAATAGGAACAGAAA
Allele2	AGTATAATAGTGCTAATGTGAAGCCAATAAACTTGCATGAAAACCAAATAGGAACAGAAA
Allele3	AGTATAATAGTGCTAATGTGAAGCCAATAAACTTGCATGAAAACCAAATAGGAACAGAAA
Allele4	AGTATAATAGTGCTAATGTGAAGCCAATAAACTTGCATGAAAACCAAATAGGAACAGAAA
Allele5	AGTATAATAGTGCTAATGTGAAGCCA**G**TAAACTTGCATGAAAACCAAATAGGAACAGAAA
Allele6	AGTATAATAGTGCTAATGTGAAGCCAATAAACTTGCATGAAAACCAAATAGGAACAGAAA
Allele7	AGTATAATAGTGCTAATGTGAAGCCAATAAACTTGCATGAAAACCAAATAGGAACAGAAA
Allele8	AGTATAATAGTGCTAATGTGAAGCCAATAAACTTGCATGAAAACCAAATAGGAACAGAAA
Allele9	AGTATAATAGTGCTAATGTGAAGCCAATAAACTTGCATGAAAACCAAATAGGAACAGAAA

Allele1	TTAACAATACAAATGGAAATTTTGAAACTAGAAGAAAGTCTAGCAACCTTATATTAATTA
Allele2	TTAACAATACAAATGGAAATTTTGAAACTAGAAGAAAGTCTAGCAACCTTATATTAATTA
Allele3	TTAACAATACAAATGGAAATTTTGAAAC**A**AGAAGAAAGTCTAGCAACCTTATATTAATTA
Allele4	TTAACAATACAAATGGAAATTTTGAAACTAGAAGAAAGTCTAGCAACCTTATATTAATTA
Allele5	TTAACAATACAAATGGAAATTTTGAAACTAGAAGAAAGTCTAGCAACCTTATATTAATTA
Allele6	TTAACAATACAAATGGAAATTTTGAAACTAGAAGAAAGTCTAGCAACCTTATATTAATTA
Allele7	TTAACAATACAAATGGAAATTTTGAAACTAGAAGAAAGTCTAGCAACCTTATATTAATTA
Allele8	TTAACAATACAAATGGAAATTTTGAAACTAGAAGAAAGTCTAGCAACCTTATATTAATTA
Allele9	TTAACAATACAAATGGAAATTTTGAAACTAGAAGAAAGTCTAGCAACCTTATATTAATTA

Allele1	GAGACATTGAGACTCAGAGCCTTAAAGATAAGAATGATAACCACAGTCTTACTATAGAAA
Allele2	GAGACATTGAGACTCAGAGCCTTAAAGATAAGAATGATAACCACA**A**TCTTACTATAGAAA
Allele3	GAGACATTGAGACTCAGAGCCTTAAAGATAAGAATGATAACCACAGTCTTACTATAGAAA
Allele4	GAGACATTGAGACTCAGAGCCTTAAAGATAAGAATGATAACCACAG**C**CTTACTATAGAAA
Allele5	GAGACATTGAGACTCAGAGCCTTAAAGATAAGAATGATAACCACA**A**TCTTACTATAGAAA
Allele6	GAGACATTGAGACTCAGAGCCTTAAAGATAAGAATGATAACCACAGTCTTACTATAGAAA
Allele7	GAGACATTGAGACTCAGAGCCTTAAAGATAAGAATGATAACCACAGTCTTACTATAGAAA
Allele8	GAGA**A**ATTGAGACTCAGAGCCTTAAAGATAAGAATGATAACCACA**A**TCTTACTATAGAAA
Allele9	GAGACATTGAGACTCAGAGCCTTAAAGATAAGAATGATAACCACAGTCTTACTATAGAAA

Allele1	AACAAGGAACAGAGGAAGAAAAAAGAAACTCAAATGACTATCATCACCTCCGCCTCCATC
Allele2	AAC**G**AGGAACAGAGGAAGAAAAAAGAAACTCAAATGACTATCATCACCTCCGCCTCCATC
Allele3	AACAAGGAACAGAGGAAGAAAAAAGAAACTCAAATGACTATCATCACCTCCGCCTCCATC
Allele4	AACAAGGAACAGAGGAAGAAAAAAGAAACTCAAATGACTATCATCACCTCCGCCTCCATC
Allele5	AAC**G**AGGAACAGAGGAAGAAAAAAGAAACTCAAATGACTATCATCACCTCCGCCTCCATC
Allele6	AACAAGGAACAGAGGAAGAAAAAAGAAACTCAAATGACTAT**T**ATCACCTCCGCCTCCATC
Allele7	AACAAGGAACAGAGGAAGAAAAAAGAAACTCAAATGACTATCATCACCTCCGCCTCCATC
Allele8	AAC**G**AGGAACAGAGGAAGAAAAAAGAAACTCAAATGACTATCATCACCTCCGCCTCCATC
Allele9	AACAAGGAACAGAGGAAGAAAAAAGAAACTCAAATGACTATCATCACCTCCGCCTCCATC

Allele1	ATCATATTCCAACCAAAAACCAAGCAAATTTAGTTCCTCATATTGGTAAAGTATGCGAAG
Allele2	ATCATATTCCAACCAAAAACCAAGCAAATTTAGTTCCTCATATTGGTAAAGTATGCGAAG
Allele3	ATCATATTCCAACCAAAAACCAAGCAAATTTAGTTCCTCATATTGGTAAAGTATGCGAAG
Allele4	ATCATATTCCAACCAAAAACCAAGCAAATTTAGTTCCTCATATTGGTAAAGTATGCGAAG
Allele5	ATCATATTCCAACCAAAAACCAAGCAAATTTAGTTCCTCATATTGGTAAAGTATGCGAAG
Allele6	ATCATATTCCAACCAAAAACCAAGCAAATTTAGTTCCTCATATTGGTAAAGTATGCGAAG
Allele7	**G**TCATATTCCAACCAAAAACCAAGCAAATTTAGTTCCTCATATTGGTAAAGTATGCGAAG
Allele8	ATCATATTCCAACCAAAAACCAAGCAAATTTAGTTCCTCATATTGGTAAAGTATG**T**GAAG
Allele9	ATCATATTCCAACCAAAAACCAAGCAAATTTAGTTCCTCATATTGGTAAAGTATGCGAAG

Allele1	AAAAAGAATCTGAAATTCTTAACAGGGAATTTATACAAAGAAATAACAACCATGAATATA
Allele2	AAAAAGAATCTGAAATTCTTAACAGGGAATTTATACAAAGAAATAACAACCATGAATATA
Allele3	AAAAAGAATCTGAAATTCTTAACAGGGAATTTATACAAAGAAATAACAACCATGAATATA
Allele4	AAAAAGAATCTGAAATTCTTAACAGGGAATTTATACAAAGAAATAACAACCATGAATATA
Allele5	AAAAAGAATCTGAAATTCTTAACAGGGAATTTATACAAAGAAATAACAACCATGAATATA
Allele6	AAAAAGAATCTGAAATTCTTAACAGGGAATTTATACAAAGAAATAACAACCATGAATATA
Allele7	AAAAAGAATCTGAAATTCTTAACAGGGAATTTATACAAAGAAATAACAACCATGAATATA
Allele8	AAAAAGAATCTGAAATTCTTAACAGGGAATTTATACAAAGAAATAACAACCATGAATATA
Allele9	AAAAAGAATCTGAAATTCTTAACAGGGAATTTATACAAAGAAATAACAACCATGAATATA

Allele1	TTGAAACACAATCTTCACCTCATCAAAACACTAAGAAAATACAAAAAAAGAAGCCTCCAA
Allele2	TTGAAACACAATCTTCACCTCATCAAAACACTAAGAAAATACAAAAAAAGAAGCCTCCAA
Allele3	TTGAAACACAATCTTCACCTCATCAAAACACTAAGAAAATACAAAAAAAGAAGCCTCCAA
Allele4	TTGAAACACAATCTTCACCTCATCAAAACACTAAGAAAATACAAAAAAAGAAGCCTCCAA
Allele5	TTGAAACACAATCTTCACCTCATCAAAACACTAAGAAAATACAAAAAAAGAAGCCTCCAA
Allele6	TTGAAACACAATCTTCACCTCATCAAAACACTAAGAAAATACAAAAAAAGAAGCCTCCAA
Allele7	TTGAAACACAATCTTCACCTCATCAAAACACTAAGAAAATACAAAAAAAGAAGCCTCCAA
Allele8	TTGAAACACAATCTTCACCTCATCAAAACACTAAGAAAATACAAAAAAAGAAGCCTCCAA
Allele9	TTGAAACACAATCTTCACCTCATCAAAACACTAAGAAAATACAAAAAAAGAAGCCTCCAA

Allele1	TATCTCTTAGGTC
Allele2	TATCTCTTAGGTC
Allele3	TATCTCTTAGGTC
Allele4	TATCTCTTAGGTC
Allele5	TATCTCTTAGGTC
Allele6	TATCTCTTAGGTC
Allele7	TATCTCTTAGGTC
Allele8	TATCTCTTAGGTC
Allele9	TATCTCTTAGGTC
