# Supplementary material for: Design, development, and testing of a new multi-locus sequence typing scheme for the zoonotic pathogen Cryptosporidium parvum
Source: Curr Res Parasitol Vector Borne Dis. 2025 Aug 14;8:100308. doi: 10.1016/j.crpvbd.2025.100308 (PMC12446620; doi:10.1016/j.crpvbd.2025.100308)
Supplement: Multimedia component 5 [file mmc5.pdf]

Supplementary file 5

Multiple alignments of the *C. parvum* IOWA-ATCC and *C. hominis* orthologous sequences (retrieved from CryptoDB) of the eight markers included in the typing scheme. Primer sequences are boxed in blue, to indicate the sequence of the primer used for primary PCR, or red color to indicate additional nucleotides at the 3‘-end of the primers used for nested PCR. Differences in the primer sequences between *C. parvum* and *C. hominis* are shown in bold.

CPATCC_0039030

```
CPATCC_0039030      GGCTTTAGATACAGAACTTCTAATTTTAAGGAATTGAACTCTACTGTCAGCATGGAAAAT      1620
CHUDEA1_370         GGCTTTAGATACAGAACTTCTAATTTTAAGGAATTGAACTCTACTGTCAGCATGGAAAAT      1620
GY17_00001034       GGCTTTAGATACAGAACTTCTAATTTTAAGGAATTGAACTCTACTGTCAGCATGGAAAAT      1620
                    ************************************************************

CPATCC_0039030      GAATGCAATACACAACAATTGCTTATATCGTTGGGTAAGCAAAAAAAAGAAAATGGAAAA      1680
CHUDEA1_370         GAATGCAATACACAACAATTGCTTATATCGTTGGGTAAGCAAAAAAAAGAAAATGGAAAA      1680
GY17_00001034       GAATGCAATACACAACAATTGCTTATATCGTTGGGTAAGCAAAAAAAAGAAAATGGAAAA      1680
                    ************************************************************

CPATCC_0039030      GATGAATTCAAGTTTGTTAGAGGGTTTGTTTTTAACGATTCCGTTGAATCAAGATCTAGT      1740
CHUDEA1_370         GATGAATTCAAATTTGTTAGAGGGTTTGTTTTAAACGATTCCGTTGAATCAAGATCTAGT      1740
GY17_00001034       GATGAATTCAAATTTGTTAGAGGGTTTGTTTTAAACGATTCCGTTGAATCAAGATCTAGT      1740
                    *********** ******************** ***************************

CPATCC_0039030      CGTGGATTTAAGGTGAATTTCAAATACAAGAATTCATCCATTAATATAGCTTCCAAATCA      1800
CHUDEA1_370         CGAGGATTTAAGGTAAATTTCAAATACAAGAATTCATCCATTAATATAGCTTCCAAATCA      1800
GY17_00001034       CGAGGATTTAAGGTAAATTTCAAATACAAGAATTCATCCATTAATATAGCTTCCAAATCA      1800
                    ** *********** *********************************************

CPATCC_0039030      AATTCCAACCCAAACTTTATTATTCATACTTGTATTGCAAACATTAAAAATAAACACACG      1860
CHUDEA1_370         AATTCAAACCCAAACTTTATTATTCATACTTGTATTGCAAACATTAGAAATAAACACACA      1860
GY17_00001034       AATTCAAACCCAAACTTTATTATTCATACTTGTATTGCAAACATTAGAAATAAACACACA      1860
                    ***** **************************************** ************ 

CPATCC_0039030      AATGGAATTTGTCAGTGCGGATTCATTAAATTCCCTGATAATAATTCCAACTCTAATACC      1920
CHUDEA1_370         AATGGAATTTGTCAGTGTGGATTCATTAAATTCCCTGATAATAATTCCAACTCTAATACC      1920
GY17_00001034       AATGGAATTTGTCAGTGTGGATTCATTAAATTCCCTGATAATAATTCCAACTCTAATACC      1920
                    ***************** ******************************************

CPATCC_0039030      GCTTTAATCGTGCATTCATCAAGTTCTGATTATTTATGTAATCAACTTTACTTAAGAAAA      1980
CHUDEA1_370         GCTTTAATAGTGCATTCATCAAGTTCTGATTATTTATGTAATCAACTTTACTTAAGGAAA      1980
GY17_00001034       GCTTTAATAGTGCATTCATCAAGTTCTGATTATTTATGTAATCAACTTTACTTAAGGAAA      1980
                    ******** *********************************************** ***

CPATCC_0039030      AATGAATTCCTTCCTTATTCCGAAATATACCTTAATATAGAACGGAACTCTAAAAACAAG      2040
CHUDEA1_370         AATGACTTCCCTCCATACTCAGAAATATACCTTGATATAGAACGGAACTCTAAAAACAAG      2040
GY17_00001034       AATGACTTCCCTCCATACTCAGAAATATACCTTGATATAGAACGGAACTCTAAAAACAAG      2040
                    ***** **** *** ** ** ************ **************************

CPATCC_0039030      CAAAGTATCATCTTCCTTAATAGCGAGCCAGCTGACTTTGGAAAAAATCTGACATATTTA      2100
CHUDEA1_370         CAAAGTATCATCTTCCTTAATAACGAACCAGCTGACTTTGGAAGAAATCTTACATATTTA      2100
GY17_00001034       CAAAGTATCATCTTCCTTAATAACGAACCAGCTGACTTTGGAAGAAATCTTACATATTTA      2100
                    ********************** *** **************** ****** *********
```

CPATCC_0028230

```
CPATCC_0028230          GGCGCAAGAGTTGAAAATCTGTATTGCTGGTATGAGGTTATCGTACCTGCTAAGCCTCAA   1020
GY17_00002211           GGCACAAGAATTGAAAATCTGTATAGCTGGTATGAGGTTGTTGTGCCTCCTAAACCTCAA   1020
CHUDEA2_2560            GGCACAAGAATTGAAAATCTGTATAGCTGGTATGAGGTTGTTGTGCCTCCTAAACCTCAA   1020
ChTU502y2012_366g0190   GGCACAAGAATTGAAAATCTGTATAGCTGGTATGAGGTTGTTGTGCCTCCTAAACCTCAA   1020
Chro.20273              GGCACAAGAATTGAAAATCTGTATAGCTGGTATGAGGTTGTTGTGCCTCCTAAACCTCAA   1020
                        *** ***** ************** ************** * ** *** **** ******

CPATCC_0028230          TATTGTTCAACAATGAAACTTCCACTGAATGGAGTCGGTAAAAAAAATCCTGTTTTACAA   1080
GY17_00002211           TATTGTTCAACAATGAAACTTTCACTGAATCGAGTCGGTGAAAAAGGTCCTGCTTCACAA   1080
CHUDEA2_2560            TATTGTTCAACAATGAAACTTTCACTGAATCGAGTCGGTGAAAAAGGTCCTGCTTCACAA   1080
ChTU502y2012_366g0190   TATTGTTCAACAATGAAACTTTCACTGAATCGAGTCGGTGAAAAAGGTCCTGCTTCACAA   1080
Chro.20273              TATTGTTCAACAATGAAACTTTCACTGAATCGAGTCGGTGAAAAAGGTCCTGCTTCACAA   1080
                        ********************* ******** ******** *****  ***** ** ****

CPATCC_0028230          AGTGGAAGTAAAGGAACTAATTTAGAAACTTCCTATGAAGATAAATTTAAAAGCCATCTT   1140
GY17_00002211           AGTGAAAGTAA---AACTGATTTAGAAACTTCCCATCCAGATAAATTTAATAGTCATCTT   1137
CHUDEA2_2560            AGTGAAAGTAA---AACTGATTTAGAAACTTCCCATCCAGATAAATTTAATAGTCATCTT   1137
ChTU502y2012_366g0190   AGTGAAAGTAA---AACTGATTTAGAAACTTCCCATCCAGATAAATTTAATAGTCATCTT   1137
Chro.20273              AGTGAAAGTAA---AACTGATTTAGAAACTTCCCATCCAGATAAATTTAATAGTCATCTT   1137
                        **** ******   **** ************** **  ************ ** ******

CPATCC_0028230          TCCATTTTATCTAGAAGCCCGAAGACTATACCTAGCGAAACTAAACAAAATAAACTTAGA   1200
GY17_00002211           TCTGTTTTATCTAGAGGCCCGAAGACTAGACCTAGCGAAATCAAACAAAATAAATTTAGA   1197
CHUDEA2_2560            TCTGTTTTATCTAGAGGCCCGAAGACTAGACCTAGCGAAATCAAACAAAATAAATTTAGA   1197
ChTU502y2012_366g0190   TCTGTTTTATCTAGAGGCCCGAAGACTAGACCTAGCGAAATCAAACAAAATAAATTTAGA   1197
Chro.20273              TCTGTTTTATCTAGAGGCCCGAAGACTAGACCTAGCGAAATCAAACAAAATAAATTTAGA   1197
                        **  *********** ************ ***********  ************ *****

CPATCC_0028230          TGGAAAGTAGAAAATACAGGCGCAGAGAGATCAAGTTCGGATAACAGAAAAAACGGTCCC   1260
GY17_00002211           TGTAAAGTAGAAACTGCAGGCGCAGAGAGATCAAATTCAGATAGCAAAGAAAATGGTTCC   1257
CHUDEA2_2560            TGTAAAGTAGAAACTGCAGGCGCAGAGAGATCAAATTCAGATAGCAAAGAAAATGGTTCC   1257
ChTU502y2012_366g0190   TGTAAAGTAGAAACTGCAGGCGCAGAGAGATCAAATTCAGATAGCAAAGAAAATGGTTCC   1257
Chro.20273              TGTAAAGTAGAAACTGCAGGCGCAGAGAGATCAAATTCAGATAGCAAAGAAAATGGTTCC   1257
                        ** ********** * ****************** *** **** ** * **** *** **

CPATCC_0028230          AGATCTGGAATGCTTGGAGGGCCTATTCAACATAGTGCTGAAGGAATGGATACTTACCAT   1320
GY17_00002211           GGATCTGGAATGCTTGGAGGGCCTATTCAACATAGTACTGAAGGAATGGACACTTACCGC   1317
CHUDEA2_2560            GGATCTGGAATGCTTGGAGGGCCTATTCAACATAGTACTGAAGGAATGGATACTTACCGC   1317
ChTU502y2012_366g0190   GGATCTGGAATGCTTGGAGGGCCTATTCAACATAGTACTGAAGGAATGGATACTTACCGC   1317
Chro.20273              GGATCTGGAATGCTTGGAGGGCCTATTCAACATAGTACTGAAGGAATGGATACTTACCGC   1317
                         *********************************** ************* *******

CPATCC_0028230          GCAACTTATGTTGAGGTTGATGATTCCTCTGATGAGGAGGACACAATTAATCCAGTTGGT   1380
GY17_00002211           GCAACTTATGTTGAGGTTGATGATTCCTCTGATGAGGGGGACACAAATAATCCAATTGAT   1377
CHUDEA2_2560            GCAACTTATGTTGAGGTTGATGATTCCTCTGACGAGGGGGACACAAATAATCCAATTGAT   1377
ChTU502y2012_366g0190   GCAACTTATGTTGAGGTTGATGATTCCTCTGACGAGGGGGACACAAATAATCCAATTGAT   1377
Chro.20273              GCAACTTATGTTGAGGTTGATGATTCCTCTGATGAGGGGGACACAAATAATCCAATTGAT   1377
                        ******************************** **** ******** ******* *** *

CPATCC_0028230          CCTCCGGACGCAGACCCAAGCGTCGCACAAATTTTGAAGCTATTAGAGCTTGATATAGAT   1440
GY17_00002211           CCTCCTAATGCAAACTCAAGCGTCTCAAACATTTTGAAGCTATTACGGCTTGATATAGAT   1437
CHUDEA2_2560            CCTCCTAATGCAAACTCAAGCGTCTCAAACATTTTGAAGCTATTACGGCTTGATATAGAT   1437
ChTU502y2012_366g0190   CCTCCTAATGCAAACTCAAGCGTCTCAAACATTTTGAAGCTATTACGGCTTGATATAGAT   1437
Chro.20273              CCTCCTAATGCAAACTCAAGCGTCTCAAACATTTTGAAGCTATTACGGCTTGATATAGAT   1437
                        *****  * *** ** ******** ** * ***************  *************

CPATCC_0028230          CTGAAAACAAATGAAGAAGCTGTGGATCATAATCAAAATGGCACCGGTAATAGACGTACT   1500
GY17_00002211           CTGAAAACAAGTGAAGAAGCTGGGAATCGTAATCAAAATAATACCGGTGATAGATATCTT   1497
CHUDEA2_2560            CTGAAAACAAGTGAAGAAGCTGGGAATCGTAATCAAAATAATACCGGTGATAGATATCTT   1497
ChTU502y2012_366g0190   CTGAAAACAAGTGAAGAAGCTGGGAATCGTAATCAAAATAATACCGGTGATAGATATCTT   1497
Chro.20273              CTGAAAACAAGTGAAGAAGCTGGGAATCGTAATCAAAATAATACCGGTGATAGATATCTT   1497
                        ********** *********** * *** **********   ****** *****  *  *
```

CPATCC_0031960

```
CPATCC_0031960       AACTTATCATTTATTTATATTATTTTGAATGTTTAGATTGGATTACTCCAGAACAGGAAA      300
CHUDEA3_990          AACTTATCATTTATTTATATTATTTTGAATGTTTAGATTGGATTACTCCAGAACAGGAAA      300
GY17_00002623        AACTTATCATTTATTTATATTATTTTGAATGTTTAGATTGGATTACTCCAGAACAGGAAA      300
                     ************************************************************

CPATCC_0031960       AGACATTACTAGAGAATATCTCAAGATCCTCATTTCTAAATGTAAAATTGAACGGAAGAC      360
CHUDEA3_990          AGATATTACTAGAGAATATCTCAAGATCCTCATTTCTAAATGTAAAATTGAACGGAAGAC      360
GY17_00002623        AGATATTACTAGAGAATATCTCAAGATCCTCATTTCTAAATGTAAAATTGAACGGAAGAC      360
                     *** ********************************************************

CPATCC_0031960       AAACTCAAGTATGGGGAGGAACAGTCTCTGAATCAGGAATTGTTAATCAGAAAGATTTAC      420
CHUDEA3_990          AAACTCAAGTATGGGGAGGAACAGTCTCTGAATCAGGAATTGTTGATCAGAAAGATTTAC      420
GY17_00002623        AAACTCAAGTATGGGGAGGAACAGTCTCTGAATCAGGAATTGTTGATCAGAAAGATTTAC      420
                     ******************************************** ***************

CPATCC_0031960       CTGAATGGTTAGAATCTATTTCTCAATCTTTAGTAGATTACAATATATTTTCAAAAGAAG      480
CHUDEA3_990          CTGAATGGTTAGAATCTATTTCTCAATCTTTAGTAGATTACAATATATTTTCAAAAGAAG      480
GY17_00002623        CTGAATGGTTAGAATCTATTTCTCAATCTTTAGTAGATTACAATATATTTTCAAAAGAAG      480
                     ************************************************************

CPATCC_0031960       AAACTCCAAACCATGTACTAATCAATCAATATGAACAATACAAAGGGATTCTTCCTCACA      540
CHUDEA3_990          AAACTCCAAACCATGTACTAATCAATCAGTATGAACAATACAAAGGGATTCTTCCTCACA      540
GY17_00002623        AAACTCCAAACCATGTACTAATCAATCAGTATGAACAATACAAAGGGATTCTTCCTCACA      540
                     **************************** *******************************

CPATCC_0031960       AGGATGGCCCTCTTTATTACCCAAGAGTCGCAATCATCTCATTAGAATCTGACACTTTAT      600
CHUDEA3_990          AGGATGGCCCCCTCTATTACCCAAGAGTTGCAATCATCTCATTAGAATCTGACACTTTAT      600
GY17_00002623        AGGATGGCCCCCTCTATTACCCAAGAGTTGCAATCATCTCATTAGAATCTGACACTTTAT      600
                     ********** ** ************** *******************************

CPATCC_0031960       TCGATTTTTGGAATCCCTCTCTTGATACTCAAGAAAACAAATTCCCTCTATTCTCACTTA      660
CHUDEA3_990          TCGACTTTTGGAATCCCTCTCTTGATATTCAAGAAAACAAATTCCCTCTATTCTCACTTA      660
GY17_00002623        TCGACTTTTGGAATCCCTCTCTTGATATTCAAGAAAACAAATTCCCTCTATTCTCACTTA      660
                     **** ********************** ********************************

CPATCC_0031960       TTGTCCCCAAACTCAGCCTACTAGTCTTTCAAGATCTATGCTATACTCAACTTCTACATG      720
CHUDEA3_990          TTGTCCCCAAACTCAGCCTACTAGTCTTTCAAGATCTATGCTATACTCAACTTCTACATG      720
GY17_00002623        TTGTCCCCAAACTCAGCCTACTAGTCTTTCAAGATCTATGCTATACTCAACTTCTACATG      720
                     ************************************************************

CPATCC_0031960       GAATTTCATCCAGGTAAGCTTTACCTATTTACCAGTTCTATTTCTAATATTTATAATTCT      780
CHUDEA3_990          GAATTTCATCCAGGTGAGCTTTACCTATTTACCAATTCTATTTCTAATATTTATAATTCT      780
GY17_00002623        GAATTTCATCCAGGTGAGCTTTACCTATTTACCAATTCTATTTCTAATATTTATAATTCT      780
                     *************** ****************** *************************

CPATCC_0031960       ACTCAAATCTCGAATTTCAGGCACCAAGACGATCTAGAAAACCATAACGTTATTAATATT      840
CHUDEA3_990          ACTCAAATCTCGGATTTCAGGCACCAAGACAATCTAGAAAACCATAACGTCATTAATATT      840
GY17_00002623        ACTCAAATCTCGGATTTCAGGCACCAAGACAATCTAGAAAACCATAACGTCATTAATATT      840
                     ************ ***************** ******************* *********

CPATCC_0031960       AAAGATTTTCCTCATTTAAGCCCTAACTCTACCATTAAAAGAGGATTTAGAACGAGCCTA      900
CHUDEA3_990          AAAGAATTTCCTCATTTAAGCCCTAACTCTATCATTAAAAGAGGATTTAGAACGAGCCTA      900
GY17_00002623        AAAGAATTTCCTCATTTAAGCCCTAACTCTATCATTAAAAGAGGATTTAGAACGAGCCTA      900
                     ***** ************************* ****************************
```

CPATCC_0021750

```
CPATCC_0021750              GGGAATGAAGCAAGCAATGCAATTGGAACTGAAACTTCAACACCCCAGGAAGGACATATA      2220
ChTU502y2012_422g0160       GGGAATGAAGCAAGCAATGTAATTGAAACTGAAACTTCAACAGCCCAGGAGGGACATATA      2220
CHUDEA4_4490                GGGAATGAAGCAAGCAATGTAATTGAAACTGAAACTTCAACAGCCCAGGAGGGACATATA      2220
Chro.40510                  GGGAATGAAGCAAGCAATGTAATTGAAACTGAAACTTCAACAGCCCAGGAGGGACATATA      870
GY17_00003410               GGGAATGAAGCAAGCAATGTAATTGAAACTGAAACTTCAACAGCCCAGGAGGGACATATA      2220
                            ******************* ***** **************** ******* *********

CPATCC_0021750              TCAAGATCAAAAACAAGATCAAAATCAAAATCAAGATCAAAATCAAGATCAAAATCAAGA      2280
ChTU502y2012_422g0160       TCAAGATCAAAATCAAGAGCAAGATCAAAATCAAGATCAAAATCAAGAGCAAGATCAAAA      2280
CHUDEA4_4490                TCAAGATCAAAATCAAGAGCAAGATCAAAATCAAGATCAAAATCAAGAGCAAGATCAAAA      2280
Chro.40510                  TCAAGATCAAAATCAAGAGCAAGATCAAAATCAAGATCAAAATCAAGAGCAAGATCAAAA      930
GY17_00003410               TCAAGATCAAAATCAAGAGCAAGATCAAAATCAAGATCAAAATCAAGAGCAAGATCAAAA      2280
                            ************ ***** *** ************************* *** ***** *

CPATCC_0021750              TCAAAATCAAAATCAAGAGCAAGATCAAAATCAAGAACAAGAGCAAGATCAAAATCAAGA      2340
ChTU502y2012_422g0160       TCAAGATCAAAAT-----------------------------------------------      2293
CHUDEA4_4490                TCAAGATCAAAAT-----------------------------------------------      2293
Chro.40510                  TCAAGATCAAAAT-----------------------------------------------      943
GY17_00003410               TCAAGATCAAAAT-----------------------------------------------      2293
                            **** ********

CPATCC_0021750              TCAAGATCAAGAACAAGATCAAGAACAAGATCAAGATCAAGATCAAGAACAAGATCAAGA      2400
ChTU502y2012_422g0160       -------------------------CAAGAGTAAGATCAAGAGCAAGAACAAGATCAAGA      2328
CHUDEA4_4490                -------------------------CAAGAGTAAGATCAAGAGCAAGAACAAGATCAAGA      2328
Chro.40510                  -------------------------CAAGAGTAAGATCAAGAGCAAGAACAAGATCAAGA      978
GY17_00003410               -------------------------CAAGAGTAAGATCAAGAGCAAGAACAAGATCAAGA      2328
                                                     *****  ********** *****************

CPATCC_0021750              ACAAGGTCAAGAACAAGATCAAGAACAAGATCAAGATCAAACTTAGATTCTGAACTCGAT      2460
ChTU502y2012_422g0160       ACAAGATCAAGAAAAAGATCAAGAACAAGATCAAGATCAAACATAGATTCTGAACTCGAT      2388
CHUDEA4_4490                ACAAGATCAAGAAAAAGATCAAGAACAAGATCAAGATCAAACATAGATTCTGAACTCGAT      2388
Chro.40510                  ACAAGATCAAGAAAAAGATCAAGAACAAGATCAAGATCAAACATAGATTCTGAACTCGAT      1038
GY17_00003410               ACAAGATCAAGAAAAAGATCAAGAACAAGATCAAGATCAAACATAGATTCTGAACTCGAT      2388
                            ***** ******* **************************** *****************

CPATCC_0021750              TCAGGAACTAGTAAAACAGAGTGTACAGAAGGTAGTTCTGTACTTGCTGAACCTTTTAAA      2520
ChTU502y2012_422g0160       TCAAGAACTAGTAAAACAGAGTGTACAGAAGGTAGTTCTGTACTTGTTGAACCTTTTAAA      2448
CHUDEA4_4490                TCAAGAACTAGTAAAACAGAGTGTACAGAAGGTAGTTCTGTACTTGTTGAACCTTTTAAA      2448
Chro.40510                  TCAAGAACTAGTAAAACAGAGTGTACAGAAGGTAGTTCTGTACTTGTTGAACCTTTTAAA      1098
GY17_00003410               TCAAGAACTAGTAAAACAGAGTGTACAGAAGGTAGTTCTGTACTTGTTGAACCTTTTAAA      2448
                            *** ****************************************** *************

CPATCC_0021750              TCAAAATTTTTGAAGATGTTTGTGAAAGAGCCCTTGGAAGATTCTACTCCTTCAGTAATG      2580
ChTU502y2012_422g0160       TCAAAATTTTTGAAGATGTTTGTGAAAAAGCCCTTAGAAGATTCTACTCCTTCAGTAATG      2508
CHUDEA4_4490                TCAAAATTTTTGAAGATGTTTGTGAAAAAGCCCTTAGAAGATTCTACTCCTTCAGTAATG      2508
Chro.40510                  TCAAAATTTTTGAAGATGTTTGTGAAAAAGCCCTTAGAAGATTCTACTCCTTCAGTAATG      1158
GY17_00003410               TCAAAATTTTTGAAGATGTTTGTGAAAAAGCCCTTAGAAGATTCTACTCCTTCAGTAATG      2508
                            *************************** ******* ************************

CPATCC_0021750              AAATTTTTCAGTATTTCATATCCAAATGAGCCAACTGTTACAGTAATGTCAGAGGCTAAA      2640
ChTU502y2012_422g0160       AAATTTTTCAGTGTTTCATATCCAAATGAGTCAACTGTTACAGTAATGTCACAGGCTAAA      2568
CHUDEA4_4490                AAATTTTTCAGTGTTTCATATCCAAATGAGTCAACTGTTACAGTAATGTCACAGGCTAAA      2568
Chro.40510                  AAATTTTTCAGTGTTTCATATCCAAATGAGTCAACTGTTACAGTAATGTCACAGGCTAAA      1218
GY17_00003410               AAATTTTTCAGTGTTTCATATCCAAATGAGTCAACTGTTACAGTAATGTCACAGGCTAAA      2568
                            ************ ***************** ******************** ********

CPATCC_0021750              GAAATTTTTAATGAAGTTCAAAATTGCTTTTTGGAAACGGCCAGATTACATAATGAAGTT      2700
ChTU502y2012_422g0160       GAAATTTTTAATGAAGTTCAAAATTGCTTTTTGGAAACGGCCAGATTACATAATGAAGTT      2628
CHUDEA4_4490                GAAATTTTTAATGAAGTTCAAAATTGCTTTTTGGAAACGGCCAGATTACATAATGAAGTT      2628
Chro.40510                  GAAATTTTTAATGAAGTTCAAAATTGCTTTTTGGAAACGGCCAGATTACATAATGAAGTT      1278
GY17_00003410               GAAATTTTTAATGAAGTTCAAAATTGCTTTTTGGAAACGGCCAGATTACATAATGAAGTT      2628
                            ************************************************************
```

CPATCC_0024650

```
CPATCC_0024650      ATGTTTAGATATTGTAGTAAAATCACCGAAAGAGTGTTATACTCTACTATAAAGAATGTT      60
Chro.50092          ATGTTTAGATATTGTAGTAAAATCACCGAAAGAGTGTTATACTCTACTATAAAGAATGTT      60
                    ************************************************************

CPATCC_0024650      ATGAATAAGATATTCACAAATTTTGAACAAGAGCAACTTTCCATCTCACTTTTAGGTGGA      120
Chro.50092          ATGAATAAGATATTTACAAATTTTGAACAAGAACAACTTTCCATCTCACTTTTAGGTGGA      120
                    ************** ***************** ***************************

CPATCC_0024650      AGCTTGGAAATAAGGGATTTAAATATTAGAAAAGAACTTTTTGATGATTTATCTTTCCCA      180
Chro.50092          AGCTTGGAAATAAGGGATTTAAATATTAGAAAAGAACTTTTTGATAATTTATCTTTCCCA      180
                    ********************************************* **************

CPATCC_0024650      ATCAGTTTAAGTGATGGAATTGTTGGAAAAGTTAATATTGATGTTATCTGGAGAAAAATA      240
Chro.50092          ATCAGTTTAAGTGATGGAATTGTTGGAAAAGTTAATATTGATGTTATCTGGAGAAAAATA      240
                    ************************************************************

CPATCC_0024650      TTTACCCAAGAGTTTGTGAAAATCACCTTGGATGATGTATATGTTATTTTTAATACTACT      300
Chro.50092          TTTACCCAAGAGTTTGTAAAAATCACCTTGGATGATGTATATGTTATTTTTAATACTACT      300
                    ***************** ******************************************

CPATCC_0024650      GATATGAAAAAATTGGAATGTTGAAATGTTTGAAAAAAACTGGAAAAGGGTTAAAGCTAAT      360
Chro.50092          GATATGAAAAAATTGGAATGTTGAAATGTTTGAAAAAAACTGGAAAAGGTTTAAAGCTAAT      360
                    ************************************************ ***********

CPATCC_0024650      CTATTAAAACAAGATGAATTTATTACTTTCTTAAAAAGTGCTATGGCTTCCAATTTTCTC      420
Chro.50092          TTATTAAAACAAGATGAATTTATTACTTTCTTAAAAAGTGCTATGGCTTCCAATTTTCTC      420
                     ***********************************************************

CPATCC_0024650      AAACAAATTGGACACTTTTTTATATCAAAGATTCAGTTTGAAATTAAAAACATCAATTTT      480
Chro.50092          AAACAAATTGGACACTTTTTTATATCAAAGATTCAGTTTGAAATTAAAAACATCAATTTT      480
                    ************************************************************

CPATCC_0024650      AGAATTGAAAATTTGTTATTCCACTATATGAAGAGATTTGTTATTGGACTAAGTATTGAT      540
Chro.50092          AGAATTGAAAATTTGTTATTCCACTATATGAAGAGATTTGTTATTGGACTAAGTATTGAT      540
                    ************************************************************

CPATCC_0024650      AAAATTTCCAGTCGAAATTGTAATGAATATTGGATTCCAGTAGATAAATCTGGAGGAGCC      600
Chro.50092          AAAATTTCCAGTCGAAATTGTAATGAATATTGGATTCCAGTAGATAAATCTGGAGGAGCC      600
                    ************************************************************

CPATCC_0024650      ATTTTAGGCCGTAATACAGACAAAATTAATGGATCATCAACTTCAAATATAGCTTTTGAT      660
Chro.50092          ATTTTAGGCCGTAATACAGACAAAATTAATGGATCATCAACTTCTACTGTAGCTTTTGAT      660
                    ******************************************** * * ***********
```

CPATCC_0012400

```
CPATCC_0012400           TTTGACTCTATGAATGAATCAATTAGATTACCTACACGTCCTTATGGCCCTGGTGAAAAG    1140
Chro.60121               TTTGACTCTATGAATGAATCAATTAGATTACCTACACGTCCTTATGGCCCTGGTGAAAAG    1140
CHUDEA6_940              TTTGACTCTATGAATGAATCAATTAGATTACCTACACGTCCTTATGGCCCTGGTGAAAAG    1140
GY17_00000093            TTTGACTCTATGAATGAATCAATTAGATTACCTACACGTCCTTATGGCCCTGGTGAAAAG    1140
ChTU502y2012_406g0205    TTTGACTCTATGAATGAATCAATTAGATTACCTACACGTCCTTATGGCCCTGGTGAAAAG    1140
                         ************************************************************

CPATCC_0012400           TTACCTCCACATCTATCTCCATTTGTGGATGATAGTACCCAAGGTTATATTCCAACTCAG    1200
Chro.60121               TTACCCCCACATCTATCTCCATTTGTGGATGATAGTACCCAAGGTTATATTCCAACTCAG    1200
CHUDEA6_940              TTACCCCCACATCTATCTCCATTTGTGGATGATAGTACCCAAGGTTATATTCCAACTCAG    1200
GY17_00000093            TTACCCCCACATCTATCTCCATTTGTGGATGATAGTACCCAAGGTTATATTCCAACTCAG    1200
ChTU502y2012_406g0205    TTACCCCCACATCTATCTCCATTTGTGGATGATAGTACCCAAGGTTATATTCCAACTCAG    1200
                         ***** ******************************************************

CPATCC_0012400           AGACAAGTTCTTGATGAAATTAAGGAATCCAATAGTCATAAATCTCAATCTTGCTTATCT    1260
Chro.60121               AGACAAGTTCTTGATGAAATTAAGGAATCCAATACTCATAAATCTCAATCTTACTTATCT    1260
CHUDEA6_940              AGACAAGTTCTTGATGAAATTAAGGAATCCAATACTCATAAATCTCAATCTTACTTATCT    1260
GY17_00000093            AGACAAGTTCTTGATGAAATTAAGGAATCCAATACTCATAAATCTCAATCTTACTTATCT    1260
ChTU502y2012_406g0205    AGACAAGTTCTTGATGAAATTAAGGAATCCAATACTCATAAATCTCAATCTTACTTATCT    1260
                         ********************************** ***************** *******

CPATCC_0012400           GAAGATGAATCAACAGAAATTGATGAACTTTCAGAACATGATTCTGATATTGAAGTTCAA    1320
Chro.60121               GAAGATGAATCAACAGAAATTGATGAACTTTCAGAACATGATTCTGACATTGAAGTTCAA    1320
CHUDEA6_940              GAAGATGAATCAACAGAAATTGATGAACTTTCAGAACATGATTCTGACATTGAAGTTCAA    1320
GY17_00000093            GAAGATGAATCAACAGAAATTGATGAACTTTCAGAACATGATTCTGACATTGAAGTTCAA    1320
ChTU502y2012_406g0205    GAAGATGAATCAACAGAAATTGATGAACTTTCAGAACATGATTCTGACATTGAAGTTCAA    1320
                         *********************************************** ************

CPATCC_0012400           CAAGCTAGAGAAGATGCCTACTTTGATTCTATTGAGAGAGAACAATCATTATCAACCTCA    1380
Chro.60121               CAAGCTAGAGAAGATGCTTACTTTGATTCTATTGAGAGAGAACAATCATTATCCACCTCA    1380
CHUDEA6_940              CAAGCTAGAGAAGATGCTTACTTTGATTCTATTGAGAGAGAACAATCATTATCCACCTCA    1380
GY17_00000093            CAAGCTAGAGAAGATGCTTACTTTGATTCTATTGAGAGAGAACAATCATTATCCACCTCA    1380
ChTU502y2012_406g0205    CAAGCTAGAGAAGATGCTTACTTTGATTCTATTGAGAGAGAACAATCATTATCCACCTCA    1380
                         ***************** *********************************** ******

CPATCC_0012400           AATGAAATTGATTCAATTGATAACGAGCATAAAGATTCATCCAATTTTACAACAGAATCT    1440
Chro.60121               AATGAAATTGATTCAATTGATACTGAGCATAAAGATTCATCCAATTTTACAACAGAATCT    1440
CHUDEA6_940              AATGAAATTGATTCAATTGATACTGAGCATAAAGATTCATCCAATTTTACAACAGAATCT    1440
GY17_00000093            AATGAAATTGATTCAATTGATACTGAGCATAAAGATTCATCCAATTTTACAACAGAATCT    1440
ChTU502y2012_406g0205    AATGAAATTGATTCAATTGATACTGAGCATAAAGATTCATCCAATTTTACAACAGAATCT    1440
                         **********************  ************************************

CPATCC_0012400           GAATTAACTAATACAAAAGATAAAGTAAATATTGCTAGAAAGCTCGCGAAGAAACGTAAA    1500
Chro.60121               GAATTAACTAATACAAAAGATAAAGTAAATATTGCTAGAAAGCTCGCAAAGAAACGTAGA    1500
CHUDEA6_940              GAATTAACTAATACAAAAGATAAAGTAAATATTGCTAGAAAGCTCGCAAAGAAACGTAGA    1500
GY17_00000093            GAATTAACTAATACAAAAGATAAAGTAAATATTGCTAGAAAGCTCGCAAAGAAACGTAGA    1500
ChTU502y2012_406g0205    GAATTAACTAATACAAAAGATAAAGTAAATATTGCTAGAAAGCTCGCAAAGAAACGTAGA    1500
                         *********************************************** ********** *

CPATCC_0012400           GAAGAGGAACAAAGAGAACAACAAAAGACTCTACTCAAAAAGAAGCATAAGAGACTTTTA    1560
Chro.60121               GAAGAAGAACAAAGAGAACAACAAAAGACTCTACTCAAAAAGAAGCATAAGAAACTTTTA    1560
CHUDEA6_940              GAAGAAGAACAAAGAGAACAACAAAAGACTCTACTCAAAAAGAAGCATAAGAGACTTTTA    1560
GY17_00000093            GAAGAAGAACAAAGAGAACAACAAAAGACTCTACTCAAAAAGAAGCATAAGAGACTTTTA    1560
ChTU502y2012_406g0205    GAAGAAGAACAAAGAGAACAACAAAAGACTCTACTCAAAAAGAAGCATAAGAGACTTTTA    1560
                         ***** ********************************************** *******

CPATCC_0012400           CAAAGAATTGAATATTCTAACAAAATCGCTTCGGAGAAAGCAGAAAGGTTGGAATCTCGA    1620
Chro.60121               CAAAGAATTGAATATTCGAATAAAATTGCTTCAGAGAAGGCAGAAAGGTTGGAATCTCGA    1620
CHUDEA6_940              CAAAGAATTGAATATTCGAATAAAATTGCTTCAGAGAAGGCAGAAAGGTTGGAATCTCGA    1620
GY17_00000093            CAAAGAATTGAATATTCGAATAAAATTGCTTCAGAGAAGGCAGAAAGGTTGGAATCTCGA    1620
ChTU502y2012_406g0205    CAAAGAATTGAATATTCGAACAAAATTGCTTCAGAGAAGGCAGAAAGGTTGGAATCTCGA    1620
                         ***************** ** ***** ***** ***** *********************
```

CPATCC_0007000

```
CPATCC_0007000      CGTGTTCTTTTGGGAGTTTCTGAATCATTTTCAGCTAGGAAACTCTTTGAGCCAATACAA
Chro.70152          CGTTTTCTTTTGGGAGTTTCTGAATCATTTTCAGCTAGGAAACTCTTTGAGCCAATACAA
CHUDEA7_1270        CGTTTTCTTTTGGGAGTTTCTGAATCATTTTCAGCTAGGAAACTCTTTGAGCCAATACAA
                    *** ********************************************************

CPATCC_0007000      ATTTTAAAGTCAGCTAAAGATCTCAACAAGATTTCTTCTGATTTCCTACGAAAGATCTCA
Chro.70152          ATTTTAAAGTCAGCTAAAGATCTCAACAAGATTTCTTCTGATTTCCTACGAAAGATCTCA
CHUDEA7_1270        ATTTTAAAGTCAGCTAAAGATCTCAACAAGATTTCTTCTGATTTCCTACGAAAGATCTCA
                    ************************************************************

CPATCC_0007000      GTTCTCAAGTTATGTGTTTACGATACTAATCCTGAATTCCTATTCTATGAGTATTACTCA
Chro.70152          GTTCTCAAGTTATGTGTTTACGTTACTAATTCTGATTTCCTATTCTATGAGTATTACTCA
CHUDEA7_1270        GTTCTCAAGTTATGTGTTTACGTTACTAATTCTGATTTCCTATTCTATGAGTATTACTCA
                    ********************** ******* **** ************************

CPATCC_0007000      GCTGAGATTATTTTGTCCCTCCAAAGATCTGGATTGAAAATTACAGAAACAGACATGTTT
Chro.70152          GCTGAGATGATTTTATCTTTCCAAAGATCTGGATTAAAACTTACAGAAACAGACATGTTT
CHUDEA7_1270        GCTGAGATGATTTTATCTTTCCAAAGATCTGGATTAAAACTTACAGAAACAGACATGTTT
                    ******** ***** **  **************** *** ********************

CPATCC_0007000      AGTCTCCTTTTGAATCATATCTTAATTAACTTTGCCAGAGAATTCATCTCAAAGCCACAA
Chro.70152          AGTCTCCTTTTGAATCATATCTTAATTAACTCTGCCAGAGAATTCATCTCAAAGCCACAA
CHUDEA7_1270        AGTCTCCTTTTGAATCATATCTTAATTAACTCTGCCAGAGAATTCATCTCAAAGCCACAA
                    ***************************** ******************************

CPATCC_0007000      TTAATCGCAGATTGCTTCTCAAACTTTAAGTTTAAACAAAGGCCAAGCTATTTCTCAACA
Chro.70152          TTAATCATAGATTGCTTCTCAGACTTTAAGTTTAAACAAAGGCCAAGCTATTTCTCAACA
CHUDEA7_1270        TTAGTCATAGATTGCTTCTCAGACTTTAAGTTTAAACAAAGGCCAAGCTATTTCTCAACA
                    *** **  ************* **************************************

CPATCC_0007000      GTCCCAGAGTTTGGACATGTATCCCAAAATACTGGTTTAATTACTTCATCATTCAAGAAT
Chro.70152          GTCCCAGAGTTTGGACATGTATCCCAAAATACTGGTTTAATTACTTCATCATTCAAGAAT
CHUDEA7_1270        GTCCCAGAGTTTGGACATGTATCCCAAAATACTGGTTTAATTACTTCATCATTCAAGAAT
                    ************************************************************

CPATCC_0007000      CTTGGATTTACAGGCCTACAAGAATCTTTTGTTCCTCATGTTAACTTATTGGTCTATTAC
Chro.70152          CTTGGATTTACAGGCCTACAAGAGTCTTTTGTTCCTCATGTTAACTTATTGGTCTATTAC
CHUDEA7_1270        CTTGGATTTACAGGCCTACAAGAGTCTTTTGTTCCTCATGTTAACTTATTGGTCTATTAC
                    *********************** ************************************

CPATCC_0007000      CTTAAATCTCTTAACTCCATAGTAAACCATCCAGGAAACAGAATAATCCTTTCAATTCCA
Chro.70152          CTTAAATCTCTTAACTCCATAGTAAACCATCCGGGAAACAGAATAATCCTTTCAATTCCA
CHUDEA7_1270        CTTAAATCTCTTAACTCCATAGTAAACCATCCGGGAAACAGAATAATCCTTTCAATTCCA
                    ******************************** ***************************

CPATCC_0007000      GCTTCTATTAACTTTATGAGTACCAGTATACTACTAAGAGCTTCCAGTTGTAGTCAAGTA
Chro.70152          GCTTCTATTAACTTTATGAGTACCAGTATACTACTAAAAGCTTCCAGTTGTAGTCAAGTA
CHUDEA7_1270        GCTTCTATTAACTTTATGAGTACCAGTATACTACTAAAAGCTTCCAGTTGTAGTCAAGTA
                    ************************************* **********************
```

CPATCC_0001010

```
CPATCC_0001010              GCCATAACTAGAGCAATCCCAGTAACAACACCACATGAACAGGTAATTACTACTGTCGAT        5799
GY17_00002840               GCCATAACTAGAGCAATCCCAGTAACAACACCACAAGAACAGGTAATTAATACTGTGGAT        5763
CHUDEA8_970                 GCCATAACTAGAGCAATCCCAGTAACAACACCACAAGAACAGGTAATTAATACTGTGGAT        5796
ChTU502y2012_409g0080       GCCATAACTAGAGCAATCCCAGTAACAACACCACAAGAACAGGTAATTAATACTGTGGAT        5799
Chro.80116                  GCCATAACTAGAGCAATCCCAGTAACAACACCACAAGAACAGGTAATTAATACTGTGGAT        5799
                            *********************************** ************* ****** ***

CPATCC_0001010              AACACACAAACATATTCTGTTAGAGAGGTTAGAACAGAAGACAATGAAATTCGCCGTTCT        5859
GY17_00002840               AACACACAAACATATTCTGCTAGAGAGGTTAGAACAGAAGACAATGAAATTCGCCATTCT        5823
CHUDEA8_970                 AACACACAAACATATTCTGCTAGAGAGGTTAGAACAGAAGACAATGAAATTCGCCATTCT        5856
ChTU502y2012_409g0080       AACACACAAACATATTCTGCTAGAGAGGTTAGAACAGAAGACAATGAAATTCGCCATTCT        5859
Chro.80116                  AACACACAAACATATTCTGCTAGAGAGGTTAGAACAGAAGACAATGAAATTCGCCATTCT        5859
                            ******************* *********************************** ****

CPATCC_0001010              TCTCAGTATAATAGTGCTAATGTGAAGCCAATAAACTTGCATGAAAACCAAATAGGAACA        5919
GY17_00002840               TCTCAGTATAATAGTGCTAATATGAAGCCAATAAACTTACATGAAAACCAAATAGGAACA        5883
CHUDEA8_970                 TCTCAGTATAATAGTGCTAATATGAAGCCAATAAACTTACATGAAAACCAAATAGGAACA        5916
ChTU502y2012_409g0080       TCTCAGTATAATAGTGCTAATATGAAGCCAATAAACTTACATGAAAACCAAATAGGAACA        5919
Chro.80116                  TCTCAGTATAATAGTGCTAATATGAAGCCAATAAACTTACATGAAAACCAAATAGGAACA        5919
                            ********************* **************** *********************

CPATCC_0001010              GAAATTAACAATACAAATGGAAATTTTGAAACTAGAAGAAAGTCTAGCAACCTTATATTA        5979
GY17_00002840               GAAATTAACAATACAAATGGAAATTTTGAAACTAGAAGAAAGTCTAGCAACCTTATATTA        5943
CHUDEA8_970                 GAAATTAACAATACAAATGGAAATTTTGAAACTAGAAGAAAGTCTAGCAACCTTATATTA        5976
ChTU502y2012_409g0080       GAAATTAACAATACAAATGGAAATTTTGAAACTAGAAGAAAGTCTAGCAACCTTATATTA        5979
Chro.80116                  GAAATTAACAATACAAATGGAAATTTTGAAACTAGAAGAAAGTCTAGCAACCTTATATTA        5979
                            ************************************************************

CPATCC_0001010              ATTAGAGACATTGAGACTCAGAGCCTTAAAGATAAGAATGATAACCACAGTCTTACTATA        6039
GY17_00002840               ATCAGAGACATTGAGACTCAGAGCCTTAAAGATAAGAATGATAACCACAATCTCACTATA        6003
CHUDEA8_970                 ATCAGAGACATTGAGACTCAGAGCCTTAAAGATAAGAATGATAACCACAATCTCACTATA        6036
ChTU502y2012_409g0080       ATCAGAGACATTGAGACTCAGAGCCTTAAAGATAAGAATGATAACCACAATCTCACTATA        6039
Chro.80116                  ATCAGAGACATTGAGACTCAGAGCCTTAAAGATAAGAATGATAACCACAATCTCACTATA        6039
                            ** ********************************************** *** ******

CPATCC_0001010              GAAAAACAAGGAACAGAGGAAGAAAAAAGAAACTCAAATGACTATCATCACCTCCGCCTC        6099
GY17_00002840               GAGAGACAAGGAATAAAAGAAGAAAAAAGAAACTCAAATGACTATCATCACCTCCGTCTC        6063
CHUDEA8_970                 GAGAGACAAGGAATAAAAGAAGAAAAAAGAAACTCAAATGACTATCATCACCTCCGCCTC        6096
ChTU502y2012_409g0080       GAGAGACAAGGAATAAAAGAAGAAAAAAGAAACTCAAATGACTATCATCACCTCCGCCTC        6099
Chro.80116                  GAGAGACAAGGAATAAAAGAAGAAAAAAGAAACTCAAATGACTATCATCACCTCCGTCTC        6099
                            ** * ******** * * ************************************** ***

CPATCC_0001010              CATCATCATATTCCAACCAAAAACCAAGCAAATTTAGTTCCTCATATTGGTAAAGTATGC        6159
GY17_00002840               CATCATCATATTCCAACTAAAAACCAAGCAAGTCTAGTTCCTCATATTGGTAAAGTATGC        6123
CHUDEA8_970                 CATCATCATATTCCAACTAAAAACCAAGCAAGTCTAGTTCCTCATATTGGTAAAGTATGC        6156
ChTU502y2012_409g0080       CATCATCATATTCCAACTAAAAACCAAGCAAGTCTAGTTCCTCATATTGGTAAAGTATGC        6159
Chro.80116                  CATCATCATATTCCAACTAAAAACCAAGCAAGTCTAGTTCCTCATATTGGTAAAGTATGC        6159
                            ***************** ************* * **************************

CPATCC_0001010              GAAGAAAAAGAATCTGAAATTCTTAACAGGGAATTTATACAAAGAAATAACAACCATGAA        6219
GY17_00002840               GAAGAAAAAGAATCTGAAATTTCTAACAAGGAACTTATACAAAGAAATAACAAACATGAA        6183
CHUDEA8_970                 GAAGAAAAAGAATCTGAAATTTCTAACAAGGAACTTATACAAAGAAATAACAAACATGAA        6216
ChTU502y2012_409g0080       GAAGAAAAAGAATCTGAAATTTCTAACAAGGAACTTATACAAAGAAATAACAAACATGAA        6219
Chro.80116                  GAAGAAAAAGAATCTGAAATTTCTAACAAGGAACTTATACAAAGAAATAACAAACATGAA        6219
                            *********************  ***** **** ******************* ******

CPATCC_0001010              TATATTGAAACACAATCTTCACCTCATCAAAACACTAAGAAAATACAAAAAAAGAAGCCT        6279
GY17_00002840               TATATTGAAACACAATCTTCACCTCATCAAAACATTAAGAAAATACAAAAAAAGAAGCCT        6243
CHUDEA8_970                 TATATTGAAACACAATCTTCACCTCATCAAAACATTAAGAAAATACAAAAAAAGAAGCCT        6276
ChTU502y2012_409g0080       TATATTGAAACACAATCTTCACCTCATCAAAACATTAAGAAAATACAAAAAAAGAAGCCT        6279
Chro.80116                  TATATTGAAACACAATCTTCACCTCATCAAAACATTAAGAAAATACAAAAAAAGAAGCCT        6279
                            ********************************** *************************

CPATCC_0001010              CCAATATCTCTTAGGTCTGGCTTCAATTTTAATAATTAA 6318
GY17_00002840               CCAATATCTCTTAGGTCTGGCTTCAATTTTAATAATTAA 6282
CHUDEA8_970                 CCAATATCTCTTAGGTCTGGCTTCAATTTTAATAATTAA 6315
ChTU502y2012_409g0080       CCAATATCTCTTAGGTCTGGCTTCAATTTTAATAATTAA 6318
Chro.80116                  CCAATATCTCTTAGGTCTGGCTTCAATTTTAATAATTAA 6318
                            ***************************************
```
